# CRISPR activation enables high-fidelity reprogramming into human pluripotent stem cells

**DOI:** 10.1016/j.stemcr.2021.12.017

**Published:** 2022-01-20

**Authors:** Joonas Sokka, Masahito Yoshihara, Jouni Kvist, Laura Laiho, Andrew Warren, Christian Stadelmann, Eeva-Mari Jouhilahti, Helena Kilpinen, Diego Balboa, Shintaro Katayama, Aija Kyttälä, Juha Kere, Timo Otonkoski, Jere Weltner, Ras Trokovic

**Affiliations:** 1 Research Programs Unit, Stem cells and Metabolism and Biomedicum Stem Cell Centre, Faculty of Medicine, University of Helsinki, Helsinki 00014, Finland; 2 Department of Biosciences and Nutrition, Karolinska Institutet, Huddinge, Stockholm 14183, Sweden; 3 Helsinki Institute of Life Science (HiLIFE), University of Helsinki, Helsinki 00014, Finland; 4 Faculty of Biological and Environmental Sciences, University of Helsinki, Helsinki 00014, Finland; 5 Institute for Molecular Medicine Finland (FIMM), University of Helsinki, Helsinki 00014, Finland; 6 European Molecular Biology Laboratory, European Bioinformatics Institute, Wellcome Genome Campus, Hinxton, Cambridge CB10 1SD, UK; 7 Centre for Genomic Regulation (CRG), Barcelona Institute of Science and Technology, Barcelona 08003, Spain; 8 Folkhälsan Research Center, Helsinki 00290, Finland; 9 Finnish Institute for Health and Welfare (THL), THL Biobank, Helsinki 00290, Finland; 10 Children’s Hospital, Helsinki University Central Hospital, University of Helsinki, Helsinki 00290, Finland; 11 Department of Clinical Science, Intervention, and Technology, Karolinska Institutet, Huddinge, Stockholm 14186, Sweden; 12 Division of Obstetrics and Gynecology, Karolinska Universitets Sjukhuset, Huddinge, Stockholm 14186, Sweden

**Keywords:** CRISPRa, iPSC, reprogramming, human, LCL, mir-302/367, EEA, microRNA, single-cell RNA sequencing, transcriptomics

## Abstract

Conventional reprogramming methods rely on the ectopic expression of transcription factors to reprogram somatic cells into induced pluripotent stem cells (iPSCs). The forced expression of transcription factors may lead to off-target gene activation and heterogeneous reprogramming, resulting in the emergence of alternative cell types and aberrant iPSCs. Activation of endogenous pluripotency factors by CRISPR activation (CRISPRa) can reduce this heterogeneity. Here, we describe a high-efficiency reprogramming of human somatic cells into iPSCs using optimized CRISPRa. Efficient reprogramming was dependent on the additional targeting of the embryo genome activation-enriched Alu-motif and the miR-302/367 locus. Single-cell transcriptome analysis revealed that the optimized CRISPRa reprogrammed cells more directly and specifically into the pluripotent state when compared to the conventional reprogramming method. These findings support the use of CRISPRa for high-quality pluripotent reprogramming of human cells.

## Highlights

• Efficient CRISPRa reprogramming with additional EEA and miR-302/367 targeting
• Day 15 scRNA-seq reveals reprogramming progression
• CRISPRa cells are reprogrammed with higher fidelity than transgenic cells

## Graphical abstract

**Figure:**
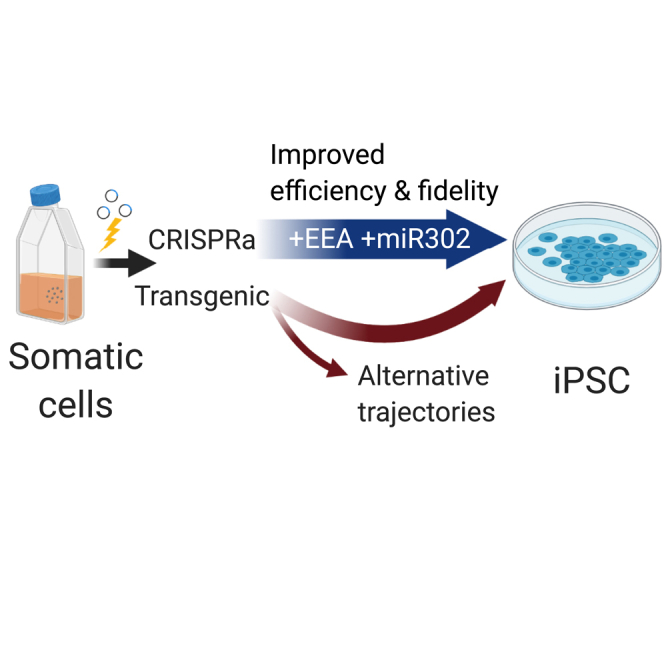

## Teaser

Forced expression of transcription factors can lead to off-target gene activation and heterogeneous reprogramming, resulting in aberrant iPSCs. Activation of endogenous pluripotency factors by CRISPRa reduces this heterogeneity. Efficient reprogramming was dependent on the additional targeting of the EEA motif and the miR-302/367 locus, with single-cell analysis revealing that these cells reprogrammed more specifically compared to the conventional reprogramming method.

## Introduction

CRISPR activation (CRISPRa) uses a catalytically inactivated form of Cas9 (dCas9) fused with a transactivator domain that enables the activation of transcription from endogenous promoters (Bikard et al., 2013). Advantages of CRISPRa over conventional reprogramming (Fusaki et al., 2009; Okita et al., 2011; Warren et al., 2010) include the direct transcriptional activation from endogenous loci, high multiplexing capability, and the potential to target non-coding regulatory elements. We have recently shown reprogramming of human fibroblasts by CRISPRa targeting the promoters of *OCT4* (*POU5F1*), *SOX2*, *KLF4*, *MYC*, and *LIN28A* and an Alu-motif enriched near promoter regions of genes expressed during embryo genome activation (EEA motif [embryo genome activation-enriched Alu-motif]) (Weltner et al., 2018). However, low reprogramming efficiency hampers the use of CRISPRa. Several small molecules and genes, including pluripotent stem cell micro RNAs (miRNA), can improve reprogramming efficiency (Subramanyam et al., 2011). The miRNA cluster miR-302/367 is expressed at high levels in human embryonic stem cells (hESCs) (Houbaviy et al., 2003) and is known to be sufficient to reprogram somatic cells to pluripotency (Anokye-Danso et al., 2011; Miyoshi et al., 2011). Therefore, the targeted activation of miR-302/367 expression with CRISPRa could improve CRISPRa reprogramming efficiency.

As CRISPRa mediated reprogramming is a novel method for inducing pluripotency, its reprogramming trajectories are as yet unknown. Recent advances in single-cell RNA sequencing (scRNA-seq) technology have facilitated our understanding of reprogramming processes and revealed the importance of the early embryonic programs for successful reprogramming (Cacchiarelli et al., 2015; Francesconi et al., 2019; Liu et al., 2020; Schiebinger et al., 2019; Tran et al., 2019). However, low efficiency and high background of non-reprogrammed cells can obscure the transcriptional analysis of fibroblast CRISPRa reprogramming. This problem could be overcome by using human lymphoblastoid cell lines (LCLs), which are generated by Epstein-Barr virus transformation of B lymphocytes (Neitzel, 1986). *In vitro* LCL cultures grow in suspension, while emerging stem cell colonies attach to the culture surface, providing a simple means for the specific enrichment of the cells undergoing reprogramming. Furthermore, vast collections of LCLs are stored in biobank repositories, providing a virtually unlimited source of reprogramming material due to the immortal nature of these cells (Sie et al., 2009).

In this study, we showed that simultaneous targeting of the EEA motif and the promoter of the miR-302/367 cluster enhanced the reprogramming efficiency of fibroblasts and LCL cells and accelerated the kinetics of induced pluripotent stem cell (iPSC) formation. Using scRNA-seq analysis, we profiled conventional and three combinations of CRISPRa reprogramming across different time points. We found that the cells reprogrammed using the CRISPRa progress to the pluripotent state with high fidelity showing a uniform expression of pluripotency genes and minimal heterogeneity. This is in contrast to the conventional reprogramming, which leads to a longer reprogramming route, often resulting in alternative cell types. These results support the use of CRISPRa for improving the quality of human pluripotent reprogramming.

## Results

### CRISPRa reprogramming of LCL

**Figure 1:**
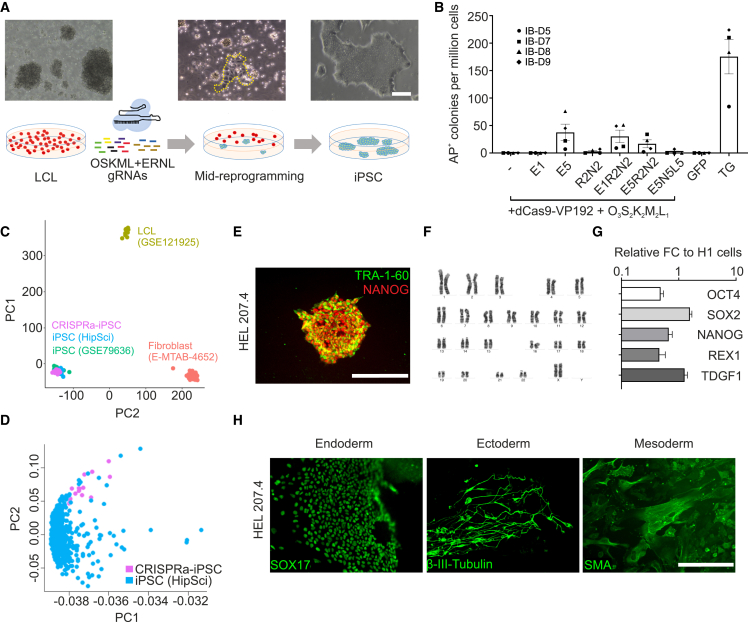
CRISPRa reprogramming of LCL (A) Schematic representation of the LCL reprogramming protocol with bright-field images from different reprogramming stages. Attached reprogramming intermediates at day 10 are encircled with yellow ticks. Scale bar, 400 μm. (B) Reprogramming efficiency of LCL from 4 different donors (IB-D5, IB-D7, IB-D8, and IB-D9). The most efficient CRISPRa condition, E5, averaged 38 colonies, while the conventional transgenic (TG) method averaged 176 colonies. Reprogramming conditions are indicated on the x axis. n = 4; each point represents an independent assay from each of the 4 patient-derived LCLs. Error bars, SEMs. E, EEA, L, LIN28A, N, NANOG, R, REX1; numbers refer to the number of guides. GFP-only containing plasmid was used as a negative reprogramming control. (C) Validation of CRISPRa iPSC (n = 15 individual cell lines), with PCA showing the bulk RNA-seq data grouping with reference iPSC lines (HipSci, GEO: GSE79636) and away from LCL (GEO: GSE121925) and fibroblast (E-MTAB-4652) cell lines. Samples are listed in Table S2. (D) Further validation with PCA by comparing CRISPRa iPSC (n = 15 individual cell lines) and HipSci iPSC lines (n = 661 individual cell lines). (E) Immunocytochemistry of undifferentiated hESC markers NANOG and TRA-1-60 in CRISPRa iPSC line HEL207.4. Scale bar, 200 μm. See also Figure S1B. Antibodies and primers used in the article are listed in Table S3. (F) Normal karyotype of iPSC line HEL207.4. See also Figure S1D. (G) Expression of selected hESC markers from 5 different CRISPRa-iPSC lines compared to the H1 hESC. n = 5; each replicate is from individual iPSC lines; error bars, SEMs. (H) Multilineage differentiation of HEL207.4 cells shown by immunostaining for endodermal (SOX17), ectodermal (β-III-tubulin) and mesodermal (smooth muscle actin, SMA) germ layer derivatives. Scale bar, 200 μm. See also Figure S1E.

We began by validating the CRISPRa system for the reprogramming of LCL. The attachment of reprogramming intermediates to the culture plates was observed by reprogramming day 10, after which unattached cells could be removed (Figure 1A). To test for different factor combinations, CRISPRa targeting *NANOG*, *REX1* (*ZFP42*), *LIN28A*, and EEA motif in addition to the basal reprogramming factor guides for *OCT4*, *SOX2*, *KLF4*, *MYC*, and *LIN28A* that were efficient in fibroblast reprogramming (Weltner et al., 2018) were used. The highest reprogramming efficiency was observed with additional EEA motif targeting by using a combination of five guides (hereafter referred to as CRISPRa + E) (Figures 1B and S1A). We opted to use multiple EEA motif targeting guides for the rest of the work, over *NANOG* and *REX1* targeting, to track reprogramming progress by endogenous *NANOG* activation with immunocytochemistry.

We then confirmed the pluripotency of generated CRISPRa iPSC lines. Principal-component analysis (PCA) of bulk RNA-seq of 15 CRISPRa iPSC lines from 5 LCL and 2 fibroblast donors demonstrated that all CRISPRa iPSC lines grouped with previously published iPSC lines generated from blood and fibroblast cells using conventional methods (Carcamo-Orive et al., 2017; Kilpinen et al., 2017) and distinct from LCL and fibroblasts (Kaisers et al., 2017; Ozgyin et al., 2019) (Figure 1C). CRISPRa iPSC lines were then compared more closely with a total of 661 Human Induced Pluripotent Stem Cells Initiative (HipSci) iPSC lines, which showed that our cell lines grouped closely together with the HipSci lines (Kilpinen et al., 2017) (Figure 1D). Further characterization showed a normal karyotype, expression of undifferentiated hESC markers, differentiation into three embryonic germ layer derivatives, and the loss of episomal reprogramming plasmids (Figures 1E–1H and S1B–S1E), supporting the notion that the LCL had been reprogrammed into *bona fide* iPSC.

### miR-302/367 promoter targeting improves CRISPRa reprogramming efficiency

**Figure 2:**
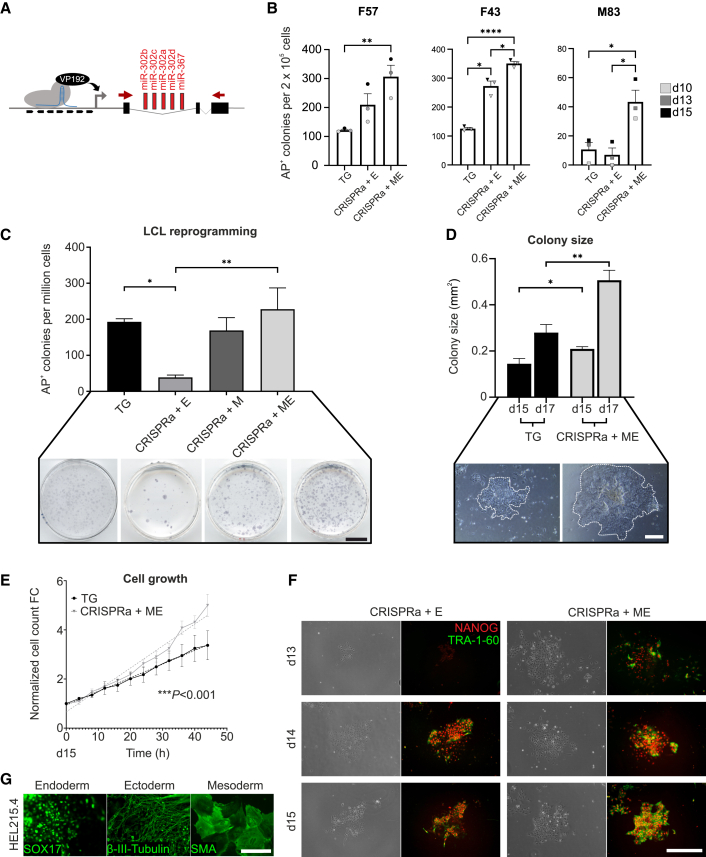
miR-302/367 promoter targeting improves CRISPRa reprogramming efficiency (A) Schematic representation of the CRISPRa activation of miR-302/367 cluster home gene (MIR302CHG). Red arrows show the locations of the qPCR primers. Guides targeting the MIR302CHG are listed in Table S4. (B) Reprogramming efficiency of primary fibroblasts using 3 separate donor lines, F57, F43, and M83, on days 10, 13, and 15. Reprogramming conditions are indicated on the x axis. n = 3; each point represents an independent assay in which cells were counted on either day 10, 13, or 15, as indicated by the legend; error bars, SEMs; p values were calculated with repeated measures 1-way ANOVA post-hoc Tukey’s test, ^∗^p < 0.05, ^∗∗^p < 0.01, ^∗∗∗∗^p < 0.0001. (C) Reprogramming efficiency of LCL. Reprogramming conditions are indicated on the x axis. n = 6 independent experiments; error bars, SEMs; p values were calculated with 1-way ANOVA post-hoc Tukey’s test, ^∗^p < 0.05, ^∗∗^p < 0.01. Representative AP-stained culture plates from each reprogramming condition are shown below. Scale bar, 1 cm. (D) Measurement of average iPSC-like colony size at days 15 and 17. n = 6 independent experiments; error bars, SEMs; p value was calculated with Student’s t test, ^∗^p < 0.05, ^∗∗^p < 0.01. Bright-field images of iPSC-like colonies in TG and CRISPRa + ME conditions at day 17 are shown below. Colony edges have been highlighted with white tick marks. Scale bar, 400 μm. (E) Growth rate of the iPSC-like cells in TG and CRISPRa + ME conditions from day 15 onward. Cell counts are normalized to the day 15 starting point. n = 6 independent experiments; error bars, SEMs. Significance of the difference between the linear regression lines (R^2^ TG = 0.4965, CRISPRa + ME = 0.8475) was calculated using GraphPad Prism’s linear regression analysis equivalent to ANCOVA, ^∗∗∗^p < 0.001. See also Figure S2G. (F) Immunocytochemistry images showing the hESC markers NANOG and TRA-1-60 in CRISPRa + EEA and CRISPRa + ME reprogramming at days 13–15. Scale bar, 400 μm. (G) Multilineage differentiation of CRISPRa + ME reprogrammed HEL215.4 iPSC line shown by immunostaining for endodermal (SOX17), ectodermal (β-III-tubulin), and mesodermal (smooth muscle actin, SMA) germ layer derivatives. Scale bar, 200 μm. See also Figure S2H.

To improve the CRISPRa reprogramming efficiency, we targeted the promoter of the miR-302/367 cluster home gene *MIR302CHG* (Figures 2A and S2A). Multiple primary fibroblast lines were transfected with the miR-302/367 targeting guides on top of CRISPRa + E (hereafter referred to as CRISPRa + ME) along with the transgenic (TG) and CRISPRa + E conditions. CRISPRa + ME significantly increased the reprogramming efficiency and colony size when compared to other reprogramming conditions (Figures 2B and S2B–S2D). CRISPRa + ME was the only reprogramming condition that properly induced iPSC colonies from an 83-year-old male-derived primary fibroblast line M83 known for being difficult to reprogram (Trokovic et al., 2015) (Figures 2B, S2C, and S2D). LCL reprogramming with the dCas9 activator plasmid and miR-302/367 guides alone did not yield any colonies (Figure S2E), but the targeting of miR-302/367 cluster on top of basal CRISPRa (hereafter referred to as CRISPRa + M) increased LCL reprogramming efficiency 6-fold (mean 169 alkaline phosphatase-positive [AP^+^] colonies, n = 6 independent experiments) compared to CRISPRa + E. Reprogramming efficiency further increased up to 8-fold (mean 228 AP^+^ colonies, n = 6 independent experiments) with the combined CRISPRa + ME condition (Figure 2C).

To define the time point for analyzing the reprogramming with scRNA-seq, we characterized the attachment and growth of the reprogramming intermediates. As attached reprogramming intermediates could be seen on day 10, the cells that were still in suspension at this stage were re-plated. After 1 week none of the re-plated cells had started to form iPSC-like colonies, suggesting that most of the reprogramming cells had already attached by day 10 (Figure S2F). Live cell imaging of the reprogramming cell cultures from days 15–17 revealed significantly larger colony sizes in CRISPRa + ME compared to the TG condition (Figure 2D) and increased cell numbers (Figures 2E and S2G). Staining for hESC markers NANOG and TRA-1-60 showed NANOG^+^ and TRA-1-60^+^ colonies emerging by day 14 in the CRISPRa + E condition, while the CRISPRa + ME condition presented NANOG^+^ and TRA-1-60^+^ colonies already at day 13 (Figure 2F). This suggested accelerated reprogramming kinetics of the miR-302/367 CRISPRa-targeted cells and that day 15 would be an optimal time point to explore the transcriptomic profiles of reprogramming intermediates by scRNA-seq analysis. Finally, to verify that the CRISPRa + ME reprogramming in LCL was robust in producing pluripotent cells, and not due to a single donor effect, four additional LCL donor lines were reprogrammed using CRISPRa + ME. All of the donor lines differentiated properly into all three embryonic germ layer derivatives, confirming the pluripotent nature of the cells (Figures 2G and S2H).

### scRNA-seq captures the progression of CRISPRa reprogramming

**Figure 3:**
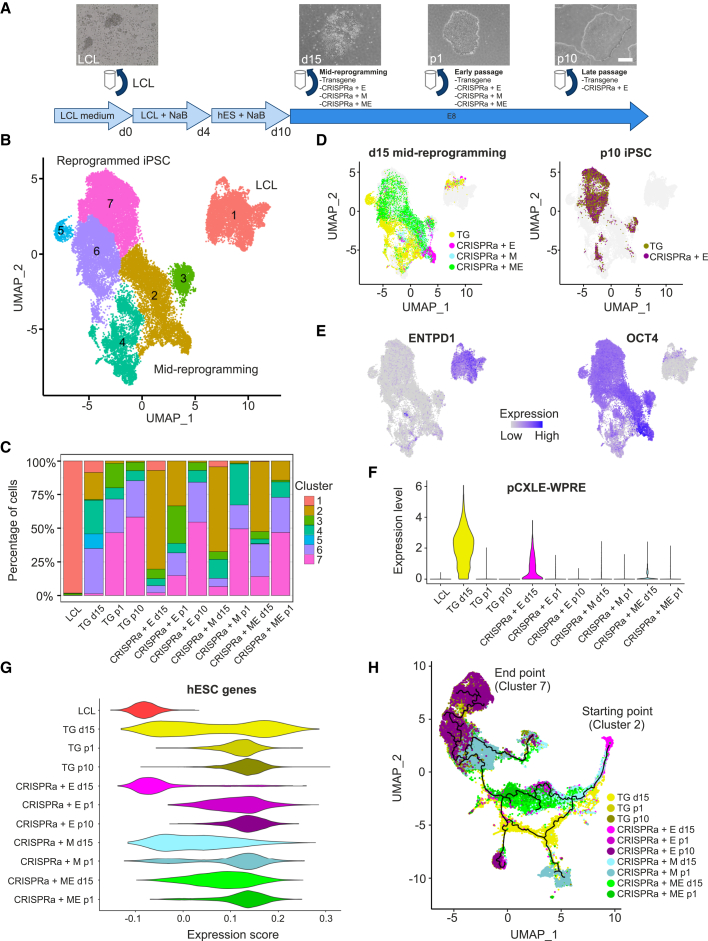
scRNA-seq captures the progression of CRISPRa reprogramming (A) Schematic representation of the time-resolved scRNA-seq sample collection strategy. (B) UMAP plot representing the 7 clusters across 32,758 cells from different reprogramming conditions and time points. (C) Cell composition across the 7 clusters in each sample. Colors indicate each cluster as seen in (B). Individual cluster cell counts are listed in Table S5. (D) UMAPs showing the distributions of mid-reprogramming and passage 10 cells. Gray dots represent cells from other samples. (E) Expression of LCL marker ENTPD1 and hESC marker OCT4. Colors indicate expression levels (blue, high; gray, low). (F) Violin plot showing the expression of the episomal vector (short plasmid backbone sequence pCXLE-WPRE) across the different reprogramming conditions and time points. (G) Expression score of 140 selected hESC markers across the different reprogramming conditions and time points. (H) Pseudotime analysis showing the trajectory of the cells during reprogramming, with cluster 1 excluded. Time points and reprogramming conditions are indicated in the key. See also Figure S4A.

To investigate changes in transcription at the single-cell level, we prepared samples for scRNA-seq at various time points of the reprogramming process. To mitigate the effects of genetic background, cells were collected from the same donor reprogrammed using the TG and three different CRISPRa (CRISPRa + E, + M, + ME) conditions at reprogramming days 0 (starting LCL) and 15 (mid-reprogramming cells), as well as from passage 1 and 10 iPSCs (Figure 3A). Characterization of the cell populations that arose during reprogramming was performed by unsupervised clustering analysis, which identified seven cell clusters (Figure 3B; Table S5). LCL confined into cluster 1, separate from the other clusters. Notably, while the mid-reprogramming cells from 3 CRISPRa conditions were located mostly in cluster 2, the TG mid-reprogramming cells clustered separately between clusters 4, 5, and 6. Passage 1 and 10 iPSCs were found in clusters 6 and 7, with over half of the passage 10 iPSC localizing in cluster 7, marking it as the endpoint of the reprogramming process (Figures 3C and 3D). Interestingly, 14% of mid-reprogramming CRISPRa + ME cells were in iPSC cluster 7, indicating that these cells may proceed toward the iPSC state faster than the other conditions (Figures 3C and 3D).

We then integrated the scRNA-seq to bulk RNA-seq data of published LCL (Ozgyin et al., 2019) and iPSC (Carcamo-Orive et al., 2017; Kilpinen et al., 2017) datasets and used PCA to visualize the relationship between clusters (Figure S3A). As expected, cluster 1 grouped close to the reference LCL, cluster 7 grouped closest to the reference iPSC samples, while the mid-reprogramming clusters grouped between the LCL and iPSC (Figure S3A). The cell identity of cluster 1 was further confirmed using the expression of well-known LCL markers *ENTPD1*, *FCER2*, *CD70*, and *LFA3* (Rajesh et al., 2011) (Figures 3E and S3B). These markers were downregulated in mid-reprogramming and iPSC clusters, consistent with previous reports (Rajesh et al., 2011). The known reprogramming and hESC markers *L1TD1*, *OCT4*, *NANOG*, *SOX2*, *TDGF1*, and *REX1* were detected in the mid-reprogramming and iPSC clusters and were almost completely absent from the LCL cluster, as expected (Figures 3E and S3B).

However, a notable exception was that these hESC markers were mostly absent in TG mid-reprogramming cluster 4 (Figures 3E and S3B). The TG mid-reprogramming cells with high hESC marker expression were instead located in iPSC cluster 6 as well as in a small cluster, 5, consisting almost solely of mid-reprogramming TG cells (Figures 3C–3E). To detect the presence of episomal vectors, we used reads mapped to the transcribed elements in the plasmid backbone (WPRE). Retention of the high expression of episomal vectors in the transgene mid-reprogramming was observed while they were mostly absent from the CRISPRa conditions (Figure 3F). To further distinguish the pluripotency signature between samples, gene expression of 140 (Table S6) well-known hESC markers were analyzed (Figure 3G). In particular, TG mid-reprogramming displayed a bimodal expression of hESC markers when compared to CRISPRa conditions (Figure 3G). This indicated the presence of a heterogeneous mixture of pluripotent-like and non-pluripotent cells in the TG reprogramming conditions. Trajectory analysis on cells excluding LCL cluster 1 revealed that cells in the TG condition took an alternative trajectory early on, leading to multiple endpoints, while the CRISPRa + ME condition followed a more direct route to the iPSC state (Figures 3H and S4A). Further analysis of the TG mid-reprogramming cells showed the loss of transgene expression along with pseudotime progression and a bifurcation of the cells into *OCT4*^+^ (clusters 5–7) and *OCT4*^−^ (cluster 4) populations (Figures S4C and S4D), suggesting that loss of transgene expression before endogenous pluripotency factor activation may hinder proper reprogramming progression. These results support the notion that CRISPRa + ME progress to the iPSC state with improved fidelity and kinetics, while TG cells are more dependent on their episomal vectors and thus take a longer reprogramming route, resulting in alternative endpoints.

### miR-302/367 and EEA motif act synergistically to promote pluripotency at the mid-reprogramming stage

**Figure 4:**
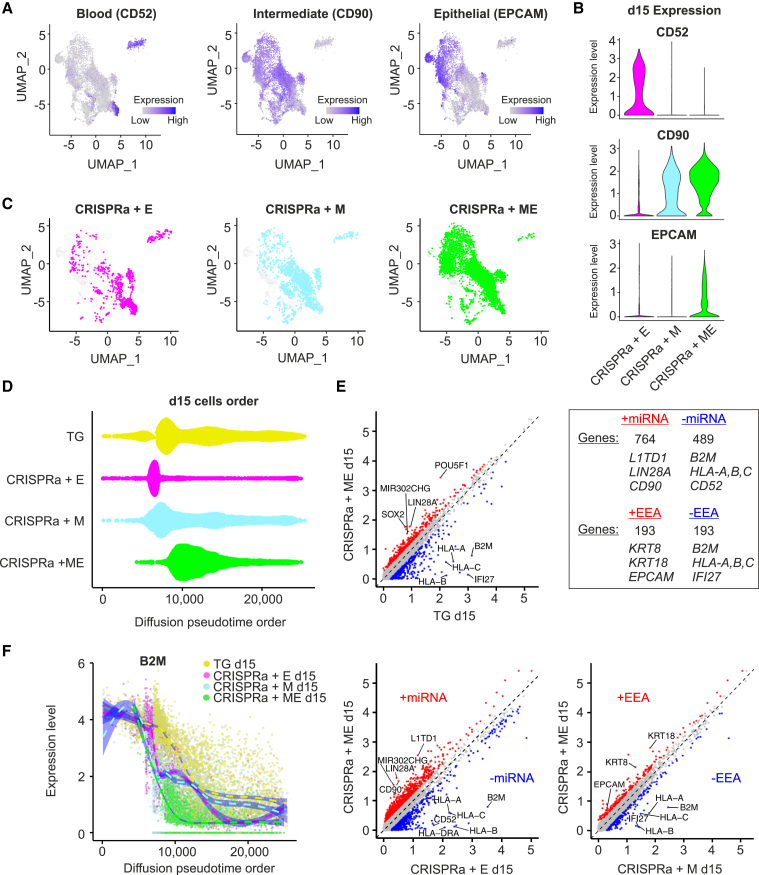
miR-302/367 and EEA motif act synergistically to promote pluripotency at the mid-reprogramming stage (A) Expression of blood cell marker CD52, intermediate reprogramming marker CD90, and epithelial marker EPCAM in all mid-reprogramming cells. Colors indicate expression levels (blue, high; gray, low). (B) Violin plots showing the expression levels of CD52, CD90, and EPCAM across the different CRISPRa reprogramming conditions. See also Figure S4H. (C) UMAPs showing the distribution of mid-reprogramming CRISPRa + E, CRISPRa + M, and CRISPRa + ME cells. Gray dots represent cells from other mid-reprogramming cell samples. (D) Mid-reprogramming cells ordered by diffusion pseudotime. See also Figure S4B. (E) Comparison of expression profiles between mid-reprogramming CRISPRa + ME and TG cells (top left), CRISPRa + ME and CRISPRa + E (bottom left), and CRISPRa + ME and CRISPRa + M (bottom right). Log-scaled average expression levels of all cells in each condition are plotted. At top right, the number of significantly highly expressed genes in CRISPRa conditions with or without miR-302/367 or EEA targeting are shown, with some of the top hits listed. These genes are marked as red or blue in the plots. (F) Diffusion pseudotime combined with the expression level of B2M in the mid-reprogramming samples. Dashed lines and blue shades are fitted generalized additive model, with 95% confidence interval in each condition. Each color represents the different reprogramming condition.

We then characterized the day 15 mid-reprogramming samples in more detail. Cluster 2 contained a subpopulation of cells with a high expression of the lymphocyte marker *CD52* (Hale et al., 1990), which consisted primarily of CRISPRa + E cells (Figures 4A–4C). Cluster 2 cells located closer to the iPSC clusters showed expression of the primed hESC surface marker *CD90* (*THY1*) (Collier et al., 2017), but still lacked the expression of another marker, *EPCAM*, which is involved in the epithelialization process (Huang et al., 2011). This suggests that these cells were intermediate reprogramming cells (Figures 4A, 4B, and S4H). By clustering only the day 15 sample cells, distinct clusters for naive and primed cells could be detected (Figures S4F and S4I).

The mid-reprogramming samples appeared to cluster in different parts of the UMAP plot with different CRISPRa reprogramming conditions (Figures 4C and S4G). Therefore, the progression to the iPSC state was estimated by applying a diffusion pseudotime analysis on all of the samples, including LCL and iPSC (Figure S4B). Among the mid-reprogramming samples, CRISPRa + ME progressed to the iPSC state faster compared to the other conditions, while most of the CRISPRa + E remained stuck at the beginning of reprogramming (Figures 4D and S4B). These results suggest that the endogenous activation of miR-302/367 helps to induce the cells out of the initial blood cell-like state.

To evaluate the gene expression changes between different reprogramming conditions at the mid-reprogramming stage, the gene expression profile of CRISPRa + ME was compared with the other three conditions (Figure 4E). Higher expression of hESC genes such as *LIN2*8A and *L1TD1* and the surface marker *CD90* was observed in the CRISPRa + ME compared with CRISPRa + E. The miR-302/367 cluster targeting influenced the expression of approximately three times more genes compared to the EEA targeting, including multiple pluripotency-associated genes (Figure 4E). Thus, the miR-302/367 targeting may help lower the barrier for the activation of pluripotency-associated genes.

We further investigated the rate of blood cells reprogramming using the expression of genes characteristic for blood cells, such as major histocompatibility complex (MHC) class I human leukocyte antigens (*HLA-A*, *HLA-B*, and *HLA-C*) and an associated gene *B2M*. These MHC class I-associated genes are expressed in almost all nucleated cells (Gussow et al., 1987; Ploegh et al., 1981), but their expression is notably higher in the blood cells (Boegel et al., 2018; Mabbott et al., 2013; Papatheodorou et al., 2020; Thul et al., 2017). The expression of these genes was reduced in the CRISPRa + ME compared to all other mid-reprogramming conditions (Figure 4E). Furthermore, when comparing the expression levels of *B2M* and *HLA-A* to the diffusion map pseudotime, their expression was rapidly decreased in the CRISPRa + ME cells (Figures 4F and S4E). These findings support the notion that the CRISPRa + ME cells lose their initial blood cell-like identity and progress faster toward the iPSC state.

The expression of epithelial cell specific genes *KRT8*, *KRT18* (Fuchs and Weber, 1994), and *EPCAM* were increased in the CRISPRa + ME condition compared to the CRISPRa + M (Figure 4E), suggesting that the EEA motif targeting may aid in epithelialization during reprogramming, in addition to its reported role in aiding in pluripotency factor activation (Weltner et al., 2018). This effect may be of importance, especially in the later stages of reprogramming, as seen by the *EPCAM* expression between CRISPRa + ME and CRISPRa + M (Figures 4A–4C). In summary, our results suggest that the miR-302/367 and EEA motif targeting act synergistically by enhancing the progression of reprogramming from the initial blood cell state and aiding epithelialization.

### CRISPRa + ME cells progress to the pluripotent state with improved fidelity

**Figure 5:**
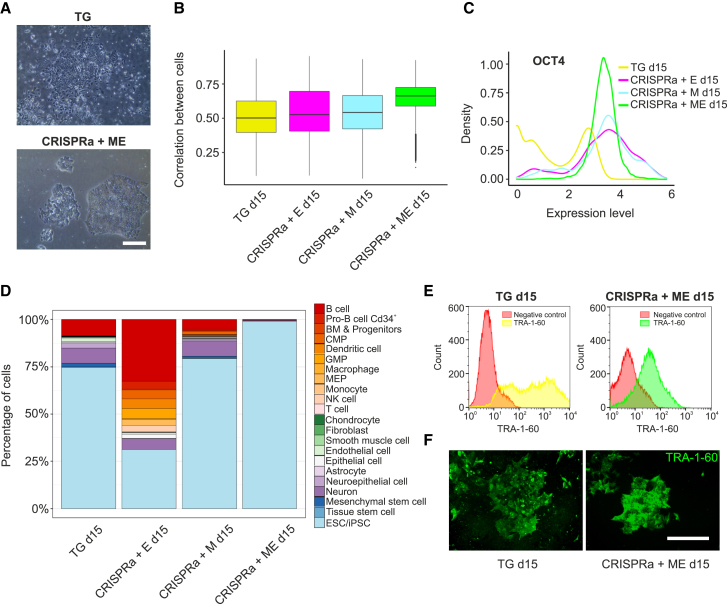
CRISPRa + ME cells progress to the pluripotent state with improved fidelity (A) Morphology of colonies during TG and CRISPRa + ME mid-reprogramming. Scale bar, 400 μm. (B) Correlation coefficients of gene expression profile among cells in all mid-reprogramming conditions. (C) Distribution of OCT4 expression level in cells from all mid-reprogramming conditions. (D) Cell identity annotation in all mid-reprogramming conditions using SingleR. BM, bone marrow; CMP, common myeloid progenitors; GMP, granulocyte monocyte progenitors; MEP, megakaryocyte-erythroid progenitor cell. (E) Flow cytometry analysis of hESC marker TRA-1-60 in TG and CRISPRa + ME mid-reprogramming cells. Red, negative control. (F) Immunocytochemistry images showing TRA-1-60 expression in TG and CRISPRa + ME mid-reprogramming cells. Scale bar, 400 μm.

To assess the mid-reprogramming cell heterogeneity observed by microscopy (Figure 5A), we calculated the correlation coefficients of highly variable genes between all cells for each mid-reprogramming condition. CRISPRa + ME had a higher correlation coefficient compared to all of the other conditions, suggesting less heterogeneity among the cells (Figure 5B). The distribution of *OCT4* expression level demonstrated a sharp single peak in CRISPRa + ME, indicating that most of these cells showed similar expression levels of *OCT4* (Figure 5C), whereas other conditions showed broader or bimodal peaks, indicating varying expression levels. To identify the cell populations at the mid-reprogramming stage, cells were annotated against the bulk RNA-seq data of the Human Primary Cell Atlas (Mabbott et al., 2013) using SingleR (Aran et al., 2019) (Figure 5D). A total of 99% of CRISPRa + ME cells showed the highest transcriptional similarity to hESC or iPSC, while CRISPRa + E showed the highest heterogeneity, with ∼60% of cells being annotated as blood cells. TG and CRISPRa + M cells had similar heterogeneity profiles, with ∼10% of cells annotated as blood cells and another 10% as neuronal cells, indicating differentiation toward alternative cell types (Figure 5D). These results reinforced our findings that the CRISPRa + ME condition reprogrammed the cells toward the iPSC state with greatly improved fidelity.

Finally, to validate our findings from scRNA-seq, we analyzed the mid-reprogramming cells with flow cytometry for the hESC surface marker TRA-1-60 (Pera et al., 2000). The results showed that CRISPRa + ME cells had more uniform TRA-1-60 expression on day 15 when compared to the TG condition (Figures 5E and 5F), supporting the bimodal expression pattern of TG cells. These results demonstrate the use of CRISPRa as an efficient reprogramming tool able to reduce cellular heterogeneity in human iPSC induction.

## Discussion

Based on our results, activation of endogenous genes by CRISPRa can be used for efficient reprogramming of fibroblasts and LCL into iPSC. The efficiency was dependent on the additional targeting of the *MIR302CHG* transcript and the EEA motif. Further scRNA-seq analysis of day 15 reprogramming intermediates revealed less heterogeneity in the CRISPRa + ME reprogrammed cells compared to other conditions.

Numerous repositories contain LCL generated from a variety of patients, and conventional reprogramming methods have been used to derive iPSC from them (Barrett et al., 2014; Fujimori et al., 2016; Rajesh et al., 2011; Thomas et al., 2015). The CRISPRa reprogramming method described herein broadens the available tools for using these repositories for disease modeling. Transcriptionally, the CRISPRa iPSCs were indistinguishable from other high-quality iPSC lines generated from the large repositories using conventional TG transcription factor-mediated reprogramming methods (Carcamo-Orive et al., 2017; Kilpinen et al., 2017).

A key aspect of the efficient reprogramming of LCL with CRISPRa was targeting the *MIR302CHG* locus. Although the ectopic overexpression of the miR-302/367 has been reported to reprogram both mouse and human cells into iPSC (Anokye-Danso et al., 2011; Miyoshi et al., 2011), we did not observe any iPSC colonies when only miR-302/367 was targeted. This is possibly due to the modest activation of *MIR302CHG* transcript by CRISPRa compared to the ectopic expression of miRNA used in previous studies. The activation of the *MIR302CHG* transcript promoted the transition of LCL toward iPSC-like cells. Our observations are consistent with the reported role of miR-302/367 in reducing the expression of a number of repressive factors in reprogramming (Subramanyam et al., 2011) and enhancing reprogramming (Kogut et al., 2018). In addition, the enhanced activation of *LIN28A* by miR-302/367 targeting may contribute to a more efficient expression of pluripotency factors by regulating the synthesis of Let-7 miRNA (Ustianenko et al., 2018; Worringer et al., 2014). Activation of additional pluripotency-associated miRNAs may prove to be an efficient way of improving CRISPRa reprogramming. In line with this, a recent article demonstrated that the transcriptional activation of another miRNA cluster on chromosome 19 (C19MC) accelerates human cellular reprogramming (Mong et al., 2020).

Previous studies have described detailed roadmaps of somatic cell reprogramming toward pluripotency (Cacchiarelli et al., 2015; Hussein et al., 2014; Liu et al., 2020; Schiebinger et al., 2019; Takahashi et al., 2014; Takahashi and Yamanaka, 2016; Wang et al., 2018). These roadmaps have pinpointed the heterogeneity of the reprogramming process and the transient cell populations and off-target cell types emerging during the process. Our day 15 reprogramming samples replicate the heterogeneity and reprogramming progression described previously, spanning from somatic-like cells to iPSC-like cells. Importantly, this mid-reprogramming sample time point enabled us to assess the effect of different reprogramming methods on the reprogramming of human LCL.

The best reprogramming outcome was observed by concurrent targeting of both miR-302/367 and EEA motif. The reprogramming effect of the EEA motif targeting has previously been linked to its contribution to the activation of *NANOG* and *REX1* (Weltner et al., 2018). The scRNA-seq data from the LCL suggest that the EEA motif targeting additionally promotes epithelialization, which is supported by the increased *KRT8/18* expression and higher proportion of *EPCAM*^+^ cells at the later time point clusters (Fuchs and Weber, 1994; Huang et al., 2011). *KRT18* expression appears to be directly activated by CRISPRa targeting of the EEA motif, similar to what we have observed previously in fibroblasts (Weltner et al., 2018). Intriguingly, this also suggests a role for the Alu-KRT18 axis in controlling human early embryo development, as KRT8 and KRT18 were recently described as the first fate determinants that drive early embryo lineage specification (Lim et al., 2020), and the EEA motif was originally detected from embryo-sequencing data (Töhönen et al., 2015). However, further studies are still required to decipher the exact mechanism behind the role of miRNA and EEA targeting on improving the reprogramming efficiency.

Importantly, the optimized CRISPRa reprogramming, with *MIR302CHG* and EEA motif targeting, seems to proceed more homogeneously than conventional reprogramming. Analysis of the cells being reprogrammed revealed differences, particularly in the expression of the endogenous reprogramming factors. The barrier for the activation of endogenous pluripotency factors may thus contribute to the divergent route that TG reprogramming cells seem to take to pluripotency. However, high levels of TG *OCT4* expression may be one additional explanation for the increased heterogeneity of the TG samples. A recent study reported that ectopic *OCT4* expression is detrimental to the generation of high-quality mouse iPSCs, due to the activation of off-target genes (Velychko et al., 2019). Thus, the activation of endogenous factors may result in improved iPSC quality through a more deterministic reprogramming process. This also supports the use of CRISPRa for improving the specificity of pluripotent reprogramming. Alternatively, targeting of additional factors specific to the alternative cell-type clusters could promote more specific derivation of other reprogrammed cell types from the reprogramming intermediates using CRISPRa (e.g., induced primitive endoderm or trophoblast cells).

In conclusion, the optimized CRISPRa approach reprograms cells toward pluripotency efficiently and with improved fidelity. These findings support the use of CRISPRa to improve the quality of human pluripotent reprogramming.

## Experimental procedures

Additional methods and more in-detail descriptions of bulk and scRNA-seq can be found in the supplemental experimental procedures.

### Ethical consent

The generation of the human iPSC lines was approved by the Coordinating Ethics Committee of the Helsinki and Uusimaa Hospital District (no. 423/13/03/00/08) with informed consent of the donors. LCLs were obtained from THL Biobank (Finnish Institute of Health and Welfare, www.thl.fi/biobank), and the experiments were performed according to the contract and in compliance with the general terms of the THL Biobank (application no. BB2016_56, amendment BB2018_33).

### Cell culture

LCL were cultured in LCL medium (RPMI 1640 with GlutaMAX [Thermo Fisher Scientific]) supplemented with 15% fetal bovine serum (FBS) (Life Technologies) and 1 μM sodium pyruvate. Human embryonic kidney cells (HEK293, ATCC [American Type Culture Collection] line CRL-1573) and human fibroblasts were cultured in fibroblast medium (Dulbecco’s Modified Eagle Medium [DMEM, Sigma-Aldrich, D6546]) supplemented with 10% FBS, 2 mM GlutaMAX (GIBCO), and 100 μL/mL penicillin-streptomycin (Sigma-Aldrich). iPSCs were cultured in plates coated with Matrigel (Corning) in E8 (GIBCO) or E8 Flex (GIBCO) medium. The medium was changed every other day. All of the cells were kept in an incubator at 37°C and 5% CO_2_ and were routinely tested for mycoplasma contamination.

### CRISPRa and conventional reprogramming of LCL

LCL were passaged the day before reprogramming. On the day of reprogramming (day 0), LCLs were dissociated into single cells by trituration and washed in PBS (Thermo Fisher Scientific). Cells were electroporated with the Neon transfection system (Invitrogen) using R buffer. A total of 10^6^ cells and 6 μg plasmid mixture, containing 1.5 μg dCas9-activator plasmid (Addgene #69535) and 4.5 μg guide plasmids, were electroporated in a 100-μL tip using 1,300 V, 10 ms, and 3× pulse conditions. For conventional reprogramming, a plasmid mixture containing 2 μg of each ectopic expression plasmid (Addgene #27077, #27078, #27080) (Okita et al., 2011) was transfected with the same electroporation conditions as CRISPRa. Electroporated LCLs were then plated onto cell culture dishes in LCL growth medium supplemented with 0.25 mM sodium butyrate (NaB). The GFP expression from the episomal dCas9 activator-plasmid or from the ectopic expression plasmids was visualized the next day to verify a successful electroporation. After 3 days, the cells were passaged onto Matrigel-coated plates. On day 4 after the transfections, the medium was changed to hES medium (DMEM/F12 with GlutaMAX [Life Technologies]), supplemented with 20% KnockOut Serum Replacement (Life Technologies), 0.0915 mM 2-mercaptoethanol (Life Technologies), 1× Non-Essential Amino Acids (Life Technologies), and 6 ng/mL basic fibroblast growth factor (bFGF) (PeproTech) supplemented with 0.25 mM NaB. The cell growth medium was changed to E8 medium at day 10 of reprogramming, and the unattached LCLs were removed at this point. The cells were cultured until day 21, when the colonies were large enough to be manually picked and plated on Matrigel-coated wells in E8 medium. The media were changed every other day. The list of plasmids used for LCL reprogramming is provided in Table S1.

### CRISPRa and conventional reprogramming of fibroblasts

Fibroblasts (human foreskin fibroblasts [HFFs, ATCC line CRL-2429] and 43- to 83-year-old donor-derived primary fibroblast lines F72, F57, F43, and M83]) were seeded 4 days before the start of reprogramming. On the day of reprogramming (day 0), cells were detached as single cells from the culture plates with TrypLE Select (GIBCO) and washed with PBS. Cells were electroporated using the Neon transfection system (Invitrogen) and reprogrammed by CRISPRa, as described previously (Weltner et al., 2018; Weltner and Trokovic, 2021). Conventional reprogramming was performed with the same transfection conditions as CRISPRa, using the three ectopic expression plasmids mentioned previously (Addgene #27077, #27078, and #27080) (Okita et al., 2011). Electroporated fibroblasts were plated on Matrigel-coated plates immediately after the transfections. The medium was changed every other day, and on day 4, the fibroblast medium was changed to a 50:50 ratio of fibroblast medium and hES medium, supplemented with 0.25 mM NaB. The cells were kept growing with medium changes every other day, until the formed iPSC colonies were either AP stained or collected for further analysis on days 10, 13, and 15.

### Bulk RNA-seq and processing

Total RNA was extracted from 15 iPSC lines (Table S2), and purified using the NucleoSpin RNA Plus kit (Macherey-Nagel). Bulk RNA-seq was performed as a service at Novagen after the cells passed quality control. The expression profiles of the RNA-seq data were compared to published reference datasets of iPSC, LCL, and fibroblasts (GEO: GSE79636, HipSci, GEO: GSE121926, E-MTAB-4652) (Carcamo-Orive et al., 2017; Kaisers et al., 2017; Kilpinen et al., 2017; Ozgyin et al., 2019) analyzed with the same methods.

### Single-cell RNA-seq and processing

Cells were dissociated with Accutase at 37°C for 5 min, resuspended in PBS + 0.04% BSA on ice, and passed through a Flowmi tip strainer (Fisher Scientific) to yield a single-cell suspension. The quality of the samples was assessed using a Luna cell counter (Logos Biosystems). scRNA-seq was performed using the 10X Genomics Chromium Single Cell 3′RNA-seq platform at the Institute of Molecular Medicine Finland (FIMM).

### Statistical analysis

Statistical analyses were performed with Student’s t test, analysis of variance (ANOVA), and the linear regression slope comparison test (GraphPad Prism version 8.4.2) equivalent to the analysis of covariance (ANCOVA), as described in the figure legends. p < 0.05 was considered significant (^∗^p < 0.05, ^∗∗^p < 0.01, ^∗∗∗^p < 0.001, ^∗∗∗∗^p < 0.0001).

### Data and code availability

The accession number for the RNA-seq data reported in this paper isGEO: GSE162530. The rest of the data are available in the main text or in the supplemental information.

## Author contributions

R.T., J.W., and D.B. were responsible for the conceptualization. J.W. and D.B. were responsible for the methodology. J.S., L.L., C.S., and A.W. were responsible for the investigation. M.Y., J. Kvist, and H.K. were responsible for the bioinformatics analysis. J.S., M.Y., and J. Kvist were responsible for visualization. T.O. and A.K. were responsible for the resources. J.S. was responsible for writing the original draft. All of the authors were involved in reviewing and editing. R.T., J.W., E.-M.J., S.K., J. Kere, and T.O. were responsible for supervision. R.T., J. Kere, and T.O. were responsible for funding acquisition.

## Conflicts of interest

Based on the article’s results, the University of Helsinki has filed a patent application on the improved CRISPRa + ME reprogramming method to the National Board of Patents and Registration of Finland (patent application no. 20215848).

## Supplemental information

Document S1. Supplemental experimental procedures, Figures S1–S4, and Tables S1–S6

Document S2. Article plus supplemental information

## Footnotes

Supplemental information can be found online at https://doi.org/10.1016/j.stemcr.2021.12.017.
