# Supplementary material for: CRISPR activation enables high-fidelity reprogramming into human pluripotent stem cells
Source: Stem Cell Reports. 2022 Jan 20;17(2):413–26. doi: 10.1016/j.stemcr.2021.12.017 (PMC8828555; doi:10.1016/j.stemcr.2021.12.017)
Supplement: Document S1. Supplemental experimental procedures, Figures S1–S4, and Tables S1–S6 [file mmc1.pdf]

Stem Cell Reports, Volume *17*

Supplemental Information

CRISPR activation enables high-fidelity reprogramming into human pluripotent stem cells

Joonas Sokka, Masahito Yoshihara, Jouni Kvist, Laura Laiho, Andrew Warren, Christian Stadelmann, Eeva-Mari Jouhilahti, Helena Kilpinen, Diego Balboa, Shintaro Katayama, Aija Kyttälä, Juha Kere, Timo Otonkoski, Jere Weltner, and Ras Trokovic

Supplemental information

Supplemental Figures

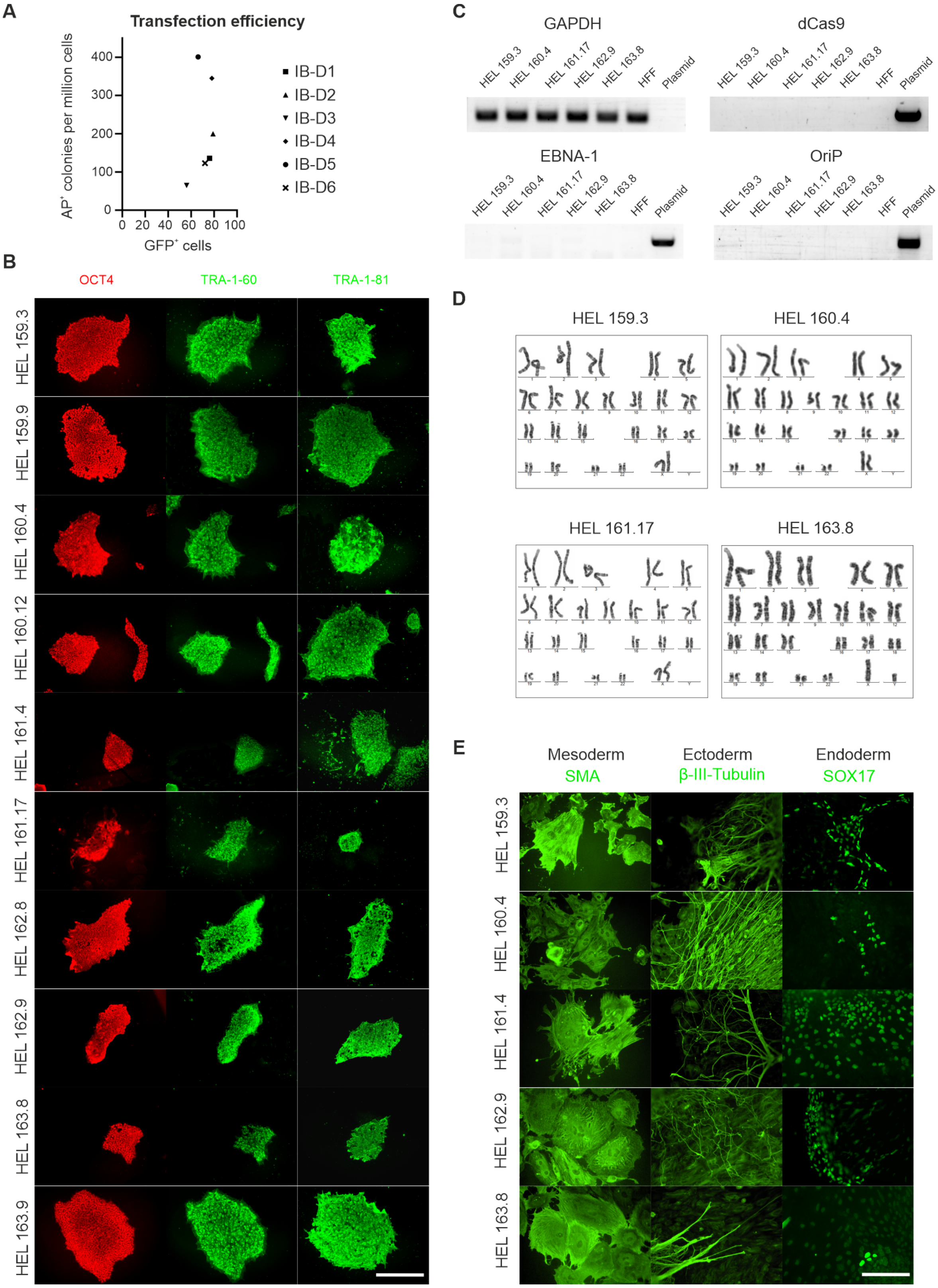

Figure S1: Validation of CRISPRa-iPSC lines, related to Figure 1

A) Transfection efficiency of six different LCL reprogrammed with the conventional transgenic method in relation to the amount of generated colonies.

B) Immunocytochemistry images showing OCT4, TRA-1-60 and TRA-1-81 stainings in generated CRISPRa-iPSC lines. Scale bar= 400 µm

C) Genomic DNA PCR gel images showing the removal of episomal plasmids from generated CRISPRa-iPSC lines. GAPDH= gDNA control, HFF= fibroblast control, Plasmid= dCas9-activator plasmid control

D) Karyotyping results of iPSC lines generated from LCL donors IB-D1, IB-D2, IB-5 and IB-D6

E) EB-assay with stainings for endodermal (SOX17), ectodermal (β-III-tubulin) and mesodermal (Smooth muscle actin, SMA) germ lineage derivatives. Scale bar= 200 µm

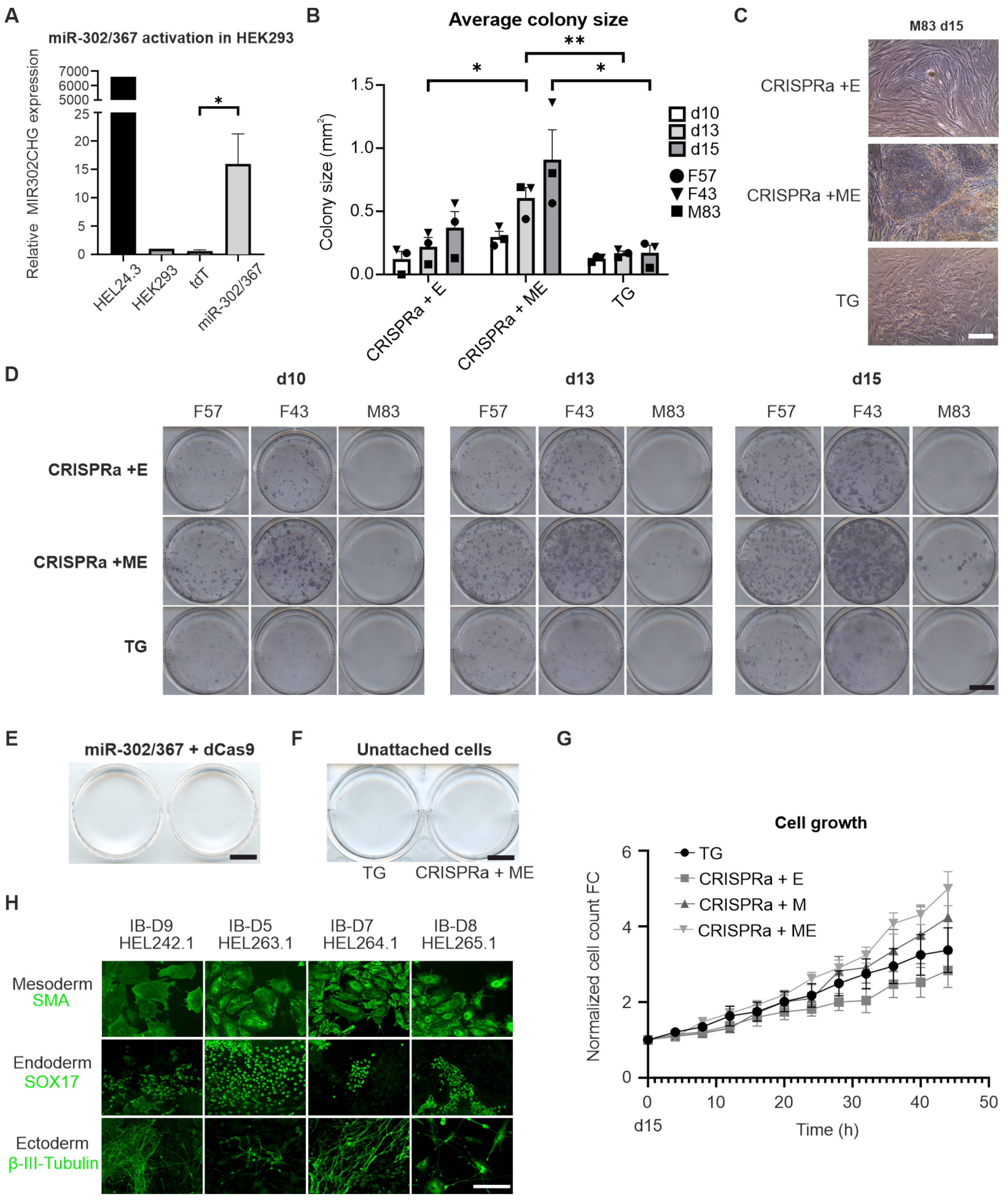

A
miR-302/367 activation in HEK293
Relative MIR302CHG expression
7000
6000
5000
25
20
15
10
5
0
*
HEL24.3
HEK293
tdT
miR-302/367
B
Average colony size
Colony size (mm²)
1.5
1.0
0.5
0.0
*
**
*
d10
d13
d15
F57
F43
M83
CRISPRa + E
CRISPRa + ME
TG
C
M83 d15
CRISPRa +E
CRISPRa +ME
TG
D
d10
d13
d15
F57
F43
M83
CRISPRa +E
CRISPRa +ME
TG
E
miR-302/367 + dCas9
F
Unattached cells
TG
CRISPRa + ME
G
Cell growth
Normalized cell count FC
6
4
2
0
TG
CRISPRa + E
CRISPRa + M
CRISPRa + ME
0
10
20
30
40
50
d15
Time (h)
H
IB-D9
HEL242.1
IB-D5
HEL263.1
IB-D7
HEL264.1
IB-D8
HEL265.1
Mesoderm
SMA
Endoderm
SOX17
Ectoderm
β-III-Tubulin

Figure S2: Additional induction data, related to Figure 2

A) Validation of CRISPR activation of miR-302/367 cluster in HEK293 cells with and without miR-302/367 gRNA targeting. n=5 independent experiments, Error bars SEM, Student's t-test *=p<0.05. TdTomato guide was used as a negative control, and iPSC line (HEL24.3) was used as a positive control.

B) Measurement of iPSC-like colony size at days 10, 13 and 15 using three different primary fibroblast lines and reprogramming methods. n=3, each point represents an independent assay where cells were counted on either day 10, 13 or 15 as indicated by the legend, Error bars=SEM, p-values were calculated with repeated measures one-way ANOVA, *=p<0.05, **=p<0.01

C) Bright field images of M83 iPSC colonies on reprogramming day 15 using three different reprogramming conditions. Scale bar= 400 µm

D) AP-stainings of primary fibroblast lines F57, F43 and M83 on reprogramming days 10, 13 and 15 using three different reprogramming methods. Scale bar= 1 cm

E) AP-staining of LCL reprogrammed with dCas9 activator and miR-302/367 targeting guides only. Scale bar= 1 cm

F) AP-staining of re-plated unattached day 10 cells from TG and CRISPRa + ME inductions. Scale bar= 1 cm

G) iPSC-like cells growth in TG, CRISPRa + E, CRISPRa + M and CRISPRa + ME conditions from reprogramming day 15 to day 17. Cell count fold change is normalized to the day 15 starting point. n=6 independent experiments, Error bars=SEM

H) Multilineage differentiation of four different LCL donor lines reprogrammed by CRISPRa + ME shown by immunostaining for endodermal (SOX17), ectodermal (β-III-tubulin) and mesodermal (Smooth muscle actin, SMA) germ layer derivatives. Scale bar= 200 µm

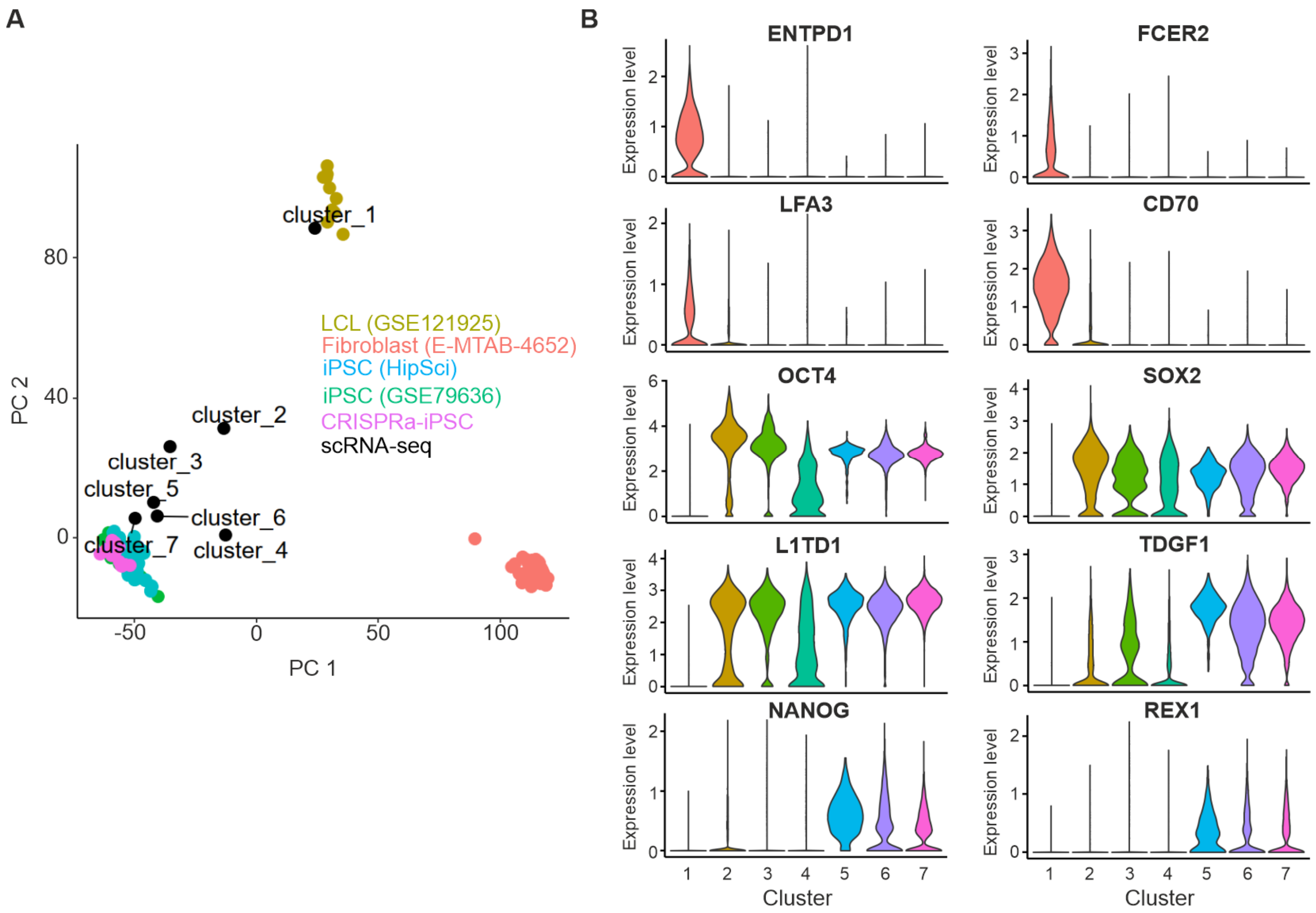

Figure S3: scRNA-seq gene expression, related to Figure 3
A) PCA of combined bulk RNA-seq data and scRNA-seq clusters with reference iPSC (HipSci, GSE79636), LCL (GSE121925) and fibroblast (E-MTAB-4652) cell lines.
B) Violin plots showing expression levels of selected LCL (ENTPD1, FCER2, LFA3, CD70) and undifferentiated hESC (OCT4, SOX2, L1TD1, TDGF1, NANOG, REX1) associated markers across the clusters.

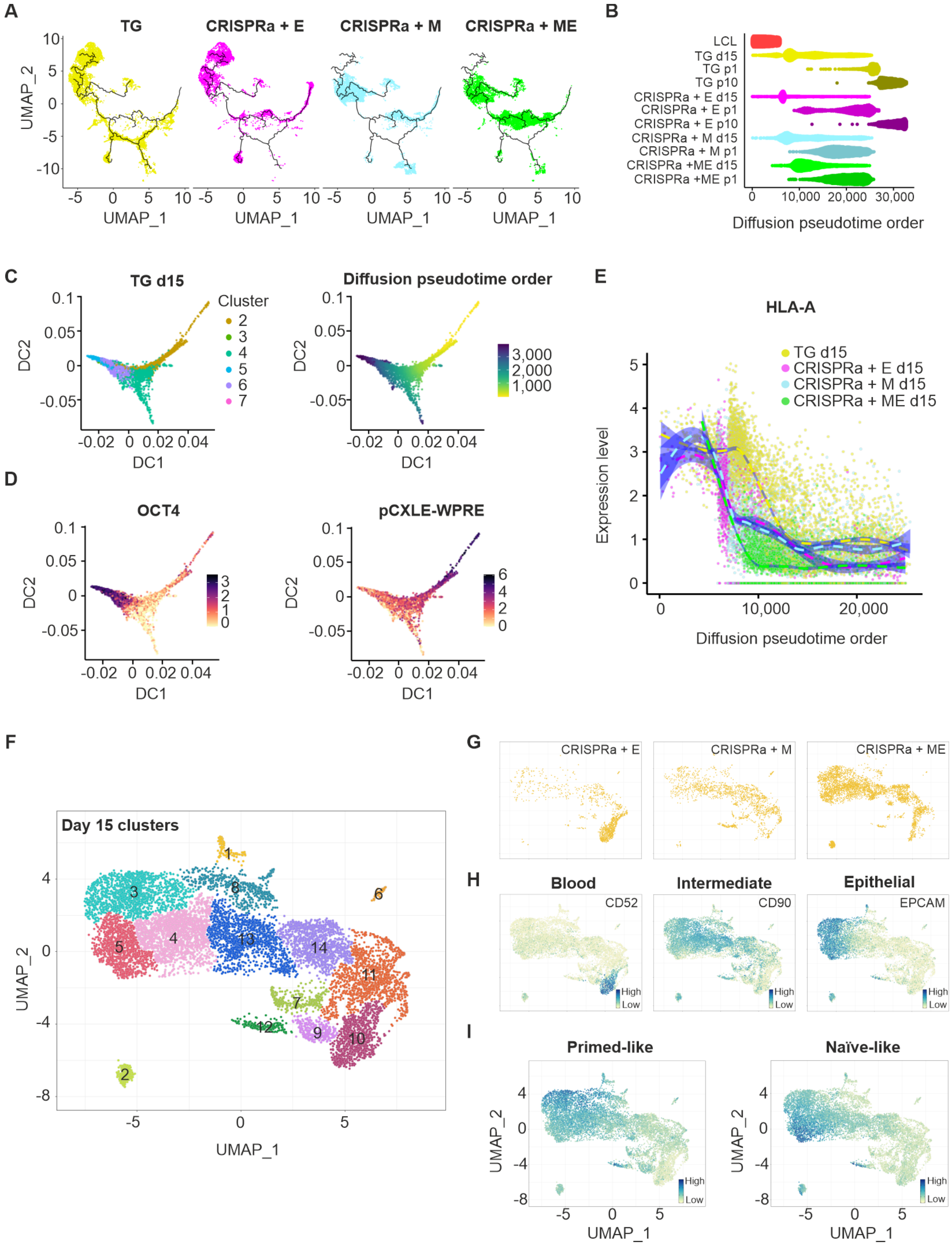
A
TG
CRISPRa + E
CRISPRa + M
CRISPRa + ME
UMAP_2
UMAP_1
B
LCL
TG d15
TG p1
TG p10
CRISPRa + E d15
CRISPRa + E p1
CRISPRa + E p10
CRISPRa + M d15
CRISPRa + M p1
CRISPRa +ME d15
CRISPRa +ME p1
Diffusion pseudotime order
C
TG d15
Cluster
Diffusion pseudotime order
DC2
DC1
D
OCT4
pCXLE-WPRE
E
HLA-A
TG d15
CRISPRa + E d15
CRISPRa + M d15
CRISPRa + ME d15
Expression level
Diffusion pseudotime order
F
Day 15 clusters
G
CRISPRa + E
CRISPRa + M
CRISPRa + ME
H
Blood
Intermediate
Epithelial
CD52
CD90
EPCAM
High
Low
I
Primed-like
Naïve-like

Figure S4: Additional pseudotime and diffusion map analyses, related to Figures 3 and 4
A) Pseudotime analysis showing the trajectory of the cells from each reprogramming condition during reprogramming with cluster 1 excluded.
B) Diffusion pseudotime analysis showing the predicted order of the cells from all samples based on their expression profiles
C) Diffusion component analysis of mid-reprogramming TG cells excluding LCL cluster 1.
D) Expression of OCT4 and pCXLE-WPRE on the diffusion map.
E) Diffusion pseudotime combined with the expression level of HLA-A in the mid-reprogramming samples. Dashed lines and blue shades are fitted generalized additive model with 95% confidence interval in each condition. Each color represents the different reprogramming condition.
F) UMAP clustering of day 15 samples only
G) Distribution of different CRISPRa condition cells in d15 only clusters
H) Expression of blood cell marker CD52, intermediate reprogramming marker CD90, and epithelial marker EPCAM in d15 only clusters
I) Expression of primed and naïve markers in d15 only clusters. Genes are listed in supplemental table S6.

Supplemental Tables

Table S1: Reprogramming plasmids, related to Figures 1 and 2

Plasmid	Method	Addgene number
pCXLE-hOCT3/4-shp53-F	Conventional	27077
pCXLE-hSK	Conventional	27078
pCXLE-hUL	Conventional	27080
pCXLE-dCas9VP192-T2A-EGFP-shP53	CRISPRa	69535
GG-EBNA-O3S2K2M2L1-PP	CRISPRa	102902
GG-EBNA-EEA-guide1-PGK-Puro	CRISPRa	102904
GG-EBNA-EEA-5guides-PGK-Puro	CRISPRa	102898
GG-EBNA-R2N2-PGK-Puro	CRISPRa	-
GG-EBNA-R2N2-EEAg1-PGK-Puro	CRISPRa	-
GG-EBNA-R2N2-EEAg5-PGK-Puro	CRISPRa	-
GG-EBNA-N5L5-EEAg1-PGK-Puro	CRISPRa	-
GG-EBNA-MIR302g7-PGK-Puro	CRISPRa	-
pCXLE-EGFP	Control	27082

Table S2: Bulk RNA-seq samples, related to Figure 1

iPSC line	Cell origin	Donor line	Sex	Reprogramming method
HEL159.3	LCL	IB-D6	F	CRISPRa
HEL159.9	LCL	IB-D6	F	CRISPRa
HEL160.4	LCL	IB-D2	F	CRISPRa
HEL160.12	LCL	IB-D2	F	CRISPRa
HEL161.4	LCL	IB-D1	F	CRISPRa
HEL161.17	LCL	IB-D1	F	CRISPRa
HEL162.8	LCL	IB-D4	F	CRISPRa
HEL162.9	LCL	IB-D4	F	CRISPRa
HEL163.8	LCL	IB-D5	M	CRISPRa
HEL163.9	LCL	IB-D5	M	CRISPRa
HEL139.2	Fibroblast	F72	F	CRISPRa
HEL139.5	Fibroblast	F72	F	CRISPRa
HEL139.8	Fibroblast	F72	F	CRISPRa
HEL140.1	Fibroblast	HFF	M	CRISPRa
HEL141	Fibroblast	HFF	M	CRISPRa

Table S3: Primers and antibodies, Related to Figures 1, 2 and 5

Gene	Forward primer	Reverse primer	Product length (bp)
OriP	TTC CAC GAG GGT AGT GAA CC	TCG GGG GTG TTA GAG ACA AC	544
EBNA-1	ATC GTC AAA GCT GCA CAC AG	CCC AGG AGT CCC AGT AGT CA	666
dCas9	AAA CAG CAG ATT CGC CTG GA	CTG TCT GCA CCT CGG TCT TT	1934
GAPDH	AAG AAG GTG GTG AAG CAG GC	CAG GAA ATG AGC TTG ACA AAG	164
OCT4 (POU5F1)	TTG GGC TCG AGA AGG ATG TG	GTG AAG TGA GGG CTC CCA TA	193
SOX2	GCC CTG CAG TAC AAC TCC AT	TGC CCT GCT GCG AGT AGG A	85
NANOG	CTC AGC CTC CAG CAG ATG C	TAG ATT TCA TTC TCT GGT TCT GG	94
REX1 (ZFP42)	CGT TTC GTG TGT CCC TTT CAA	CCT CTT GTT CAT TCT TGT TCG T	106
LIN28A	AGG AGA CAG GTG CTA CAA CTG	TCT TGG GCT GGG GTG GCA G	74
TDGF1	TCA GAG ATG ACA GCA TTT GGC	TTC AGG CAG CAG GTT CTG TTT A	118
KLF4	CCG CTC CAT TAC CAA G	CAC GAT CGT CTT CCC CTC TT	80
cMYC	AGC GAC TCT GAG GAG GAA CA	CTC TGA CCT TTT GCC AGG AG	87
pri-miR-302/367 (MIR302CHG) intronic region	TAA CTT TAT TGT ATT GAC CGC AGC TC	GTC ACA GCA AGT GCC TCC AT	106
pri-miR-302/367 (MIR302CHG) exonic region	TGG AGG AGA ACA CGA ATC TTTG GG	ACA AGC AGC AAA AGC AAT TGA GGT A	127/74 splice variants
Cyclophilin G	CAA TGG CCA ACA GAG GGA AG	CCA AAA ACA ACA TGA TGC CCA	94
**Primary Antibody**	**Manufacturer**	**Host Species and Dilution**	**Catalog**
OCT3/4	Santa Cruz Biotechnology	Rabbit 1:500	SC-9081
NANOG	Cell signaling technologies	Rabbit 1:200	4903S
TRA-1-60	Invitrogen	Mouse 1:500	MA1-023

TRA-1-81	Invitrogen	Mouse 1:250	MA1-024
SOX17	R&D Systems	Goat 1:500	AF1924
α-Smooth muscle actin	Sigma	Mouse 1:500	A2547
β-III-tubulin	Abcam	Rabbit 1:500	Ab18207
**Secondary Antibody**	**Manufacturer**	**Host Species and Dilution**	**Catalog**
Anti-Rabbit IgG (H+L) Alexa Fluor 594 (Red)	Invitrogen	Donkey 1:500	A21207
Anti-Rabbit IgG (H+L) Alexa Fluor 488 (Green)	Invitrogen	Donkey 1:500	A21206
Anti-Mouse IgG (H+L) Alexa Fluor 594 (Red)	Invitrogen	Donkey 1:500	A21203
Anti-Mouse IgG (H+L) Alexa Fluor 488 (Green)	Invitrogen	Donkey 1:500	A21202
Anti-Goat IgG (H+L) Alexa Fluor 594 (Red)	Invitrogen	Donkey 1:500	A11058
Anti-Goat IgG (H+L) Alexa Fluor 488 (Green)	Invitrogen	Donkey 1:500	A11055

Table S4: Guide RNA oligos, related to Figure 2

Gene	Guide	Sequence
miR-302/367	1	GTGGAAAGGACGAAACACCgAAGAATAGTATAAATAGAAGgttttagagctaGAAAtag
miR-302/367	2	GTGGAAAGGACGAAACACCgATCTCAGAGAATCATTACAAgttttagagctaGAAAtag
miR-302/367	3	GTGGAAAGGACGAAACACCgAGGGAATGTATGATCCTGGGgttttagagctaGAAAtag
miR-302/367	4	GTGGAAAGGACGAAACACCgAAAAGGATCCAGACCCACCCgttttagagctaGAAAtag
miR-302/367	5	GTGGAAAGGACGAAACACCgTTTAAGAGGAAGATATCTTGgttttagagctaGAAAtag
miR-302/367	6	GTGGAAAGGACGAAACACCgATGCCATCAAACAAGCAGATgttttagagctaGAAAtag
miR-302/367	7	GTGGAAAGGACGAAACACCgCAATGCCTTTCTCGGCTCAGgttttagagctaGAAAtag

Table S5: scRNA-seq cluster cell counts, related to Figure 3

Cluster	LCL	TG d15	TG p1	TG p10	CRISPRa + E d15	CRISPRa + E p1	CRISPRa + E p10	CRISPRa + M d15	CRISPRa + M p1	CRISPRa + ME d15	CRISPRa + ME p1
**1**	5315	371	0	0	92	0	0	75	1	19	1
**2**	15	852	32	30	966	300	31	1109	65	2052	368
**3**	67	36	324	200	93	250	185	98	30	221	33
**4**	18	1073	152	244	69	63	264	249	1373	138	302
**5**	0	465	0	0	0	0	0	0	0	4	0
**6**	0	1438	448	860	71	150	905	108	800	956	679
**7**	0	63	834	1869	26	133	1665	116	2220	557	1215

Table S6: hESC, primed and naïve associated gene sets, related to Figures 3 and 4

**hESC**	ABCG2, ALPP, ANO6, BCL3, BNIP3, BRIX1, BUB1, CCNA2, CD24, CD59, CD9, CD90, CDC42, CDH1, CDK1, CDK8, CHD1, CHD7, COMMD3, CRABP2, CTNNB1, CXCL5, DIAPH2, DNMT1, DNMT3B, DPPA2, DPPA3, DPPA4, DPPA5, EDNRB, EPCAM, ESRG, ESRRB, FBXO15, FGF2, FGF4, FGF5, FOXA2, FOXD3, FUT4, FZD1, GABRB3, GAL, GATA6, GBX2, GDF3, GJB1, GJB4, GJC1, GRB7, HDAC1, HES1, HESX1, HHEX, HMGA2, HOXB5, HSPA9, IDO1, IFITM2, IGF2BP2, IL6ST, ITGA4, ITGA6, ITGB1, KAT5, KCNIP3, KHDC3L, KIT, KITLG, KLF4, KLF5, KNL1, L1TD1, LCK, LEF1, LEFTY1, LEFTY2, LIFR, LIN28A, LMNA, MYBL2, MYC, NACC1, NANOG, NODAL, NOG, NR5A2, NR6A1, NUMB, OCT4, OTX2, PCGF2, PECAM1, PITX2, PIWIL1, PIWIL2, PIWIL4, PML, PODXL, PRDM14, PRDM5, PROM1, PTEN, PUM2, REST, REX1, RIF1, SALL4, SCN1A, SEMA3A, SFRP2, SMAD1, SMAD2, SMAD3, SMAD4, SMAD5, SMAD9, SOX15, SOX2, STAT3, SUMO2, TAF8, TBX3, TCF3, TCL1A, TDGF1, TERF1, TERT, TEX19, TFCP2L1, THAP11, TRIM22, TRIM28, TRIM6, UTF1, XIST, ZFX, ZIC1, ZSCAN10, ZSCAN4
**Primed**	NLGN4X, PTPRG, AC022140.1, LARGE1, JARID2, RIMS2, KCND2, NRXN1, TCF7L1, DLGAP1, TMEM132B, FOXO1, ADCY2, CADM2, GALNT17, TERF1, ANK3, FIRRE, AP002856.2, AC009446.1, SHISA9, AL590705.1, MGAT4C, AL365259.1, PTPRZ1, RYR2, LINC00678, DTNA, GPC6, ANOS1, PCDH11X, GPC4, LEFTY1
**Naïve**	CHODL, NLRP7, SLC16A10, UTF1, MT1G, AC011447.3, ZYG11A, CBFA2T2, MT1H, ZNF600, AKAP12, TRIML2, WDHD1, PRODH, RESF1, SERPINB9, NLRP2, NLRP1, ASRGL1, AC092546.1, LINC01950, PTCHD1, SLC25A16, DNMT3L, ZNF729, CNR2, PBX4, BRDT

Supplemental experimental procedures

Cell imaging

For light microscopy a Leica DM IL LED microscope (Leica-microsystems) was used, and pictures of live cells were taken with an attached Leica EC3 (Leica-microsystems) camera using LAS-EZ imaging software (Leica-microsystems). EVOS FL cell imaging system (Thermo Fisher) was used for fluorescence imaging. Whole culture plate images for the cell growth assays were taken with the IncuCyte S3 live-cell analysis system (Sartorius).

Guide RNA design and validation

Guide cassettes were designed and produced as previously described (Balboa et al., 2015; Weltner et al., 2018). Guide RNA (gRNA) expression cassettes, containing the U6 promoter, chimeric single gRNA and Pol III terminator, were assembled by PCR amplification and concatenated into GG-dest plasmid (Addgene #69538) using Golden Gate assembly. Concatenated guide sets were cloned from GG-dest into an episomal OriP-EBNA1 containing plasmid backbone for reprogramming experiments. A list of miR-302/367 targeting gRNA oligonucleotides is provided in supplemental table S4. Guides targeting pluripotency factors and the EEA-motif (Addgene #102902, #102904 and #102898) have been described previously (Weltner et al., 2018). To validate gRNAs, HEK293s were transfected using 4:1 ratio of FuGENE HD transfection reagent (Promega) with 500 ng of dCas9 transactivator encoding plasmid (Addgene plasmid #69535) and 100-200 ng of gRNA cassette. Transfected cells were cultured for 72 h, after which they were collected for quantitative reverse transcription PCR (qRT-PCR) analysis.

Alkaline phosphatase staining

iPSC colonies were fixed with 4% PFA for 10 min and washed with PBS. Cells were then stained with NBT/BCIP (Roche) containing buffer (0.1 M Tris HCL pH 9.5, 0.1M NaCl, 0.05 M $MgCl2$) until purple precipitate formed. The reaction was stopped by washing the plates with PBS.

Embryoid body assay

For the embryoid body (EB) assay iPSC were passaged on ultra-low attachment 6-well culture plates in hES medium without bFGF. The medium was supplemented with 10 µM ROCKi Y-27632 for the first 24 h. The cells were cultured as EB aggregates for two weeks and the medium was changed every other day. After two weeks, the EBs were plated onto a 24 well-plate and cultured for 7 days in hES medium without bFGF. Thereafter, the EBs were fixed with 4% PFA for 30 min for immunocytochemistry.

Quantitative reverse transcription PCR

Total RNA was extracted from cells using NucleoSpin RNA Plus kit (Macherey-Nagel) and complementary DNA (cDNA) was synthesized from 1 µg of RNA by reverse transcription with 0.5 µl Moloney murine leukemia virus reverse transcriptase (Promega), 0.5 µl Riboblock RNAse inhibitor (Thermo Scientific), 0.2 µl Random hexamers (Promega), 1 µl Oligo (dT) 18 Primer (Thermo Scientific) and 2.5 ul dNTP (2.5 mM) at 37 °C for 90 minutes. For qRT-PCR reactions, 1 µl of cDNA was added to a master mix containing 4 µl of 5x HOT FIREPol EvaGreen qPCR Mix Plus (no ROX) (Solis Biodyne) and 10 µl of DEPC-H_2O per reaction. QIAgility (Qiagen) liquid handling system was used for pipetting 15 µl of sample mix with 5 µl of 2 µM forward and reverse primer mix per reaction with all reactions made as duplicates. Samples were amplified in Rotor-Gene Q (Qiagen) with a thermal cycle of 95 °C for 15 min, followed by 35 cycles of 95 °C 25 s, 57 °C 25 s, 72 °C 25 s, followed by a melting step. The qRT-PCR results were analyzed by the ΔΔCt method, using Cyclophilin G as a housekeeping gene. The expression data were presented as a fold change compared to the control. For the reverse transcription PCR (RT-PCR) reaction, 1 µl of synthesized cDNA was used per reaction and added to the master mix containing DreamTaq DNA polymerase (Thermo Scientific). Primers used in RT-PCR and qRT-PCR assays are listed in supplemental table S3.

Immunocytochemistry

The cells were plated onto 24-well culture plates prior to the immunostainings. Cells were fixed with 4% paraformaldehyde (PFA) (Fisher Chemical) in PBS for 15 min. The cells were then permeabilized by 0.5% Triton X-100 in PBS for 10 min and treated with Ultra Vision (UV)-blocker (Thermo Scientific) for 10 min. Primary

antibodies were diluted in 0.1% Tween in PBS, added to the wells and incubated for 24 h in dark at 4 °C on a Stuart SSL4 see-saw rocker. Secondary antibodies, and Hoechst 33342 (Thermo Fisher Scientific) to stain the nuclei, were diluted in 0.1% Tween in PBS and added to the wells. The wells were then incubated in the dark at RT for 30 min on the see-saw rocker. Primary and secondary antibodies used are listed in supplemental table S3.

Flow cytometry

Prior to the flow cytometry analysis, the cells were detached as single cells from the culture plates with TrypLE Select. 1 x 10^6 cells were resuspended in flow cytometry-buffer (5% FBS in PBS) containing 1:10 TRA-1-60 primary antibody and incubated in the dark at RT for 20 min on the see-saw rocker. After aspirating the primary antibody solution, the secondary antibody was diluted 1:100 in flow cytometry-buffer and added to the cells. Cells were incubated in the dark at RT for 20 min on the see-saw rocker, and resuspended in 500 µl flow cytometry-buffer and transferred to 5 ml Falcon flow cytometry-tubes. Cells were analyzed in FACSCalibur (Becton Dickinson) using 3 x 10^4 cells per sample. The acquired data were analyzed and visualized using the FlowJo v.10.7 software.

Karyotyping

2 x 10^6 cells were suspended in medium supplemented with 0.1 µg/mL KaryoMAX Colcemid Solution in PBS and incubated for 4 h at 37 °C. Cells were resuspended in 0.075 M KCl and incubated at 37 °C for 10 min. Fixative (3:1 ratio of methanol and acetic acid) was added dropwise to the cell suspension. Fixation was repeated three times before storing the samples at 4 °C until shipping. Karyotyping was performed as a service by Ambar in Barcelona, Spain.

Bulk RNA sequencing and processing

Total RNA was extracted from 15 iPSC lines (Supplemental table 2) and purified by NucleoSpin RNA Plus kit (Macherey-Nagel). Bulk RNA-seq was performed as a service at Novagen after the cells passed quality control. Samples were sequenced using Illumina PE150. The raw data was filtered with cutadapt (Martin, 2011) to remove adapter sequences, ambiguous (N) and low quality bases (Phred score < 25). The filtered reads were mapped to a customized version of the human reference genome (GRCh38 release 99) with STAR aligner (Dobin et al., 2013). The backbone of the plasmid sequences (pCXLE-gw) and gene annotations to the reference genome were added as a separate chromosome. Gene expression was counted from read pairs mapping to exons using featureCount in Rsubreads(Liao et al., 2019). Duplicates, chimeric and multi mapping reads were excluded, as well as reads with low mapping scores (MAPQ < 10). The read count data were analyzed with DESeq2 (Love et al., 2014). The expression profiles of the RNA-seq data were compared to published reference datasets of iPSC, LCL and fibroblasts (GSE79636, HipSci, GSE121926, E-MTAB-4652) (Carcamo-Orive et al., 2017; Kaisers et al., 2017; Kilpinen et al., 2017; Ozgyin et al., 2019) analyzed with the same methods.

Single-cell RNA sequencing and processing

Cells were dissociated with Accutase at 37 °C for 5 min, resuspended in PBS + 0.04% BSA and passed through a Flowmi tip strainer (Fisher Scientific) to yield a single-cell suspension. The quality of the samples was assessed using a Luna cell counter (Logos Biosystems). ScRNA-seq was performed using the 10x Genomics Chromium Single Cell 3'RNA-seq platform at the Institute of Molecular Medicine Finland (FIMM). The raw data were mapped to the same customized human reference genome (GRCh38.99 + pCXLE-gw) using Cell Ranger (10x Genomics, v.3.1.0). Empty droplets were removed with DropletUtils (Lun et al., 2018). The remaining droplets were filtered to exclude cells with less than 2000 detected genes, and cells with a high mitochondrial fraction (>50 %). Transcripts that were detected in less than 10 cells were also excluded. The UMI counts were analyzed with R (R Core Team, 2019) using Seurat (version 3.1.4) (Stuart et al., 2019). The data were normalized and the 1000 most variable features per sample were identified (using FindVariableFeatures). The data were scaled (using ScaleData), and the top 50 principal components were extracted. The PCA matrix was "harmonized" using Harmony (Korsunsky et al., 2019) to reduce sample specific biases, with sample ID as the main variable, theta-value set to 2, using 50 clusters and maximum iterations for clusters set to 40 and 15 for the entire harmony series. The harmonized PCA values were then used as input for a 2D and 3D UMAP. Cell clusters were identified with Seurat functions (FindNeighbors, FindClusters and BuildClusterTree) with the

resolution set to 0.04 and the rest of the settings set to default. For pseudotime analysis we used monocle 3 (v0.2.2) (Trapnell et al., 2014) in combination with tradeSeq (Van den Berge et al., 2020), as well as diffusion maps using destiny (v3.0.1) (Angerer et al., 2016). Expression scores of pluripotency genes were calculated using the AddModuleScore function of Seurat. Differential gene expression analysis between different conditions was performed with the FindMarkers function of Seurat with default settings, and genes with Bonferroni adjusted *P*-value < 0.01 (Wilcoxon rank-sum test) were considered as differentially expressed. Pearson correlation coefficients between cells were calculated using the normalized expression values of 2,000 most variable genes among the mid-reprogramming samples. Annotation of cell identity for the mid-reprogramming samples was performed using SingleR (v1.0.6) (Aran et al., 2019) with Human Primary Cell Atlas (Mabbott et al., 2013) as the reference.

Supplemental references

Angerer, P., Haghverdi, L., Büttner, M., Theis, F.J., Marr, C., and Buettner, F. (2016). Destiny: Diffusion maps for large-scale single-cell data in R. Bioinformatics *32*, 1241–1243. 10.1093/bioinformatics/btv715

Aran, D., Looney, A.P., Liu, L., Wu, E., Fong, V., Hsu, A., Chak, S., Naikawadi, R.P., Wolters, P.J., Abate, A.R., et al. (2019). Reference-based analysis of lung single-cell sequencing reveals a transitional profibrotic macrophage. Nat. Immunol. *20*, 163–172. 10.1038/s41590-018-0276-y

Balboa, D., Weltner, J., Eurola, S., Trokovic, R., Wartiovaara, K., and Otonkoski, T. (2015). Conditionally Stabilized dCas9 Activator for Controlling Gene Expression in Human Cell Reprogramming and Differentiation. Stem Cell Reports *5*, 448–459. 10.1016/j.stemcr.2015.08.001

Van den Berge, K., Roux de Bézieux, H., Street, K., Saelens, W., Cannoodt, R., Saeys, Y., Dudoit, S., and Clement, L. (2020). Trajectory-based differential expression analysis for single-cell sequencing data. Nat. Commun. *11*, 1–13. 10.1038/s41467-020-14766-3

Carcamo-Orive, I., Hoffman, G.E., Cundiff, P., Beckmann, N.D., Souza, S.D., Knowles, J.W., Patel, A., Papatsenko, D., Reaven, G.M., Whalen, S., et al. (2017). Analysis of transcriptional variability in a large human iPSC library reveals genetic and non-genetic determinants of heterogeneity. Cell Stem Cell *20*, 518–532. 10.1016/j.stem.2016.11.005

Dobin, A., Davis, C.A., Schlesinger, F., Drenkow, J., Zaleski, C., Jha, S., Batut, P., Chaisson, M., and Gingeras, T.R. (2013). STAR: Ultrafast universal RNA-seq aligner. Bioinformatics *29*, 15–21. 10.1093/bioinformatics/bts635

Kaisers, W., Schwender, H., and Schaal, H. (2017). Sample size estimation for detection of splicing events in transcriptome sequencing data. Int. J. Mol. Sci. *18,* 1-17. 10.3390/ijms18091900

Kilpinen, H., Goncalves, A., Leha, A., Afzal, V., Alasoo, K., Ashford, S., Bala, S., Bensaddek, D., Casale, F.P., Oliver, J., et al. (2017). Common genetic variation drives molecular heterogeneity in human iPSCs. Nature *546*, 370–375. 10.1038/nature22403

Korsunsky, I., Millard, N., Fan, J., Slowikowski, K., Zhang, F., Wei, K., Baglaenko, Y., Brenner, M., Loh, P. ru, and Raychaudhuri, S. (2019). Fast, sensitive and accurate integration of single-cell data with Harmony. Nat. Methods *16*, 1289–1296. 10.1038/s41592-019-0619-0

Liao, Y., Smyth, G.K., and Shi, W. (2019). The R package Rsubread is easier, faster, cheaper and better for alignment and quantification of RNA sequencing reads. Nucleic Acids Res. *47,* 1-9. 10.1093/nar/gkz114

Love, M.I., Huber, W., and Anders, S. (2014). Moderated estimation of fold change and dispersion for RNA-seq data with DESeq2. Genome Biol. *15*, 1–21. 10.1186/s13059-014-0550-8

Lun, A., Riesenfeld, S., Andrews, T., Gomes, T., and Marioni, J.C. (2018). Distinguishing cells from empty droplets in droplet-based single-cell RNA sequencing data. Genome Biol. *20*, 1-9. 10.1101/234872

Mabbott, N.A., Baillie, J.K., Brown, H., Freeman, T.C., and Hume, D.A. (2013). An expression atlas of human primary cells: Inference of gene function from coexpression networks. BMC Genomics *14,* 1-13. 10.1186/1471-2164-14-632

Martin, M. (2011). Cutadapt removes adapter sequences from high-throughput sequencing reads. EMBnet.Journal *17.1*, 10–12. 10.14806/ej.17.1.200

Ozgyin, L., Horvath, A., Hevessy, Z., and Balint, B.L. (2019). Extensive epigenetic and transcriptomic variability between genetically identical human B-lymphoblastoid cells with implications in pharmacogenomics research. Sci. Rep. *9*, 1–16. 10.1038/s41598-019-40897-9

Stuart, T., Butler, A., Hoffman, P., Hafemeister, C., Papalexi, E., Mauck, W.M., Hao, Y., Stoeckius, M., Smibert, P., and Satija, R. (2019). Comprehensive Integration of Single-Cell Data. Cell *177*, 1888-1902.e21.

Trapnell, C., Cacchiarelli, D., Grimsby, J., Pokharel, P., Li, S., Morse, M., Lennon, N.J., Livak, K.J., Mikkelsen,

T.S., and Rinn, J.L. (2014). The dynamics and regulators of cell fate decisions are revealed by pseudotemporal ordering of single cells. Nat. Biotechnol. *32*, 381–386. 10.1038/nbt.2859

Weltner, J., Balboa, D., Katayama, S., Bespalov, M., Krjutškov, K., Jouhilahti, E.-M., Trokovic, R., Kere, J., and Otonkoski, T. (2018). Human pluripotent reprogramming with CRISPR activators. Nat. Commun. *9*, 1–12. 10.1038/s41467-018-05067-x
