# Supplementary material for: CRISPR activation enables high-fidelity reprogramming into human pluripotent stem cells
Source: Stem Cell Reports. 2022 Jan 20;17(2):413–26. doi: 10.1016/j.stemcr.2021.12.017 (PMC8828555; doi:10.1016/j.stemcr.2021.12.017)
Supplement: Document S2. Article plus supplemental information [file mmc2.pdf]

Stem Cell Reports
Article
OPEN ACCESS

CRISPR activation enables high-fidelity reprogramming into human pluripotent stem cells

Joonas Sokka,[1] Masahito Yoshihara,[2,13] Jouni Kvist,[1,13] Laura Laiho,[1] Andrew Warren,[1] Christian Stadelmann,[1] Eeva-Mari Jouhilahti,[1] Helena Kilpinen,[3,4,5,6] Diego Balboa,[7] Shintaro Katayama,[1,2,8] Aija Kyttälä,[9] Juha Kere,[1,2,8] Timo Otonkoski,[1,10] Jere Weltner,[1,11,12,14] and Ras Trokovic[1,14,*]

[1]Research Programs Unit, Stem cells and Metabolism and Biomedicum Stem Cell Centre, Faculty of Medicine, University of Helsinki, Helsinki 00014, Finland
[2]Department of Biosciences and Nutrition, Karolinska Institutet, Huddinge, Stockholm 14183, Sweden
[3]Helsinki Institute of Life Science (HiLIFE), University of Helsinki, Helsinki 00014, Finland
[4]Faculty of Biological and Environmental Sciences, University of Helsinki, Helsinki 00014, Finland
[5]Institute for Molecular Medicine Finland (FIMM), University of Helsinki, Helsinki 00014, Finland
[6]European Molecular Biology Laboratory, European Bioinformatics Institute, Wellcome Genome Campus, Hinxton, Cambridge CB10 1SD, UK
[7]Centre for Genomic Regulation (CRG), Barcelona Institute of Science and Technology, Barcelona 08003, Spain
[8]Folkhälsan Research Center, Helsinki 00290, Finland
[9]Finnish Institute for Health and Welfare (THL), THL Biobank, Helsinki 00290, Finland
[10]Children's Hospital, Helsinki University Central Hospital, University of Helsinki, Helsinki 00290, Finland
[11]Department of Clinical Science, Intervention, and Technology, Karolinska Institutet, Huddinge, Stockholm 14186, Sweden
[12]Division of Obstetrics and Gynecology, Karolinska Universitets Sjukhuset, Huddinge, Stockholm 14186, Sweden
[13]These authors contributed equally
[14]Senior author
*Correspondence: ras.trokovic@helsinki.fi
https://doi.org/10.1016/j.stemcr.2021.12.017

SUMMARY

Conventional reprogramming methods rely on the ectopic expression of transcription factors to reprogram somatic cells into induced pluripotent stem cells (iPSCs). The forced expression of transcription factors may lead to off-target gene activation and heterogeneous reprogramming, resulting in the emergence of alternative cell types and aberrant iPSCs. Activation of endogenous pluripotency factors by CRISPR activation (CRISPRa) can reduce this heterogeneity. Here, we describe a high-efficiency reprogramming of human somatic cells into iPSCs using optimized CRISPRa. Efficient reprogramming was dependent on the additional targeting of the embryo genome activation-enriched Alu-motif and the miR-302/367 locus. Single-cell transcriptome analysis revealed that the optimized CRISPRa reprogrammed cells more directly and specifically into the pluripotent state when compared to the conventional reprogramming method. These findings support the use of CRISPRa for high-quality pluripotent reprogramming of human cells.

INTRODUCTION

CRISPR activation (CRISPRa) uses a catalytically inactivated form of Cas9 (dCas9) fused with a transactivator domain that enables the activation of transcription from endogenous promoters (Bikard et al., 2013). Advantages of CRISPRa over conventional reprogramming (Fusaki et al., 2009; Okita et al., 2011; Warren et al., 2010) include the direct transcriptional activation from endogenous loci, high multiplexing capability, and the potential to target non-coding regulatory elements. We have recently shown reprogramming of human fibroblasts by CRISPRa targeting the promoters of *OCT4* (*POU5F1*), *SOX2*, *KLF4*, *MYC*, and *LIN28A* and an Alu-motif enriched near promoter regions of genes expressed during embryo genome activation (EEA motif [embryo genome activation-enriched Alu-motif]) (Weltner et al., 2018). However, low reprogramming efficiency hampers the use of CRISPRa. Several small molecules and genes, including pluripotent stem cell micro RNAs (miRNA), can improve reprogramming efficiency (Subramanyam et al., 2011). The miRNA cluster miR-302/367 is expressed at high levels in human embryonic stem cells (hESCs) (Houbaviy et al., 2003) and is known to be sufficient to reprogram somatic cells to pluripotency (Anokye-Danso et al., 2011; Miyoshi et al., 2011). Therefore, the targeted activation of miR-302/367 expression with CRISPRa could improve CRISPRa reprogramming efficiency.

As CRISPRa mediated reprogramming is a novel method for inducing pluripotency, its reprogramming trajectories are as yet unknown. Recent advances in single-cell RNA sequencing (scRNA-seq) technology have facilitated our understanding of reprogramming processes and revealed the importance of the early embryonic programs for successful reprogramming (Cacchiarelli et al., 2015; Francesconi et al., 2019; Liu et al., 2020; Schiebinger et al., 2019; Tran et al., 2019). However, low efficiency and high background of non-reprogrammed cells can obscure the transcriptional analysis of fibroblast CRISPRa reprogramming. This problem could be overcome by using human lymphoblastoid cell lines (LCLs), which are generated by Epstein-Barr virus transformation of B lymphocytes (Neitzel, 1986). *In vitro* LCL cultures grow in suspension, while

This is an open access article under the CC BY license (http://creativecommons.org/licenses/by/4.0/).

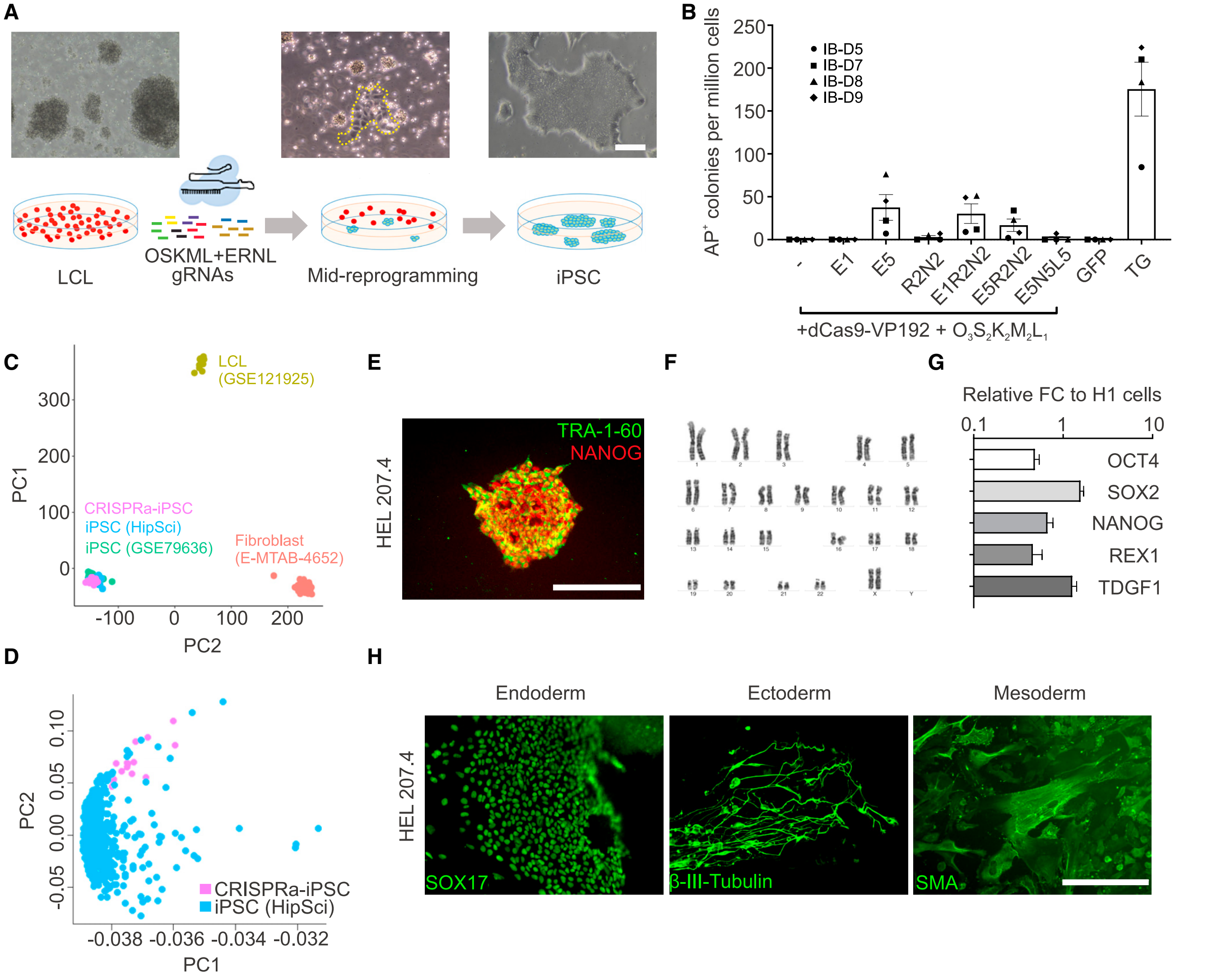

Figure 1. CRISPRa reprogramming of LCL

(A) Schematic representation of the LCL reprogramming protocol with bright-field images from different reprogramming stages. Attached reprogramming intermediates at day 10 are encircled with yellow ticks. Scale bar, 400 μm.

(B) Reprogramming efficiency of LCL from 4 different donors (IB-D5, IB-D7, IB-D8, and IB-D9). The most efficient CRISPRa condition, E5, averaged 38 colonies, while the conventional transgenic (TG) method averaged 176 colonies. Reprogramming conditions are indicated on the x axis. n = 4; each point represents an independent assay from each of the 4 patient-derived LCLs. Error bars, SEMs. E, EEA, L, LIN28A, N, NANOG, R, REX1; numbers refer to the number of guides. GFP-only containing plasmid was used as a negative reprogramming control.

(C) Validation of CRISPRa iPSC (n = 15 individual cell lines), with PCA showing the bulk RNA-seq data grouping with reference iPSC lines (HipSci, GEO: GSE79636) and away from LCL (GEO: GSE121925) and fibroblast (E-MTAB-4652) cell lines. Samples are listed in Table S2.

(D) Further validation with PCA by comparing CRISPRa iPSC (n = 15 individual cell lines) and HipSci iPSC lines (n = 661 individual cell lines).

(E) Immunocytochemistry of undifferentiated hESC markers NANOG and TRA-1-60 in CRISPRa iPSC line HEL207.4. Scale bar, 200 μm. See also Figure S1B. Antibodies and primers used in the article are listed in Table S3.

(F) Normal karyotype of iPSC line HEL207.4. See also Figure S1D.

(G) Expression of selected hESC markers from 5 different CRISPRa-iPSC lines compared to the H1 hESC. n = 5; each replicate is from individual iPSC lines; error bars, SEMs.

(H) Multilineage differentiation of HEL207.4 cells shown by immunostaining for endodermal (SOX17), ectodermal (β-III-tubulin) and mesodermal (smooth muscle actin, SMA) germ layer derivatives. Scale bar, 200 μm. See also Figure S1E.

emerging stem cell colonies attach to the culture surface, providing a simple means for the specific enrichment of the cells undergoing reprogramming. Furthermore, vast collections of LCLs are stored in biobank repositories, providing a virtually unlimited source of reprogramming material due to the immortal nature of these cells (Sie et al., 2009).

In this study, we showed that simultaneous targeting of the EEA motif and the promoter of the miR-302/367 cluster enhanced the reprogramming efficiency of fibroblasts and LCL cells and accelerated the kinetics of induced pluripotent stem cell (iPSC) formation. Using scRNA-seq analysis, we profiled conventional and three combinations of CRISPRa reprogramming across different time points. We found that the cells reprogrammed using the CRISPRa progress to the pluripotent state with high fidelity showing a uniform expression of pluripotency genes and minimal heterogeneity. This is in contrast to the conventional reprogramming, which leads to a longer reprogramming route, often resulting in alternative cell types. These results support the use of CRISPRa for improving the quality of human pluripotent reprogramming.

RESULTS

CRISPRa reprogramming of LCL

We began by validating the CRISPRa system for the reprogramming of LCL. The attachment of reprogramming intermediates to the culture plates was observed by reprogramming day 10, after which unattached cells could be removed (Figure 1A). To test for different factor combinations, CRISPRa targeting *NANOG*, *REX1* (*ZFP42*), *LIN28A*, and EEA motif in addition to the basal reprogramming factor guides for *OCT4*, *SOX2*, *KLF4*, *MYC*, and *LIN28A* that were efficient in fibroblast reprogramming (Weltner et al., 2018) were used. The highest reprogramming efficiency was observed with additional EEA motif targeting by using a combination of five guides (hereafter referred to as CRISPRa + E) (Figures 1B and S1A). We opted to use multiple EEA motif targeting guides for the rest of the work, over *NANOG* and *REX1* targeting, to track reprogramming progress by endogenous *NANOG* activation with immunocytochemistry.

We then confirmed the pluripotency of generated CRISPRa iPSC lines. Principal-component analysis (PCA) of bulk RNA-seq of 15 CRISPRa iPSC lines from 5 LCL and 2 fibroblast donors demonstrated that all CRISPRa iPSC lines grouped with previously published iPSC lines generated from blood and fibroblast cells using conventional methods (Carcamo-Orive et al., 2017; Kilpinen et al., 2017) and distinct from LCL and fibroblasts (Kaisers et al., 2017; Ozgyin et al., 2019) (Figure 1C). CRISPRa iPSC lines were then compared more closely with a total of 661 Human Induced Pluripotent Stem Cells Initiative (HipSci) iPSC lines, which showed that our cell lines grouped closely together with the HipSci lines (Kilpinen et al., 2017) (Figure 1D). Further characterization showed a normal karyotype, expression of undifferentiated hESC markers, differentiation into three embryonic germ layer derivatives, and the loss of episomal reprogramming plasmids (Figures 1E–1H and S1B–S1E), supporting the notion that the LCL had been reprogrammed into *bona fide* iPSC.

miR-302/367 promoter targeting improves CRISPRa reprogramming efficiency

To improve the CRISPRa reprogramming efficiency, we targeted the promoter of the miR-302/367 cluster home gene *MIR302CHG* (Figures 2A and S2A). Multiple primary fibroblast lines were transfected with the miR-302/367 targeting guides on top of CRISPRa + E (hereafter referred to as CRISPRa + ME) along with the transgenic (TG) and CRISPRa + E conditions. CRISPRa + ME significantly increased the reprogramming efficiency and colony size when compared to other reprogramming conditions (Figures 2B and S2B–S2D). CRISPRa + ME was the only reprogramming condition that properly induced iPSC colonies from an 83-year-old male-derived primary fibroblast line M83 known for being difficult to reprogram (Trokovic et al., 2015) (Figures 2B, S2C, and S2D). LCL reprogramming with the dCas9 activator plasmid and miR-302/367 guides alone did not yield any colonies (Figure S2E), but the targeting of miR-302/367 cluster on top of basal CRISPRa (hereafter referred to as CRISPRa + M) increased LCL reprogramming efficiency 6-fold (mean 169 alkaline phosphatase-positive [AP$^+$] colonies, n = 6 independent experiments) compared to CRISPRa + E. Reprogramming efficiency further increased up to 8-fold (mean 228 AP$^+$ colonies, n = 6 independent experiments) with the combined CRISPRa + ME condition (Figure 2C).

To define the time point for analyzing the reprogramming with scRNA-seq, we characterized the attachment and growth of the reprogramming intermediates. As attached reprogramming intermediates could be seen on day 10, the cells that were still in suspension at this stage were re-plated. After 1 week none of the re-plated cells had started to form iPSC-like colonies, suggesting that most of the reprogramming cells had already attached by day 10 (Figure S2F). Live cell imaging of the reprogramming cell cultures from days 15–17 revealed significantly larger colony sizes in CRISPRa + ME compared to the TG condition (Figure 2D) and increased cell numbers (Figures 2E and S2G). Staining for hESC markers NANOG and TRA-1-60 showed NANOG$^+$ and TRA-1-60$^+$ colonies emerging by day 14 in the CRISPRa + E condition, while the CRISPRa + ME condition presented NANOG$^+$ and TRA-1-60$^+$ colonies already at day 13 (Figure 2F). This suggested accelerated

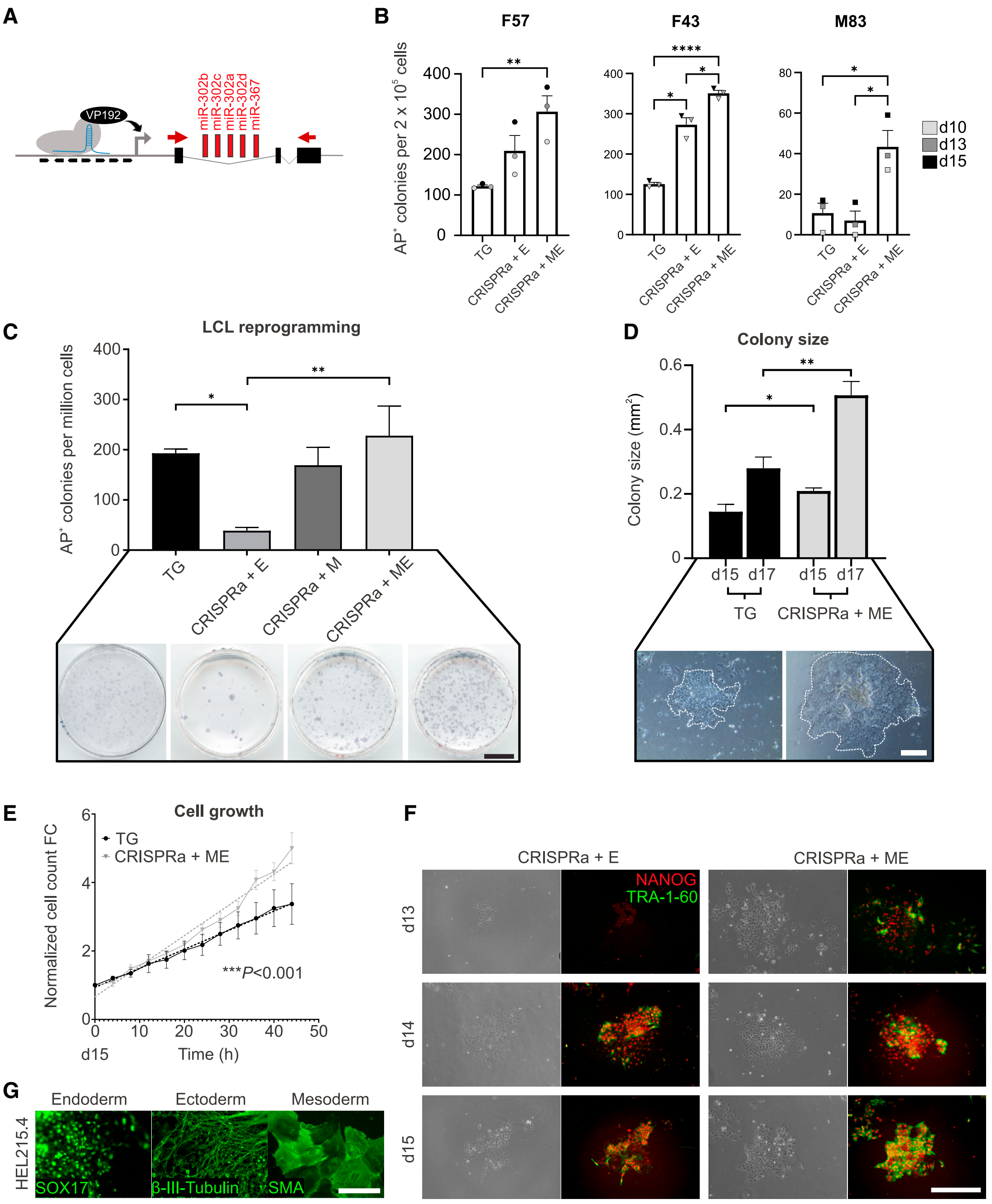

Figure 2. miR-302/367 promoter targeting improves CRISPRa reprogramming efficiency

(A) Schematic representation of the CRISPRa activation of miR-302/367 cluster home gene (MIR302CHG). Red arrows show the locations of the qPCR primers. Guides targeting the MIR302CHG are listed in Table S4.

(B) Reprogramming efficiency of primary fibroblasts using 3 separate donor lines, F57, F43, and M83, on days 10, 13, and 15. Reprogramming conditions are indicated on the x axis. n = 3; each point represents an independent assay in which cells were counted on either day 10, 13, or 15, as indicated by the legend; error bars, SEMs; p values were calculated with repeated measures 1-way ANOVA post-hoc Tukey's test, *p < 0.05, **p < 0.01, ****p < 0.0001.

(C) Reprogramming efficiency of LCL. Reprogramming conditions are indicated on the x axis. n = 6 independent experiments; error bars, SEMs; p values were calculated with 1-way ANOVA post-hoc Tukey's test, *p < 0.05, **p < 0.01. Representative AP-stained culture plates from each reprogramming condition are shown below. Scale bar, 1 cm.

(legend continued on next page)

reprogramming kinetics of the miR-302/367 CRISPRa-targeted cells and that day 15 would be an optimal time point to explore the transcriptomic profiles of reprogramming intermediates by scRNA-seq analysis. Finally, to verify that the CRISPRa + ME reprogramming in LCL was robust in producing pluripotent cells, and not due to a single donor effect, four additional LCL donor lines were reprogrammed using CRISPRa + ME. All of the donor lines differentiated properly into all three embryonic germ layer derivatives, confirming the pluripotent nature of the cells (Figures 2G and S2H).

scRNA-seq captures the progression of CRISPRa reprogramming

To investigate changes in transcription at the single-cell level, we prepared samples for scRNA-seq at various time points of the reprogramming process. To mitigate the effects of genetic background, cells were collected from the same donor reprogrammed using the TG and three different CRISPRa (CRISPRa + E, + M, + ME) conditions at reprogramming days 0 (starting LCL) and 15 (mid-reprogramming cells), as well as from passage 1 and 10 iPSCs (Figure 3A). Characterization of the cell populations that arose during reprogramming was performed by unsupervised clustering analysis, which identified seven cell clusters (Figure 3B; Table S5). LCL confined into cluster 1, separate from the other clusters. Notably, while the mid-reprogramming cells from 3 CRISPRa conditions were located mostly in cluster 2, the TG mid-reprogramming cells clustered separately between clusters 4, 5, and 6. Passage 1 and 10 iPSCs were found in clusters 6 and 7, with over half of the passage 10 iPSC localizing in cluster 7, marking it as the endpoint of the reprogramming process (Figures 3C and 3D). Interestingly, 14% of mid-reprogramming CRISPRa + ME cells were in iPSC cluster 7, indicating that these cells may proceed toward the iPSC state faster than the other conditions (Figures 3C and 3D).

We then integrated the scRNA-seq to bulk RNA-seq data of published LCL (Ozgyin et al., 2019) and iPSC (Carcamo-Orive et al., 2017; Kilpinen et al., 2017) datasets and used PCA to visualize the relationship between clusters (Figure S3A). As expected, cluster 1 grouped close to the reference LCL, cluster 7 grouped closest to the reference iPSC samples, while the mid-reprogramming clusters grouped between the LCL and iPSC (Figure S3A). The cell identity of cluster 1 was further confirmed using the expression of well-known LCL markers *ENTPD1*, *FCER2*, *CD70*, and *LFA3* (Rajesh et al., 2011) (Figures 3E and S3B). These markers were downregulated in mid-reprogramming and iPSC clusters, consistent with previous reports (Rajesh et al., 2011). The known reprogramming and hESC markers *L1TD1*, *OCT4*, *NANOG*, *SOX2*, *TDGF1*, and *REX1* were detected in the mid-reprogramming and iPSC clusters and were almost completely absent from the LCL cluster, as expected (Figures 3E and S3B).

However, a notable exception was that these hESC markers were mostly absent in TG mid-reprogramming cluster 4 (Figures 3E and S3B). The TG mid-reprogramming cells with high hESC marker expression were instead located in iPSC cluster 6 as well as in a small cluster, 5, consisting almost solely of mid-reprogramming TG cells (Figures 3C–3E). To detect the presence of episomal vectors, we used reads mapped to the transcribed elements in the plasmid backbone (WPRE). Retention of the high expression of episomal vectors in the transgene mid-reprogramming was observed while they were mostly absent from the CRISPRa conditions (Figure 3F). To further distinguish the pluripotency signature between samples, gene expression of 140 (Table S6) well-known hESC markers were analyzed (Figure 3G). In particular, TG mid-reprogramming displayed a bimodal expression of hESC markers when compared to CRISPRa conditions (Figure 3G). This indicated the presence of a heterogeneous mixture of pluripotent-like and non-pluripotent cells in the TG reprogramming conditions. Trajectory analysis on cells excluding LCL cluster 1 revealed that cells in the TG condition took an alternative trajectory early on, leading to multiple endpoints, while the CRISPRa + ME condition followed a more direct route to the iPSC state (Figures 3H and S4A). Further analysis of the TG mid-reprogramming cells showed the loss of transgene expression along with pseudotime progression and a bifurcation of the cells into

(D) Measurement of average iPSC-like colony size at days 15 and 17. n = 6 independent experiments; error bars, SEMs; p value was calculated with Student's t test, $*p < 0.05$, $**p < 0.01$. Bright-field images of iPSC-like colonies in TG and CRISPRa + ME conditions at day 17 are shown below. Colony edges have been highlighted with white tick marks. Scale bar, 400 μm.

(E) Growth rate of the iPSC-like cells in TG and CRISPRa + ME conditions from day 15 onward. Cell counts are normalized to the day 15 starting point. n = 6 independent experiments; error bars, SEMs. Significance of the difference between the linear regression lines (R^2 TG = 0.4965, CRISPRa + ME = 0.8475) was calculated using GraphPad Prism's linear regression analysis equivalent to ANCOVA, $***p < 0.001$. See also Figure S2G.

(F) Immunocytochemistry images showing the hESC markers NANOG and TRA-1-60 in CRISPRa + EEA and CRISPRa + ME reprogramming at days 13–15. Scale bar, 400 μm.

(G) Multilineage differentiation of CRISPRa + ME reprogrammed HEL215.4 iPSC line shown by immunostaining for endodermal (SOX17), ectodermal (β-III-tubulin), and mesodermal (smooth muscle actin, SMA) germ layer derivatives. Scale bar, 200 μm. See also Figure S2H.

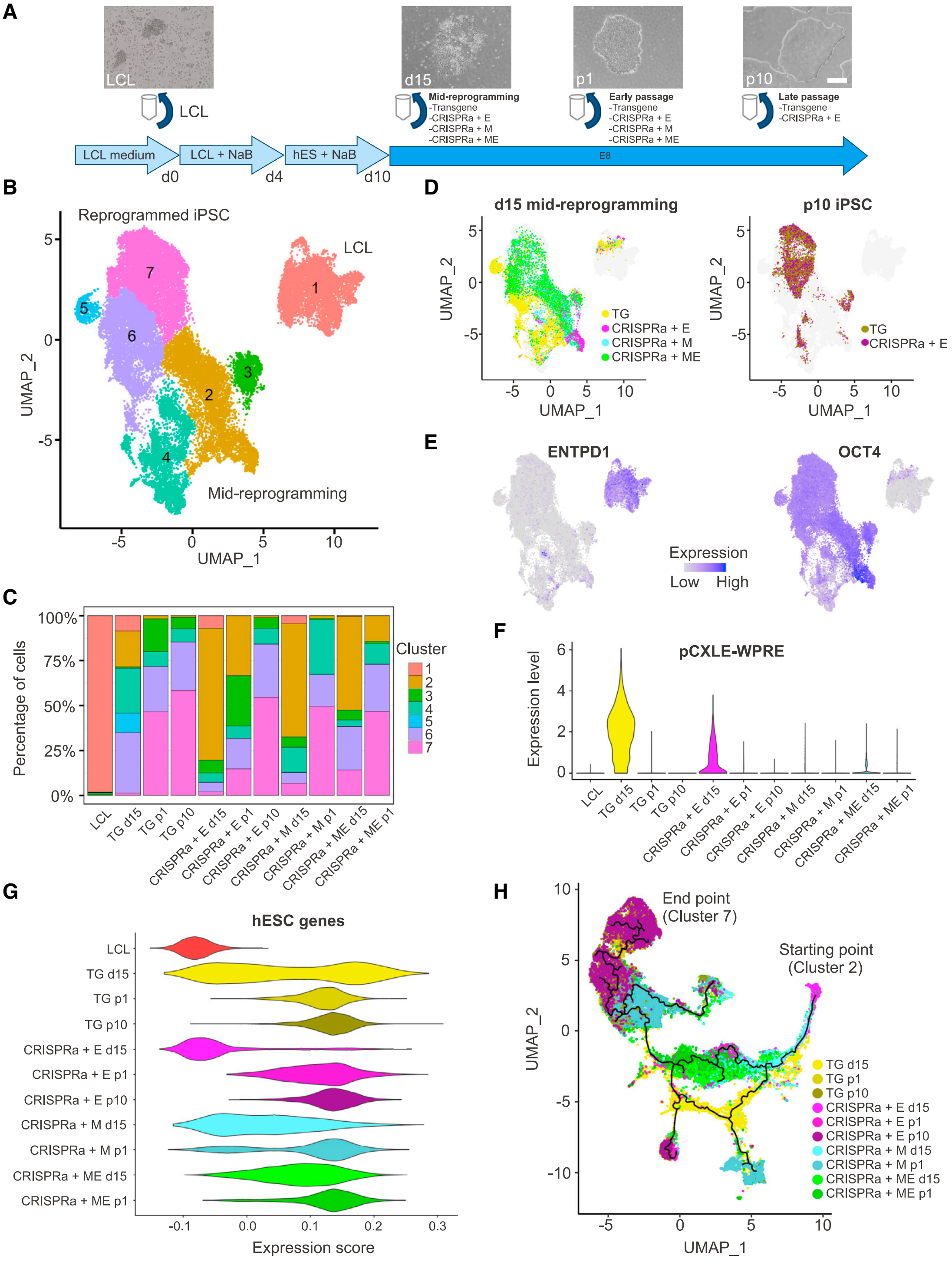

Figure 3. scRNA-seq captures the progression of CRISPRa reprogramming

(A) Schematic representation of the time-resolved scRNA-seq sample collection strategy.

(B) UMAP plot representing the 7 clusters across 32,758 cells from different reprogramming conditions and time points.

(C) Cell composition across the 7 clusters in each sample. Colors indicate each cluster as seen in (B). Individual cluster cell counts are listed in Table S5.

(D) UMAPs showing the distributions of mid-reprogramming and passage 10 cells. Gray dots represent cells from other samples.

(legend continued on next page)

OCT4$^+$ (clusters 5–7) and *OCT4*$^-$ (cluster 4) populations (Figures S4C and S4D), suggesting that loss of transgene expression before endogenous pluripotency factor activation may hinder proper reprogramming progression. These results support the notion that CRISPRa + ME progress to the iPSC state with improved fidelity and kinetics, while TG cells are more dependent on their episomal vectors and thus take a longer reprogramming route, resulting in alternative endpoints.

miR-302/367 and EEA motif act synergistically to promote pluripotency at the mid-reprogramming stage

We then characterized the day 15 mid-reprogramming samples in more detail. Cluster 2 contained a subpopulation of cells with a high expression of the lymphocyte marker *CD52* (Hale et al., 1990), which consisted primarily of CRISPRa + E cells (Figures 4A–4C). Cluster 2 cells located closer to the iPSC clusters showed expression of the primed hESC surface marker *CD90* (*THY1*) (Collier et al., 2017), but still lacked the expression of another marker, *EPCAM*, which is involved in the epithelialization process (Huang et al., 2011). This suggests that these cells were intermediate reprogramming cells (Figures 4A, 4B, and S4H). By clustering only the day 15 sample cells, distinct clusters for naive and primed cells could be detected (Figures S4F and S4I).

The mid-reprogramming samples appeared to cluster in different parts of the UMAP plot with different CRISPRa reprogramming conditions (Figures 4C and S4G). Therefore, the progression to the iPSC state was estimated by applying a diffusion pseudotime analysis on all of the samples, including LCL and iPSC (Figure S4B). Among the mid-reprogramming samples, CRISPRa + ME progressed to the iPSC state faster compared to the other conditions, while most of the CRISPRa + E remained stuck at the beginning of reprogramming (Figures 4D and S4B). These results suggest that the endogenous activation of miR-302/367 helps to induce the cells out of the initial blood cell-like state.

To evaluate the gene expression changes between different reprogramming conditions at the mid-reprogramming stage, the gene expression profile of CRISPRa + ME was compared with the other three conditions (Figure 4E). Higher expression of hESC genes such as *LIN28*A and *L1TD1* and the surface marker *CD90* was observed in the CRISPRa + ME compared with CRISPRa + E. The miR-302/367 cluster targeting influenced the expression of approximately three times more genes compared to the EEA targeting, including multiple pluripotency-associated genes (Figure 4E). Thus, the miR-302/367 targeting may help lower the barrier for the activation of pluripotency-associated genes.

We further investigated the rate of blood cells reprogramming using the expression of genes characteristic for blood cells, such as major histocompatibility complex (MHC) class I human leukocyte antigens (*HLA-A*, *HLA-B*, and *HLA-C*) and an associated gene *B2M*. These MHC class I-associated genes are expressed in almost all nucleated cells (Gussow et al., 1987; Ploegh et al., 1981), but their expression is notably higher in the blood cells (Boegel et al., 2018; Mabbott et al., 2013; Papatheodorou et al., 2020; Thul et al., 2017). The expression of these genes was reduced in the CRISPRa + ME compared to all other mid-reprogramming conditions (Figure 4E). Furthermore, when comparing the expression levels of *B2M* and *HLA-A* to the diffusion map pseudotime, their expression was rapidly decreased in the CRISPRa + ME cells (Figures 4F and S4E). These findings support the notion that the CRISPRa + ME cells lose their initial blood cell-like identity and progress faster toward the iPSC state.

The expression of epithelial cell specific genes *KRT8*, *KRT18* (Fuchs and Weber, 1994), and *EPCAM* were increased in the CRISPRa + ME condition compared to the CRISPRa + M (Figure 4E), suggesting that the EEA motif targeting may aid in epithelialization during reprogramming, in addition to its reported role in aiding in pluripotency factor activation (Weltner et al., 2018). This effect may be of importance, especially in the later stages of reprogramming, as seen by the *EPCAM* expression between CRISPRa + ME and CRISPRa + M (Figures 4A–4C). In summary, our results suggest that the miR-302/367 and EEA motif targeting act synergistically by enhancing the progression of reprogramming from the initial blood cell state and aiding epithelialization.

CRISPRa + ME cells progress to the pluripotent state with improved fidelity

To assess the mid-reprogramming cell heterogeneity observed by microscopy (Figure 5A), we calculated the correlation coefficients of highly variable genes between all

(E) Expression of LCL marker ENTPD1 and hESC marker OCT4. Colors indicate expression levels (blue, high; gray, low).
(F) Violin plot showing the expression of the episomal vector (short plasmid backbone sequence pCXLE-WPRE) across the different reprogramming conditions and time points.
(G) Expression score of 140 selected hESC markers across the different reprogramming conditions and time points.
(H) Pseudotime analysis showing the trajectory of the cells during reprogramming, with cluster 1 excluded. Time points and reprogramming conditions are indicated in the key.
See also Figure S4A.

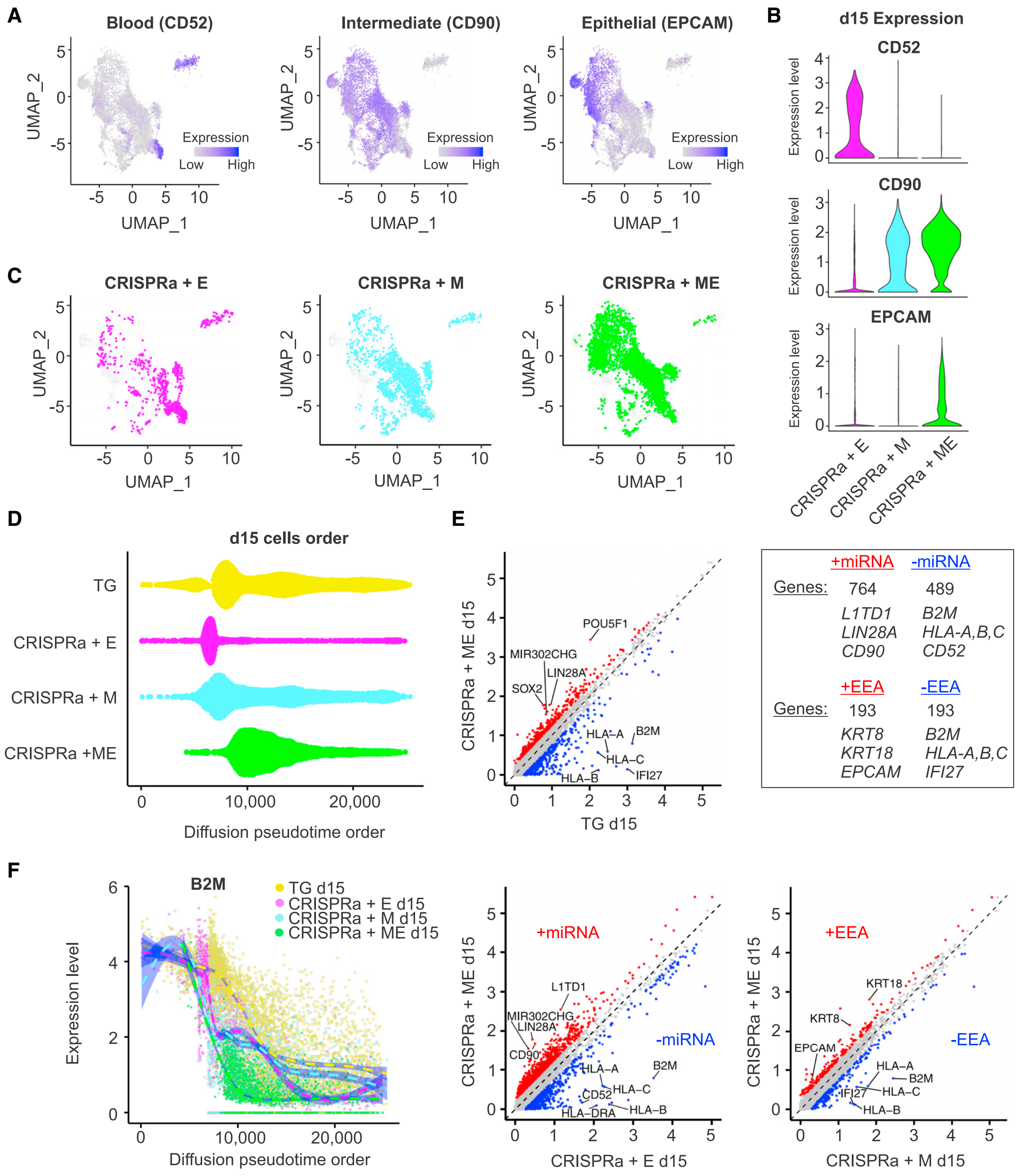

Figure 4. miR-302/367 and EEA motif act synergistically to promote pluripotency at the mid-reprogramming stage

(A) Expression of blood cell marker CD52, intermediate reprogramming marker CD90, and epithelial marker EPCAM in all mid-reprogramming cells. Colors indicate expression levels (blue, high; gray, low).

(B) Violin plots showing the expression levels of CD52, CD90, and EPCAM across the different CRISPRa reprogramming conditions. See also Figure S4H.

(C) UMAPs showing the distribution of mid-reprogramming CRISPRa + E, CRISPRa + M, and CRISPRa + ME cells. Gray dots represent cells from other mid-reprogramming cell samples.

(D) Mid-reprogramming cells ordered by diffusion pseudotime. See also Figure S4B.

(E) Comparison of expression profiles between mid-reprogramming CRISPRa + ME and TG cells (top left), CRISPRa + ME and CRISPRa + E (bottom left), and CRISPRa + ME and CRISPRa + M (bottom right). Log-scaled average expression levels of all cells in each condition are

(legend continued on next page)

cells for each mid-reprogramming condition. CRISPRa + ME had a higher correlation coefficient compared to all of the other conditions, suggesting less heterogeneity among the cells (Figure 5B). The distribution of *OCT4* expression level demonstrated a sharp single peak in CRISPRa + ME, indicating that most of these cells showed similar expression levels of *OCT4* (Figure 5C), whereas other conditions showed broader or bimodal peaks, indicating varying expression levels. To identify the cell populations at the mid-reprogramming stage, cells were annotated against the bulk RNA-seq data of the Human Primary Cell Atlas (Mabbott et al., 2013) using SingleR (Aran et al., 2019) (Figure 5D). A total of 99% of CRISPRa + ME cells showed the highest transcriptional similarity to hESC or iPSC, while CRISPRa + E showed the highest heterogeneity, with ~60% of cells being annotated as blood cells. TG and CRISPRa + M cells had similar heterogeneity profiles, with ~10% of cells annotated as blood cells and another 10% as neuronal cells, indicating differentiation toward alternative cell types (Figure 5D). These results reinforced our findings that the CRISPRa + ME condition reprogrammed the cells toward the iPSC state with greatly improved fidelity.

Finally, to validate our findings from scRNA-seq, we analyzed the mid-reprogramming cells with flow cytometry for the hESC surface marker TRA-1-60 (Pera et al., 2000). The results showed that CRISPRa + ME cells had more uniform TRA-1-60 expression on day 15 when compared to the TG condition (Figures 5E and 5F), supporting the bimodal expression pattern of TG cells. These results demonstrate the use of CRISPRa as an efficient reprogramming tool able to reduce cellular heterogeneity in human iPSC induction.

DISCUSSION

Based on our results, activation of endogenous genes by CRISPRa can be used for efficient reprogramming of fibroblasts and LCL into iPSC. The efficiency was dependent on the additional targeting of the *MIR302CHG* transcript and the EEA motif. Further scRNA-seq analysis of day 15 reprogramming intermediates revealed less heterogeneity in the CRISPRa + ME reprogrammed cells compared to other conditions.

Numerous repositories contain LCL generated from a variety of patients, and conventional reprogramming methods have been used to derive iPSC from them (Barrett et al., 2014; Fujimori et al., 2016; Rajesh et al., 2011; Thomas et al., 2015). The CRISPRa reprogramming method described herein broadens the available tools for using these repositories for disease modeling. Transcriptionally, the CRISPRa iPSCs were indistinguishable from other high-quality iPSC lines generated from the large repositories using conventional TG transcription factor-mediated reprogramming methods (Carcamo-Orive et al., 2017; Kilpinen et al., 2017).

A key aspect of the efficient reprogramming of LCL with CRISPRa was targeting the *MIR302CHG* locus. Although the ectopic overexpression of the miR-302/367 has been reported to reprogram both mouse and human cells into iPSC (Anokye-Danso et al., 2011; Miyoshi et al., 2011), we did not observe any iPSC colonies when only miR-302/367 was targeted. This is possibly due to the modest activation of *MIR302CHG* transcript by CRISPRa compared to the ectopic expression of miRNA used in previous studies. The activation of the *MIR302CHG* transcript promoted the transition of LCL toward iPSC-like cells. Our observations are consistent with the reported role of miR-302/367 in reducing the expression of a number of repressive factors in reprogramming (Subramanyam et al., 2011) and enhancing reprogramming (Kogut et al., 2018). In addition, the enhanced activation of *LIN28A* by miR-302/367 targeting may contribute to a more efficient expression of pluripotency factors by regulating the synthesis of Let-7 miRNA (Ustianenko et al., 2018; Worringer et al., 2014). Activation of additional pluripotency-associated miRNAs may prove to be an efficient way of improving CRISPRa reprogramming. In line with this, a recent article demonstrated that the transcriptional activation of another miRNA cluster on chromosome 19 (C19MC) accelerates human cellular reprogramming (Mong et al., 2020).

Previous studies have described detailed roadmaps of somatic cell reprogramming toward pluripotency (Cacchiarelli et al., 2015; Hussein et al., 2014; Liu et al., 2020; Schiebinger et al., 2019; Takahashi et al., 2014; Takahashi and Yamanaka, 2016; Wang et al., 2018). These roadmaps have pinpointed the heterogeneity of the reprogramming process and the transient cell populations and off-target cell types emerging during the process. Our day 15 reprogramming samples replicate the heterogeneity and reprogramming progression described previously, spanning from somatic-like cells to iPSC-like cells. Importantly, this mid-reprogramming sample time point enabled us to assess the effect of different reprogramming methods on the reprogramming of human LCL.

plotted. At top right, the number of significantly highly expressed genes in CRISPRa conditions with or without miR-302/367 or EEA targeting are shown, with some of the top hits listed. These genes are marked as red or blue in the plots.
(F) Diffusion pseudotime combined with the expression level of B2M in the mid-reprogramming samples. Dashed lines and blue shades are fitted generalized additive model, with 95% confidence interval in each condition. Each color represents the different reprogramming condition.

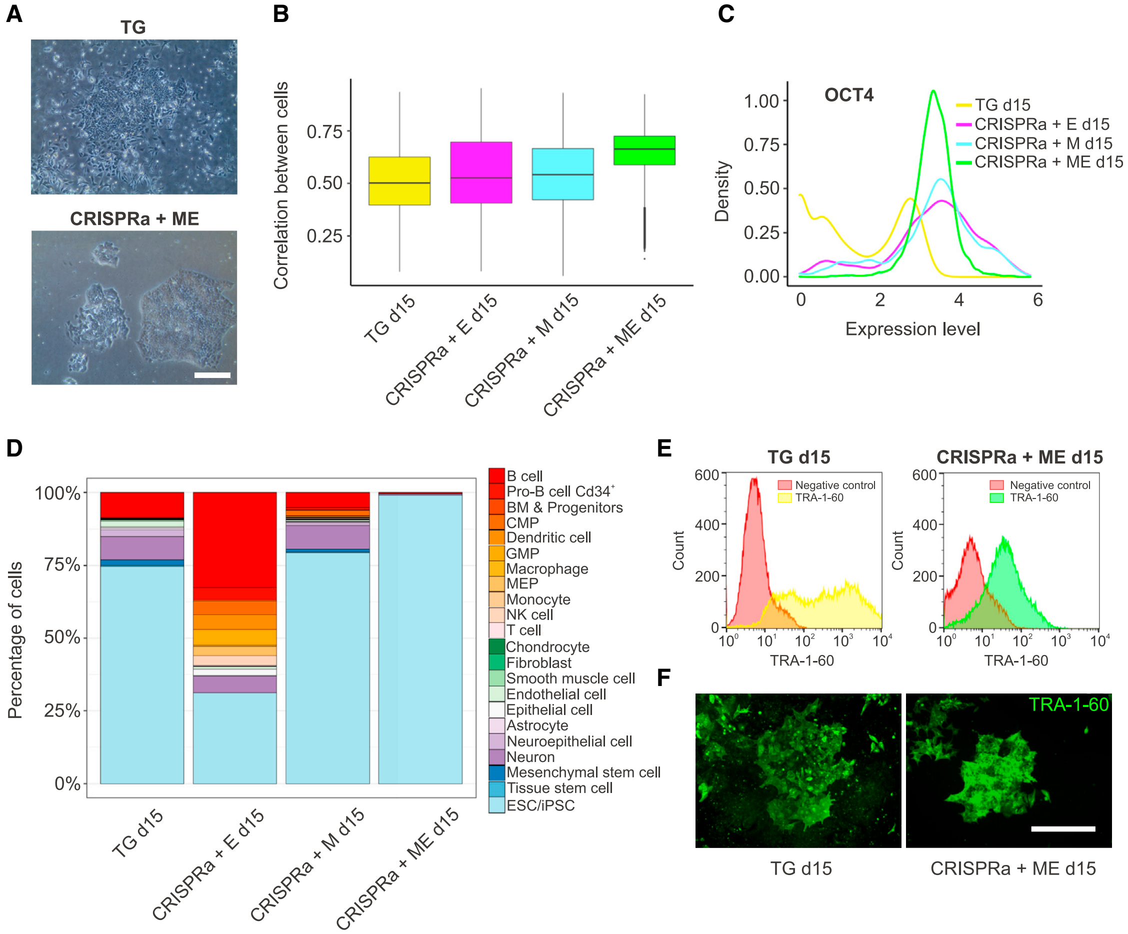

Figure 5. CRISPRa + ME cells progress to the pluripotent state with improved fidelity

(A) Morphology of colonies during TG and CRISPRa + ME mid-reprogramming. Scale bar, 400 μm.

(B) Correlation coefficients of gene expression profile among cells in all mid-reprogramming conditions.

(C) Distribution of OCT4 expression level in cells from all mid-reprogramming conditions.

(D) Cell identity annotation in all mid-reprogramming conditions using SingleR. BM, bone marrow; CMP, common myeloid progenitors; GMP, granulocyte monocyte progenitors; MEP, megakaryocyte-erythroid progenitor cell.

(E) Flow cytometry analysis of hESC marker TRA-1-60 in TG and CRISPRa + ME mid-reprogramming cells. Red, negative control.

(F) Immunocytochemistry images showing TRA-1-60 expression in TG and CRISPRa + ME mid-reprogramming cells. Scale bar, 400 μm.

The best reprogramming outcome was observed by concurrent targeting of both miR-302/367 and EEA motif. The reprogramming effect of the EEA motif targeting has previously been linked to its contribution to the activation of *NANOG* and *REX1* (Weltner et al., 2018). The scRNA-seq data from the LCL suggest that the EEA motif targeting additionally promotes epithelialization, which is supported by the increased *KRT8/18* expression and higher proportion of *EPCAM*+ cells at the later time point clusters (Fuchs and Weber, 1994; Huang et al., 2011). *KRT18* expression appears to be directly activated by CRISPRa targeting of the EEA motif, similar to what we have observed previously in fibroblasts (Weltner et al., 2018). Intriguingly, this also suggests a role for the Alu-KRT18 axis in controlling human early embryo development, as KRT8 and KRT18 were recently described as the first fate determinants that drive early embryo lineage specification (Lim et al., 2020), and the EEA motif was originally detected from embryo-sequencing data (Töhönen et al., 2015). However, further studies are still required to decipher the exact mechanism behind the role of miRNA and EEA targeting on improving the reprogramming efficiency.

Importantly, the optimized CRISPRa reprogramming, with *MIR302CHG* and EEA motif targeting, seems to proceed more homogeneously than conventional reprogramming. Analysis of the cells being reprogrammed revealed differences, particularly in the expression of the

endogenous reprogramming factors. The barrier for the activation of endogenous pluripotency factors may thus contribute to the divergent route that TG reprogramming cells seem to take to pluripotency. However, high levels of TG *OCT4* expression may be one additional explanation for the increased heterogeneity of the TG samples. A recent study reported that ectopic *OCT4* expression is detrimental to the generation of high-quality mouse iPSCs, due to the activation of off-target genes (Velychko et al., 2019). Thus, the activation of endogenous factors may result in improved iPSC quality through a more deterministic reprogramming process. This also supports the use of CRISPRa for improving the specificity of pluripotent reprogramming. Alternatively, targeting of additional factors specific to the alternative cell-type clusters could promote more specific derivation of other reprogrammed cell types from the reprogramming intermediates using CRISPRa (e.g., induced primitive endoderm or trophoblast cells).

In conclusion, the optimized CRISPRa approach reprograms cells toward pluripotency efficiently and with improved fidelity. These findings support the use of CRISPRa to improve the quality of human pluripotent reprogramming.

EXPERIMENTAL PROCEDURES

Additional methods and more in-detail descriptions of bulk and scRNA-seq can be found in the supplemental experimental procedures.

Ethical consent

The generation of the human iPSC lines was approved by the Coordinating Ethics Committee of the Helsinki and Uusimaa Hospital District (no. 423/13/03/00/08) with informed consent of the donors. LCLs were obtained from THL Biobank (Finnish Institute of Health and Welfare, www.thl.fi/biobank), and the experiments were performed according to the contract and in compliance with the general terms of the THL Biobank (application no. BB2016_56, amendment BB2018_33).

Cell culture

LCL were cultured in LCL medium (RPMI 1640 with GlutaMAX [Thermo Fisher Scientific]) supplemented with 15% fetal bovine serum (FBS) (Life Technologies) and 1 μM sodium pyruvate. Human embryonic kidney cells (HEK293, ATCC [American Type Culture Collection] line CRL-1573) and human fibroblasts were cultured in fibroblast medium (Dulbecco's Modified Eagle Medium [DMEM, Sigma-Aldrich, D6546]) supplemented with 10% FBS, 2 mM GlutaMAX (GIBCO), and 100 μL/mL penicillin-streptomycin (Sigma-Aldrich). iPSCs were cultured in plates coated with Matrigel (Corning) in E8 (GIBCO) or E8 Flex (GIBCO) medium. The medium was changed every other day. All of the cells were kept in an incubator at 37°C and 5% CO_2 and were routinely tested for mycoplasma contamination.

CRISPRa and conventional reprogramming of LCL

LCL were passaged the day before reprogramming. On the day of reprogramming (day 0), LCLs were dissociated into single cells by trituration and washed in PBS (Thermo Fisher Scientific). Cells were electroporated with the Neon transfection system (Invitrogen) using R buffer. A total of 10^6 cells and 6 μg plasmid mixture, containing 1.5 μg dCas9-activator plasmid (Addgene #69535) and 4.5 μg guide plasmids, were electroporated in a 100-μL tip using 1,300 V, 10 ms, and 3× pulse conditions. For conventional reprogramming, a plasmid mixture containing 2 μg of each ectopic expression plasmid (Addgene #27077, #27078, #27080) (Okita et al., 2011) was transfected with the same electroporation conditions as CRISPRa. Electroporated LCLs were then plated onto cell culture dishes in LCL growth medium supplemented with 0.25 mM sodium butyrate (NaB). The GFP expression from the episomal dCas9 activator-plasmid or from the ectopic expression plasmids was visualized the next day to verify a successful electroporation. After 3 days, the cells were passaged onto Matrigel-coated plates. On day 4 after the transfections, the medium was changed to hES medium (DMEM/F12 with GlutaMAX [Life Technologies]), supplemented with 20% KnockOut Serum Replacement (Life Technologies), 0.0915 mM 2-mercaptoethanol (Life Technologies), 1× Non-Essential Amino Acids (Life Technologies), and 6 ng/mL basic fibroblast growth factor (bFGF) (PeproTech) supplemented with 0.25 mM NaB. The cell growth medium was changed to E8 medium at day 10 of reprogramming, and the unattached LCLs were removed at this point. The cells were cultured until day 21, when the colonies were large enough to be manually picked and plated on Matrigel-coated wells in E8 medium. The media were changed every other day. The list of plasmids used for LCL reprogramming is provided in Table S1.

CRISPRa and conventional reprogramming of fibroblasts

Fibroblasts (human foreskin fibroblasts [HFFs, ATCC line CRL-2429] and 43- to 83-year-old donor-derived primary fibroblast lines F72, F57, F43, and M83]) were seeded 4 days before the start of reprogramming. On the day of reprogramming (day 0), cells were detached as single cells from the culture plates with TrypLE Select (GIBCO) and washed with PBS. Cells were electroporated using the Neon transfection system (Invitrogen) and reprogrammed by CRISPRa, as described previously (Weltner et al., 2018; Weltner and Trokovic, 2021). Conventional reprogramming was performed with the same transfection conditions as CRISPRa, using the three ectopic expression plasmids mentioned previously (Addgene #27077, #27078, and #27080) (Okita et al., 2011). Electroporated fibroblasts were plated on Matrigel-coated plates immediately after the transfections. The medium was changed every other day, and on day 4, the fibroblast medium was changed to a 50:50 ratio of fibroblast medium and hES medium, supplemented with 0.25 mM NaB. The cells were kept growing with medium changes every other day, until the formed iPSC colonies were either AP stained or collected for further analysis on days 10, 13, and 15.

Bulk RNA-seq and processing

Total RNA was extracted from 15 iPSC lines (Table S2), and purified using the NucleoSpin RNA Plus kit (Macherey-Nagel). Bulk

RNA-seq was performed as a service at Novagen after the cells passed quality control. The expression profiles of the RNA-seq data were compared to published reference datasets of iPSC, LCL, and fibroblasts (GEO: GSE79636, HipSci, GEO: GSE121926, E-MTAB-4652) (Carcamo-Orive et al., 2017; Kaisers et al., 2017; Kilpinen et al., 2017; Ozgyin et al., 2019) analyzed with the same methods.

Single-cell RNA-seq and processing

Cells were dissociated with Accutase at 37°C for 5 min, resuspended in PBS + 0.04% BSA on ice, and passed through a Flowmi tip strainer (Fisher Scientific) to yield a single-cell suspension. The quality of the samples was assessed using a Luna cell counter (Logos Biosystems). scRNA-seq was performed using the 10X Genomics Chromium Single Cell 3′RNA-seq platform at the Institute of Molecular Medicine Finland (FIMM).

Statistical analysis

Statistical analyses were performed with Student's t test, analysis of variance (ANOVA), and the linear regression slope comparison test (GraphPad Prism version 8.4.2) equivalent to the analysis of covariance (ANCOVA), as described in the figure legends. $p < 0.05$ was considered significant (*$p < 0.05$, **$p < 0.01$, ***$p < 0.001$, ****$p < 0.0001$).

Data and code availability

The accession number for the RNA-seq data reported in this paper isGEO: GSE162530. The rest of the data are available in the main text or in the supplemental information.

SUPPLEMENTAL INFORMATION

Supplemental information can be found online at https://doi.org/10.1016/j.stemcr.2021.12.017.

AUTHOR CONTRIBUTIONS

R.T., J.W., and D.B. were responsible for the conceptualization. J.W. and D.B. were responsible for the methodology. J.S., L.L., C.S., and A.W. were responsible for the investigation. M.Y., J. Kvist, and H.K. were responsible for the bioinformatics analysis. J.S., M.Y., and J. Kvist were responsible for visualization. T.O. and A.K. were responsible for the resources. J.S. was responsible for writing the original draft. All of the authors were involved in reviewing and editing. R.T., J.W., E.-M.J., S.K., J. Kere, and T.O. were responsible for supervision. R.T., J. Kere, and T.O. were responsible for funding acquisition.

CONFLICTS OF INTEREST

Based on the article's results, the University of Helsinki has filed a patent application on the improved CRISPRa + ME reprogramming method to the National Board of Patents and Registration of Finland (patent application no. 20215848).

ACKNOWLEDGMENTS

The LCLs used for the research were obtained from THL Biobank (BB2016_56 and BB2018_33). We thank all of the sample donors for their generous participation in biobank research. We thank J. Ustinov, H. Grym, A. Laitinen, S. Eurola, and H. Ibrahim for technical assistance with the work and M. Bespalov and T. Barsby for comments on the manuscript. We also thank the Biocenter Finland Biomedicum Stem Cell Center core facility for technical support. Some of the computations were performed on resources provided by SNIC through the Uppsala Multidisciplinary Center for Advanced Computational Science (UPPMAX) under Project SNIC 2017/7-317. This work has been supported by the Jane and Aatos Erkko Foundation, the Academy of Finland (nos. 297466 and 312437, Center of Excellence in Stem Cell Metabolism), the Päivikki and Sakari Sohlberg Foundation, the Sigrid Jusélius Foundation, the Swedish Research Council, the Scandinavia–Japan Sasakawa Foundation, the Japan Eye Bank Association, the Astellas Foundation for Research on Metabolic Disorders and the Japan Society for the Promotion of Science (JSPS) Overseas Research Fellowships. The graphical abstract was created with BioRender.com.

Received: August 16, 2021
Revised: December 21, 2021
Accepted: December 21, 2021
Published: January 20, 2022

REFERENCES

Anokye-Danso, F., Trivedi, C.M., Juhr, D., Gupta, M., Cui, Z., Tian, Y., Zhang, Y., Yang, W., Gruber, P.J., Epstein, J.A., et al. (2011). Highly efficient miRNA-mediated reprogramming of mouse and human somatic cells to pluripotency. Cell Stem Cell *8*, 376–388. https://doi.org/10.1016/j.stem.2011.03.001.

Aran, D., Looney, A.P., Liu, L., Wu, E., Fong, V., Hsu, A., Chak, S., Naikawadi, R.P., Wolters, P.J., Abate, A.R., et al. (2019). Reference-based analysis of lung single-cell sequencing reveals a transitional profibrotic macrophage. Nat. Immunol. *20*, 163–172. https://doi.org/10.1038/s41590-018-0276-y.

Barrett, R., Ornelas, L., Yeager, N., Mandefro, B., Sahabian, A., Lenaeus, L., Targan, S.R., Svendsen, C.N., and Sareen, D. (2014). Reliable generation of induced pluripotent stem cells from human lymphoblastoid cell lines. Stem Cells Transl. Med. *3*, 1429–1434. https://doi.org/10.5966/sctm.2014-0121.

Bikard, D., Jiang, W., Samai, P., Hochschild, A., Zhang, F., and Marraffini, L.A. (2013). Programmable repression and activation of bacterial gene expression using an engineered CRISPR-Cas system. Nucleic Acids Res. *41*, 7429–7437. https://doi.org/10.1093/nar/gkt520.

Boegel, S., Löwer, M., Bukur, T., Sorn, P., Castle, J.C., and Sahin, U. (2018). HLA and proteasome expression body map. BMC Med. Genomics *11*, 1–12. https://doi.org/10.1186/s12920-018-0354-x.

Cacchiarelli, D., Trapnell, C., Ziller, M.J., Soumillon, M., Cesana, M., Karnik, R., Donaghey, J., Smith, Z.D., Zhang, X., Sui, S.J.H., et al. (2015). Integrative analyses of human reprogramming reveal dynamic nature of induced pluripotency. Cell *162*, 412–424. https://doi.org/10.1016/j.cell.2015.06.016.

Carcamo-Orive, I., Hoffman, G.E., Cundiff, P., Beckmann, N.D., Souza, S.D., Knowles, J.W., Patel, A., Papatsenko, D., Reaven, G.M., Whalen, S., et al. (2017). Analysis of transcriptional

variability in a large human iPSC library reveals genetic and non-genetic determinants of heterogeneity. Cell Stem Cell *20*, 518–532. https://doi.org/10.1016/j.stem.2016.11.005.

Collier, A.J., Panula, S.P., Schell, J.P., Douagi, I., Lanner, F., and Rugg-Gunn, P.J. (2017). Comprehensive cell surface protein profiling identifies specific markers of human naive and primed pluripotent states. Cell Stem Cell *20*, 874–890.e7. https://doi.org/10.1016/j.stem.2017.02.014.

Francesconi, M., Di Stefano, B., Berenguer, C., Andrés-Aguayo, L. De, Plana-Carmona, M., Mendez-Lago, M., Guillaumet-Adkins, A., Rodriguez-Esteban, G., Gut, M., Gut, I.G., et al. (2019). Single cell RNA-seq identifies the origins of heterogeneity in efficient cell transdifferentiation and reprogramming. Elife *8*, 1–22. https://doi.org/10.7554/eLife.41627.

Fuchs, E., and Weber, K. (1994). Intermediate filaments: structure, dynamics, function, and disease. Annu. Rev. Biochem. *63*, 345–382. https://doi.org/10.1146/annurev.bi.63.070194.002021.

Fujimori, K., Tezuka, T., Ishiura, H., Mitsui, J., Doi, K., Yoshimura, J., Tada, H., Matsumoto, T., Isoda, M., Hashimoto, R., et al. (2016). Modeling neurological diseases with induced pluripotent cells reprogrammed from immortalized lymphoblastoid cell lines. Mol. Brain *9*, 1–14. https://doi.org/10.1186/s13041-016-0267-6.

Fusaki, N., Ban, H., Nishiyama, A., Saeki, K., and Hasegawa, M. (2009). Efficient induction of transgene-free human pluripotent stem cells using a vector based on Sendai virus, an RNA virus that does not integrate into the host genome. Proc. Jpn. Acad. Ser. B Phys. Biol. Sci. *85*, 348–362. https://doi.org/10.2183/pjab.85.348.

Gussow, D., Rein, R., Ginjaar, I., Hochstenbach, F., Seeman, G., Kottman, A., and Ploegh, H.L. (1987). The human beta 2-microglobulin gene. Primary structure and definition of the transcriptional unit. J. Immunol. *139*, 3132–3138.

Hale, G., Xia, M., Tighe, H.P., Dyer, M.J.S., and Waldmann, H. (1990). The CAMPATH-1 antigen (CDw52). Tissue Antigens *35*, 118–127. https://doi.org/10.1111/j.1399-0039.1990.tb01767.x.

Houbaviy, H.B., Murray, M.F., and Sharp, P.A. (2003). Embryonic stem cell-specific MicroRNAs. Dev. Cell *5*, 351–358. https://doi.org/10.1016/S1534-5807(03)00227-2.

Huang, H.P., Chen, P.H., Yu, C.Y., Chuang, C.Y., Stone, L., Hsiao, W.C., Li, C.L., Tsai, S.C., Chen, K.Y., Chen, H.F., et al. (2011). Epithelial cell adhesion molecule (EpCAM) complex proteins promote transcription factor-mediated pluripotency reprogramming. J. Biol. Chem. *286*, 33520–33532. https://doi.org/10.1074/jbc.M111.256164.

Hussein, S.M.I., Puri, M.C., Tonge, P.D., Benevento, M., Corso, A.J., Clancy, J.L., Mosbergen, R., Li, M., Lee, D.-S., Cloonan, N., et al. (2014). Genome-wide characterization of the routes to pluripotency. Nature *516*, 198–206. https://doi.org/10.1038/nature14046.

Kaisers, W., Schwender, H., and Schaal, H. (2017). Sample size estimation for detection of splicing events in transcriptome sequencing data. Int. J. Mol. Sci. *18*, 1–17. https://doi.org/10.3390/ijms18091900.

Kilpinen, H., Goncalves, A., Leha, A., Afzal, V., Alasoo, K., Ashford, S., Bala, S., Bensaddek, D., Casale, F.P., Oliver, J., et al. (2017). Common genetic variation drives molecular heterogeneity in human iPSCs. Nature *546*, 370–375. https://doi.org/10.1038/nature22403.

Kogut, I., McCarthy, S.M., Pavlova, M., Astling, D.P., Chen, X., Jakimenko, A., Jones, K.L., Getahun, A., Cambier, J.C., Pasmooij, A.M.G., et al. (2018). High-efficiency RNA-based reprogramming of human primary fibroblasts. Nat. Commun. *9*, 1–15. https://doi.org/10.1038/s41467-018-03190-3.

Lim, H.Y.G., Alvarez, Y.D., Gasnier, M., Wang, Y., Tetlak, P., Bissiere, S., Wang, H., Biro, M., and Plachta, N. (2020). Keratins are asymmetrically inherited fate determinants in the mammalian embryo. Nature *585*, 404–409. https://doi.org/10.1038/s41586-020-2647-4.

Liu, X., Ouyang, J.F., Rossello, F.J., Tan, J.P., Knaupp, A.S., Sun, G., Chy, H.S., Huang, Z., Pflueger, J., Firas, J., et al. (2020). Reprogramming roadmap reveals route to human induced trophoblast stem cells. Nature *586*, 101–107. https://doi.org/10.1038/s41586-020-2734-6.

Mabbott, N.A., Baillie, J.K., Brown, H., Freeman, T.C., and Hume, D.A. (2013). An expression atlas of human primary cells: inference of gene function from coexpression networks. BMC Genomics *14*, 1–13. https://doi.org/10.1186/1471-2164-14-632.

Miyoshi, N., Ishii, H., Nagano, H., Haraguchi, N., Dewi, D.L., Kano, Y., Nishikawa, S., Tanemura, M., Mimori, K., Tanaka, F., et al. (2011). Reprogramming of mouse and human cells to pluripotency using mature microRNAs. Cell Stem Cell *8*, 633–638. https://doi.org/10.1016/j.stem.2011.05.001.

Mong, E.F., Yang, Y., Akat, K.M., Canfield, J., VanWye, J., Lockhart, J., Tsibris, J.C.M., Schatz, F., Lockwood, C.J., Tuschl, T., et al. (2020). Chromosome 19 microRNA cluster enhances cell reprogramming by inhibiting epithelial-to-mesenchymal transition. Sci. Rep. *10*, 1–13. https://doi.org/10.1038/s41598-020-59812-8.

Neitzel, H. (1986). A routine method for the establishment of permanent growing lymphoblastoid cell lines. Hum. Genet. *73*, 320–326. https://doi.org/10.1007/BF00279094.

Okita, K., Matsumura, Y., Sato, Y., Okada, A., Morizane, A., Okamoto, S., Hong, H., Nakagawa, M., Tanabe, K., Tezuka, K., et al. (2011). A more efficient method to generate integration-free human iPS cells. Nat. Methods *8*, 409–412. https://doi.org/10.1038/nmeth.1591.

Ozgyin, L., Horvath, A., Hevessy, Z., and Balint, B.L. (2019). Extensive epigenetic and transcriptomic variability between genetically identical human B-lymphoblastoid cells with implications in pharmacogenomics research. Sci. Rep. *9*, 1–16. https://doi.org/10.1038/s41598-019-40897-9.

Papatheodorou, I., Moreno, P., Manning, J., Fuentes, A.M.P., George, N., Fexova, S., Fonseca, N.A., Füllgrabe, A., Green, M., Huang, N., et al. (2020). Expression Atlas update: from tissues to single cells. Nucleic Acids Res. *48*, D77–D83. https://doi.org/10.1093/nar/gkz947.

Pera, M.F., Reubinoff, B., and Trounson, A. (2000). Human embryonic stem cells. J. Cell Sci. *113*, 5–10.

Ploegh, H.L., Orr, H.T., and Strominger, J.L. (1981). Major histocompatibility antigens: the human (HLA-A,-B,-C) and murine (H-

2K, H-2D) class I molecules. Cell *24*, 287–299. https://doi.org/10.1016/0092-8674(81)90318-4.

Rajesh, D., Dickerson, S.J., Yu, J., Brown, M.E., Thomson, J.A., and Seay, N.J. (2011). Human lymphoblastoid B-cell lines reprogrammed to EBV-free induced pluripotent stem cells. Blood *118*, 1797–1800. https://doi.org/10.1182/blood-2011-01-332064.

Schiebinger, G., Shu, J., Tabaka, M., Cleary, B., Subramanian, V., Solomon, A., Gould, J., Liu, S., Lin, S., Berube, P., et al. (2019). Optimal-transport analysis of single-cell gene expression identifies developmental trajectories in reprogramming. Cell *176*, 928–943. https://doi.org/10.1016/j.cell.2019.01.006.

Sie, L., Loong, S., and Tan, E.K. (2009). Utility of lymphoblastoid cell lines. J. Neurosci. Res. *87*, 1953–1959. https://doi.org/10.1002/jnr.22000.

Subramanyam, D., Lamouille, S., Judson, R.L., Liu, J.Y., Bucay, N., Derynck, R., and Blelloch, R. (2011). Multiple targets of miR-302 and miR-372 promote reprogramming of human fibroblasts to induced pluripotent stem cells. Nat. Biotechnol. *29*, 443–448. https://doi.org/10.1038/nbt.1862.

Takahashi, K., and Yamanaka, S. (2016). A decade of transcription factor-mediated reprogramming to pluripotency. Nat. Rev. Mol. Cell Biol. *17*, 183–193. https://doi.org/10.1038/nrm.2016.8.

Takahashi, K., Tanabe, K., Ohnuki, M., Narita, M., Sasaki, A., Yamamoto, M., Nakamura, M., Sutou, K., Osafune, K., and Yamanaka, S. (2014). Induction of pluripotency in human somatic cells via a transient state resembling primitive streak-like mesendoderm. Nat. Commun. *5*, 1–9. https://doi.org/10.1038/ncomms4678.

Thomas, S.M., Kagan, C., Pavlovic, B.J., Burnett, J., Patterson, K., Pritchard, J.K., and Gilad, Y. (2015). Reprogramming LCLs to iPSCs results in recovery of donor-specific gene expression signature. PLoS Genet. *11*, 1–17. https://doi.org/10.1371/journal.pgen.1005216.

Thul, P.J., Akesson, L., Wiking, M., Mahdessian, D., Geladaki, A., Ait Blal, H., Alm, T., Asplund, A., Björk, L., Breckels, L.M., et al. (2017). A subcellular map of the human proteome. Science *356*, 1–12. https://doi.org/10.1126/science.aal3321.

Töhönen, V., Katayama, S., Vesterlund, L., Jouhilahti, E.-M., Sheikhi, M., Madissoon, E., Filippini-Cattaneo, G., Jaconi, M., Johnsson, A., Burglin, T.R., et al. (2015). Novel PRD-like homeodomain transcription factors and retrotransposon elements in early human development. Nat. Commun. *6*, 1–9. https://doi.org/10.1038/ncomms9207.

Tran, K.A., Pietrzak, S.J., Zaida, N.Z., Siahpirani, A.F., McCalla, S.G., Zhou, A.S., Iyer, G., Roy, S., and Sridharan, R. (2019). Defining reprogramming checkpoints from single-cell analyses of induced pluripotency. Cell Rep. *27*, 1726–1741. https://doi.org/10.1016/j.celrep.2019.04.056.

Trokovic, R., Weltner, J., Noisa, P., Raivio, T., and Otonkoski, T. (2015). Combined negative effect of donor age and time in culture on the reprogramming efficiency into induced pluripotent stem cells. Stem Cell Res. *15*, 254–262. https://doi.org/10.1016/j.scr.2015.06.001.

Ustianenko, D., Chiu, H.-S.C., Treiber, T., Weyn-Vanhentenryck, S.M., Treiber, N., Meister, G., Sumazin, P., and Zhang, C. (2018). LIN28 selectively modulates a subclass of let-7 microRNAs. Mol. Cell *71*, 271–283. https://doi.org/10.1016/j.molcel.2018.06.029.

Velychko, S., Adachi, K., Kim, K.P., Hou, Y., MacCarthy, C.M., Wu, G., and Schöler, H.R. (2019). Excluding Oct4 from Yamanaka cocktail unleashes the developmental potential of iPSCs. Cell Stem Cell *25*, 737–753.e4. https://doi.org/10.1016/j.stem.2019.10.002.

Wang, Y., Zhao, C., Hou, Z., Yang, Y., Bi, Y., Wang, H., Zhang, Y., and Gao, S. (2018). Unique molecular events during reprogramming of human somatic cells to induced pluripotent stem cells (iPSCs) at naïve state. Elife *7*, 1–21. https://doi.org/10.7554/eLife.29518.

Warren, L., Manos, P.D., Ahfeldt, T., Loh, Y., Li, H., Daley, Q., Brack, A.S., Collins, J.J., Cowan, C., Thorsten, M., et al. (2010). Highly efficient reprogramming to pluripotency and directed differentiation of human cells using synthetic modified mRNA. Cell Stem Cell *7*, 618–630. https://doi.org/10.1016/j.stem.2010.08.012.

Weltner, J., and Trokovic, R. (2021). Reprogramming of fibroblasts to human iPSCs by CRISPR activators. In Nuclear Reprogramming: Methods and Protocols, K. Hu, ed. (Springer US), pp. 175–198. https://doi.org/10.1007/978-1-0716-1084-8_12.

Weltner, J., Balboa, D., Katayama, S., Bespalov, M., Krjutškov, K., Jouhilahti, E.-M., Trokovic, R., Kere, J., and Otonkoski, T. (2018). Human pluripotent reprogramming with CRISPR activators. Nat. Commun. *9*, 1–12. https://doi.org/10.1038/s41467-018-05067-x.

Worringer, K.A., Rand, T.A., Hayashi, Y., Sami, S., Takahashi, K., Tanabe, K., Narita, M., Srivastava, D., and Yamanaka, S. (2014). The let-7/LIN-41 pathway regulates reprogramming to human induced pluripotent stem cells by controlling expression of pro-differentiation genes. Cell Stem Cell *14*, 40–52. https://doi.org/10.1016/j.stem.2013.11.001.

Stem Cell Reports, Volume *17*

Supplemental Information

CRISPR activation enables high-fidelity reprogramming into human pluripotent stem cells

Joonas Sokka, Masahito Yoshihara, Jouni Kvist, Laura Laiho, Andrew Warren, Christian Stadelmann, Eeva-Mari Jouhilahti, Helena Kilpinen, Diego Balboa, Shintaro Katayama, Aija Kyttälä, Juha Kere, Timo Otonkoski, Jere Weltner, and Ras Trokovic

Supplemental information

Supplemental Figures

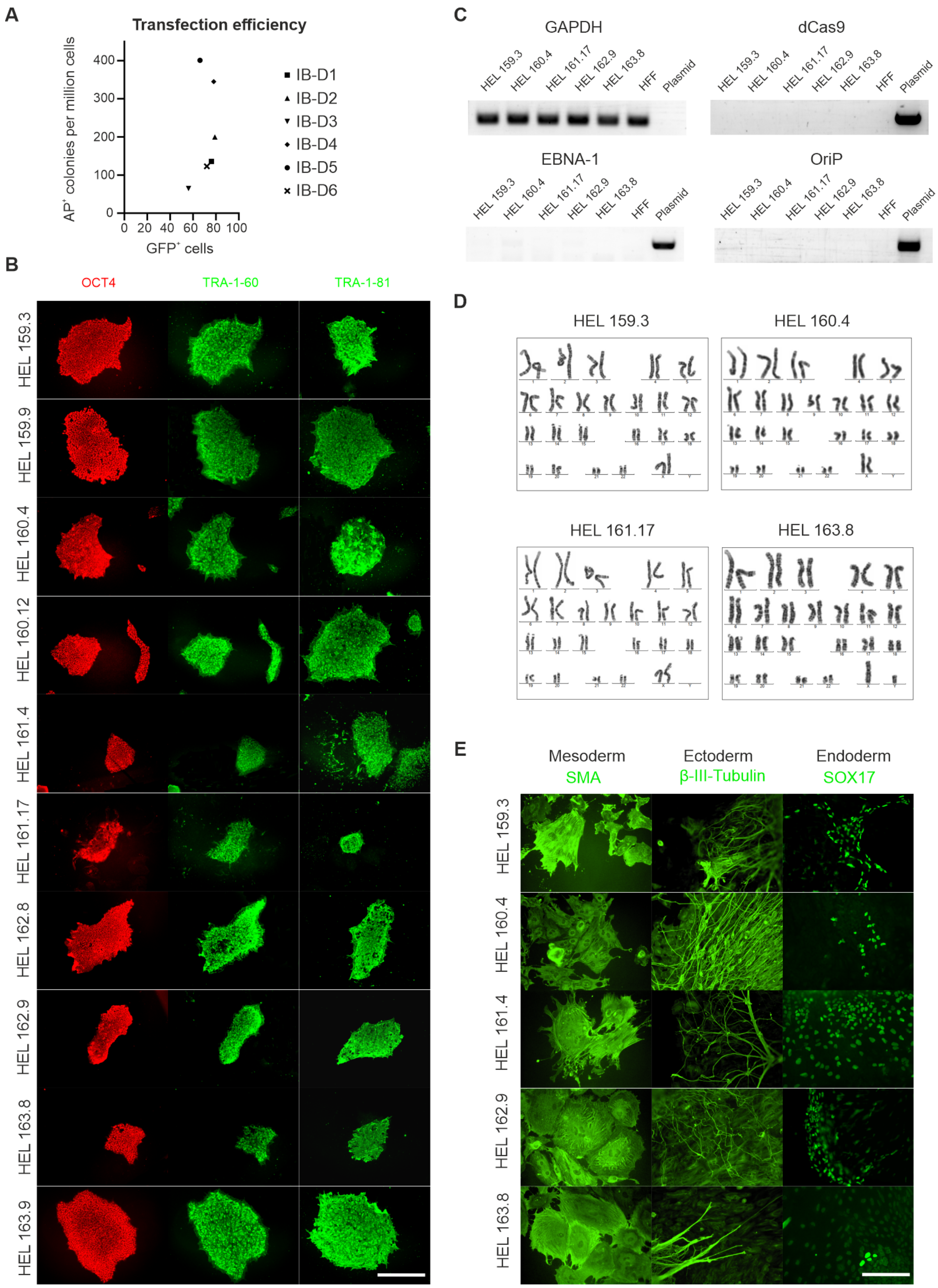

Figure S1: Validation of CRISPRa-iPSC lines, related to Figure 1
A) Transfection efficiency of six different LCL reprogrammed with the conventional transgenic method in relation to the amount of generated colonies.
B) Immunocytochemistry images showing OCT4, TRA-1-60 and TRA-1-81 stainings in generated CRISPRa-iPSC lines. Scale bar= 400 µm
C) Genomic DNA PCR gel images showing the removal of episomal plasmids from generated CRISPRa-iPSC lines. GAPDH= gDNA control, HFF= fibroblast control, Plasmid= dCas9-activator plasmid control
D) Karyotyping results of iPSC lines generated from LCL donors IB-D1, IB-D2, IB-5 and IB-D6
E) EB-assay with stainings for endodermal (SOX17), ectodermal (β-III-tubulin) and mesodermal (Smooth muscle actin, SMA) germ lineage derivatives. Scale bar= 200 µm

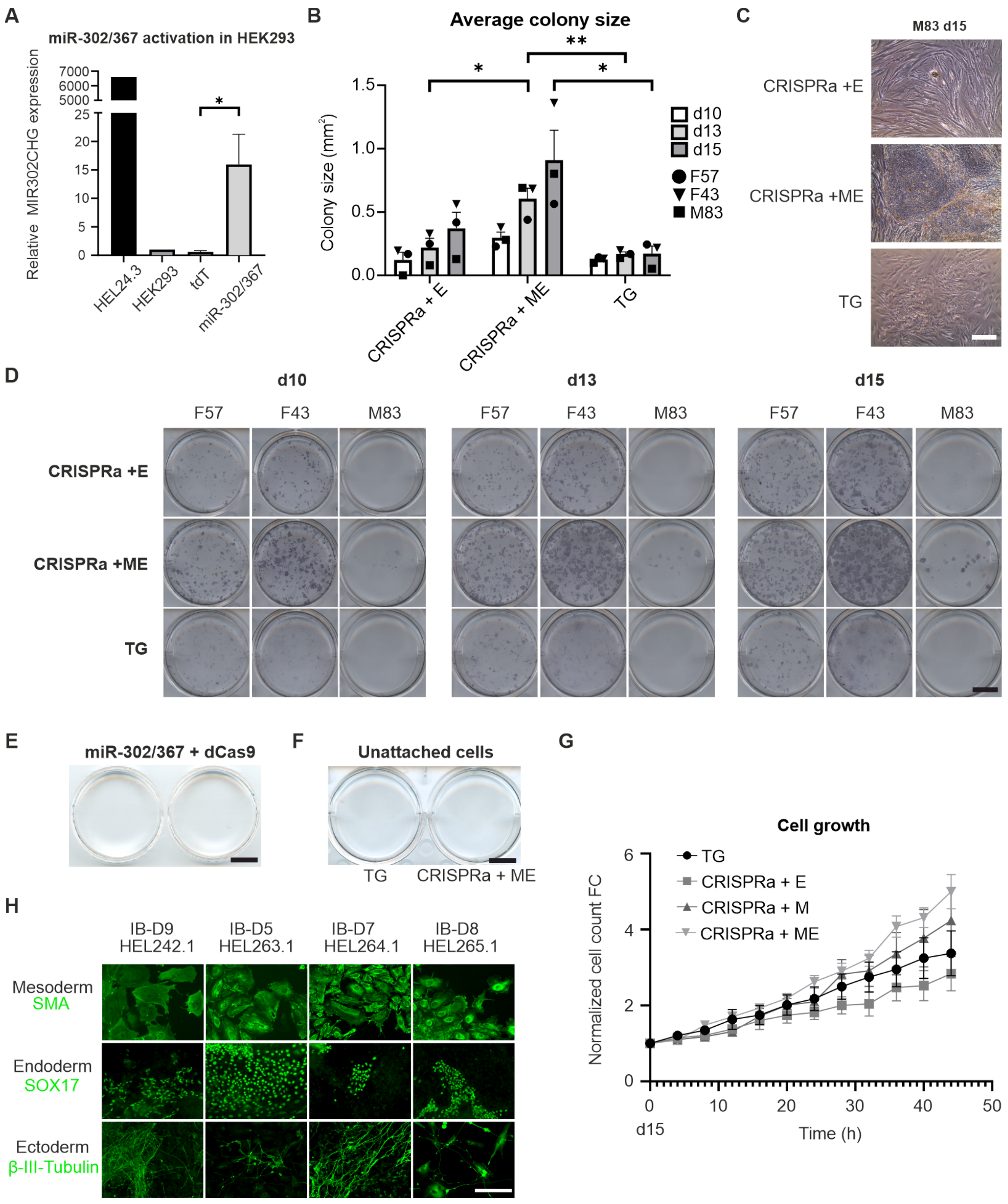

A
miR-302/367 activation in HEK293
Relative MIR302CHG expression
7000
6000
5000
25
20
15
10
5
0
*
HEL24.3
HEK293
tdT
miR-302/367
B
Average colony size
Colony size (mm²)
1.5
1.0
0.5
0.0
*
**
*
d10
d13
d15
F57
F43
M83
CRISPRa + E
CRISPRa + ME
TG
C
M83 d15
CRISPRa +E
CRISPRa +ME
TG
D
d10
d13
d15
F57
F43
M83
CRISPRa +E
CRISPRa +ME
TG
E
miR-302/367 + dCas9
F
Unattached cells
TG
CRISPRa + ME
G
Cell growth
TG
CRISPRa + E
CRISPRa + M
CRISPRa + ME
Normalized cell count FC
6
4
2
0
0
10
20
30
40
50
d15
Time (h)
H
IB-D9
HEL242.1
IB-D5
HEL263.1
IB-D7
HEL264.1
IB-D8
HEL265.1
Mesoderm
SMA
Endoderm
SOX17
Ectoderm
β-III-Tubulin

Figure S2: Additional induction data, related to Figure 2

A) Validation of CRISPR activation of miR-302/367 cluster in HEK293 cells with and without miR-302/367 gRNA targeting. n=5 independent experiments, Error bars SEM, Student's t-test *=$p<0.05$. TdTomato guide was used as a negative control, and iPSC line (HEL24.3) was used as a positive control.

B) Measurement of iPSC-like colony size at days 10, 13 and 15 using three different primary fibroblast lines and reprogramming methods. n=3, each point represents an independent assay where cells were counted on either day 10, 13 or 15 as indicated by the legend, Error bars=SEM, p-values were calculated with repeated measures one-way ANOVA, *=$p<0.05$, **=$p<0.01$

C) Bright field images of M83 iPSC colonies on reprogramming day 15 using three different reprogramming conditions. Scale bar= 400 µm

D) AP-stainings of primary fibroblast lines F57, F43 and M83 on reprogramming days 10, 13 and 15 using three different reprogramming methods. Scale bar= 1 cm

E) AP-staining of LCL reprogrammed with dCas9 activator and miR-302/367 targeting guides only. Scale bar= 1 cm

F) AP-staining of re-plated unattached day 10 cells from TG and CRISPRa + ME inductions. Scale bar= 1 cm

G) iPSC-like cells growth in TG, CRISPRa + E, CRISPRa + M and CRISPRa + ME conditions from reprogramming day 15 to day 17. Cell count fold change is normalized to the day 15 starting point. n=6 independent experiments, Error bars=SEM

H) Multilineage differentiation of four different LCL donor lines reprogrammed by CRISPRa + ME shown by immunostaining for endodermal (SOX17), ectodermal (β-III-tubulin) and mesodermal (Smooth muscle actin, SMA) germ layer derivatives. Scale bar= 200 µm

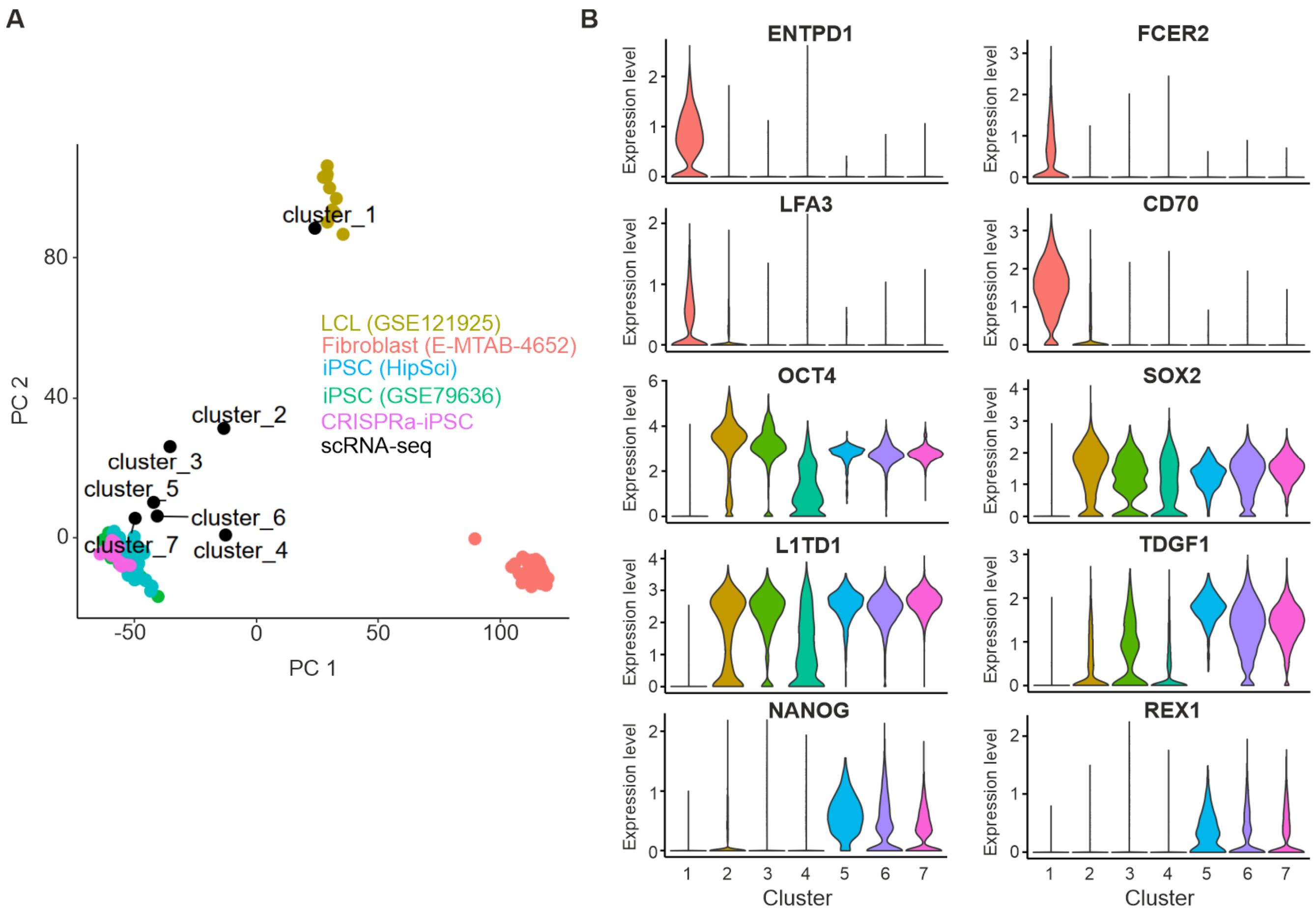

Figure S3: scRNA-seq gene expression, related to Figure 3
A) PCA of combined bulk RNA-seq data and scRNA-seq clusters with reference iPSC (HipSci, GSE79636), LCL (GSE121925) and fibroblast (E-MTAB-4652) cell lines.
B) Violin plots showing expression levels of selected LCL (ENTPD1, FCER2, LFA3, CD70) and undifferentiated hESC (OCT4, SOX2, L1TD1, TDGF1, NANOG, REX1) associated markers across the clusters.

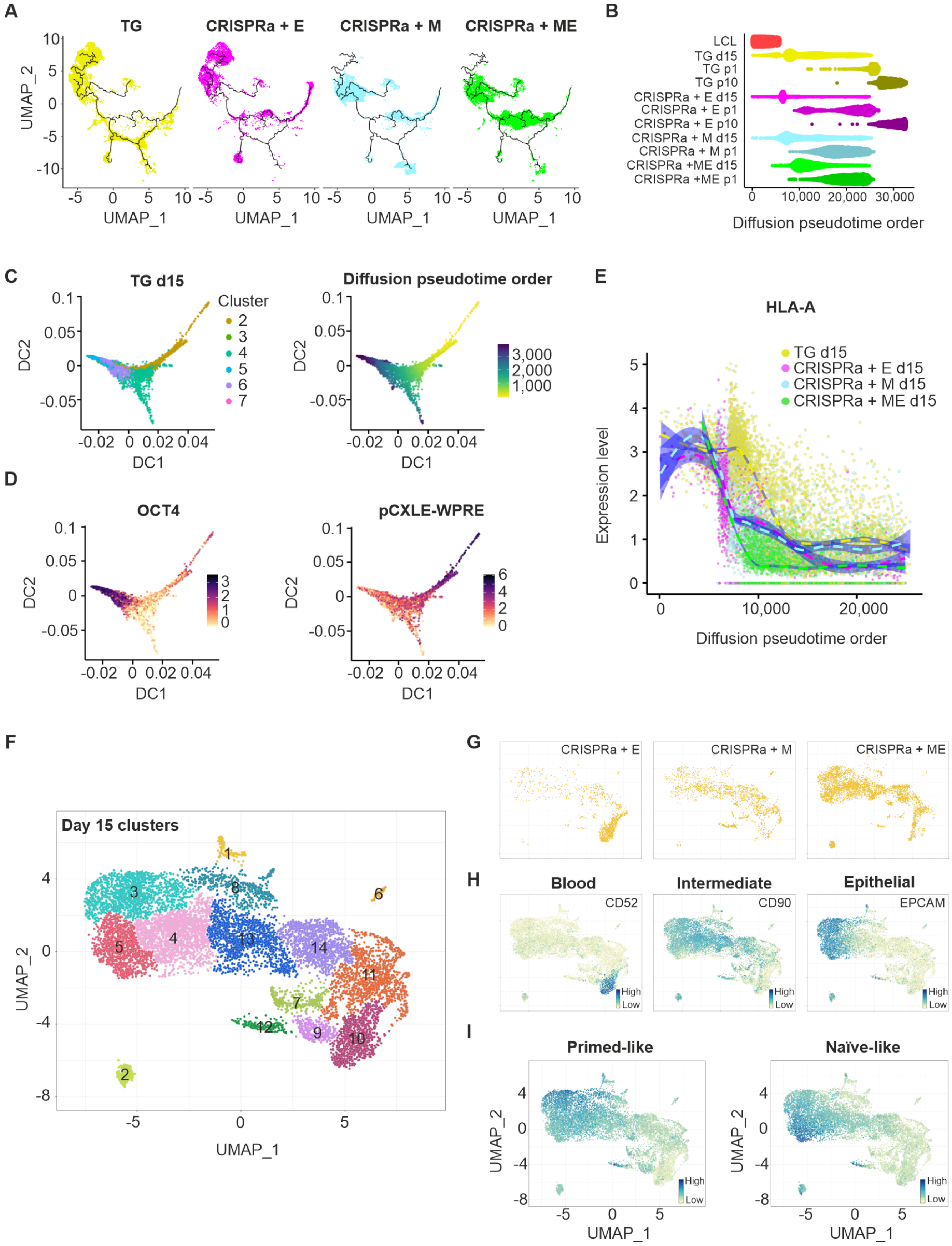
A
TG
CRISPRa + E
CRISPRa + M
CRISPRa + ME
UMAP_2
UMAP_1
B
LCL
TG d15
TG p1
TG p10
CRISPRa + E d15
CRISPRa + E p1
CRISPRa + E p10
CRISPRa + M d15
CRISPRa + M p1
CRISPRa +ME d15
CRISPRa +ME p1
Diffusion pseudotime order
C
TG d15
Cluster
DC1
DC2
Diffusion pseudotime order
D
OCT4
pCXLE-WPRE
E
HLA-A
TG d15
CRISPRa + E d15
CRISPRa + M d15
CRISPRa + ME d15
Expression level
Diffusion pseudotime order
F
Day 15 clusters
UMAP_1
UMAP_2
G
CRISPRa + E
CRISPRa + M
CRISPRa + ME
H
Blood
Intermediate
Epithelial
CD52
CD90
EPCAM
High
Low
I
Primed-like
Naïve-like

Figure S4: Additional pseudotime and diffusion map analyses, related to Figures 3 and 4

A) Pseudotime analysis showing the trajectory of the cells from each reprogramming condition during reprogramming with cluster 1 excluded.

B) Diffusion pseudotime analysis showing the predicted order of the cells from all samples based on their expression profiles

C) Diffusion component analysis of mid-reprogramming TG cells excluding LCL cluster 1.

D) Expression of OCT4 and pCXLE-WPRE on the diffusion map.

E) Diffusion pseudotime combined with the expression level of HLA-A in the mid-reprogramming samples. Dashed lines and blue shades are fitted generalized additive model with 95% confidence interval in each condition. Each color represents the different reprogramming condition.

F) UMAP clustering of day 15 samples only

G) Distribution of different CRISPRa condition cells in d15 only clusters

H) Expression of blood cell marker CD52, intermediate reprogramming marker CD90, and epithelial marker EPCAM in d15 only clusters

I) Expression of primed and naïve markers in d15 only clusters. Genes are listed in supplemental table S6.

Supplemental Tables

Table S1: Reprogramming plasmids, related to Figures 1 and 2

Plasmid	Method	Addgene number
pCXLE-hOCT3/4-shp53-F	Conventional	27077
pCXLE-hSK	Conventional	27078
pCXLE-hUL	Conventional	27080
pCXLE-dCas9VP192-T2A-EGFP-shP53	CRISPRa	69535
GG-EBNA-O3S2K2M2L1-PP	CRISPRa	102902
GG-EBNA-EEA-guide1-PGK-Puro	CRISPRa	102904
GG-EBNA-EEA-5guides-PGK-Puro	CRISPRa	102898
GG-EBNA-R2N2-PGK-Puro	CRISPRa	-
GG-EBNA-R2N2-EEAg1-PGK-Puro	CRISPRa	-
GG-EBNA-R2N2-EEAg5-PGK-Puro	CRISPRa	-
GG-EBNA-N5L5-EEAg1-PGK-Puro	CRISPRa	-
GG-EBNA-MIR302g7-PGK-Puro	CRISPRa	-
pCXLE-EGFP	Control	27082

Table S2: Bulk RNA-seq samples, related to Figure 1

iPSC line	Cell origin	Donor line	Sex	Reprogramming method
HEL159.3	LCL	IB-D6	F	CRISPRa
HEL159.9	LCL	IB-D6	F	CRISPRa
HEL160.4	LCL	IB-D2	F	CRISPRa
HEL160.12	LCL	IB-D2	F	CRISPRa
HEL161.4	LCL	IB-D1	F	CRISPRa
HEL161.17	LCL	IB-D1	F	CRISPRa
HEL162.8	LCL	IB-D4	F	CRISPRa
HEL162.9	LCL	IB-D4	F	CRISPRa
HEL163.8	LCL	IB-D5	M	CRISPRa
HEL163.9	LCL	IB-D5	M	CRISPRa
HEL139.2	Fibroblast	F72	F	CRISPRa
HEL139.5	Fibroblast	F72	F	CRISPRa
HEL139.8	Fibroblast	F72	F	CRISPRa
HEL140.1	Fibroblast	HFF	M	CRISPRa
HEL141	Fibroblast	HFF	M	CRISPRa

Table S3: Primers and antibodies, Related to Figures 1, 2 and 5

Gene	Forward primer	Reverse primer	Product length (bp)
OriP	TTC CAC GAG GGT AGT GAA CC	TCG GGG GTG TTA GAG ACA AC	544
EBNA-1	ATC GTC AAA GCT GCA CAC AG	CCC AGG AGT CCC AGT AGT CA	666
dCas9	AAA CAG CAG ATT CGC CTG GA	CTG TCT GCA CCT CGG TCT TT	1934
GAPDH	AAG AAG GTG GTG AAG CAG GC	CAG GAA ATG AGC TTG ACA AAG	164
OCT4 (POU5F1)	TTG GGC TCG AGA AGG ATG TG	GTG AAG TGA GGG CTC CCA TA	193
SOX2	GCC CTG CAG TAC AAC TCC AT	TGC CCT GCT GCG AGT AGG A	85
NANOG	CTC AGC CTC CAG CAG ATG C	TAG ATT TCA TTC TCT GGT TCT GG	94
REX1 (ZFP42)	CGT TTC GTG TGT CCC TTT CAA	CCT CTT GTT CAT TCT TGT TCG T	106
LIN28A	AGG AGA CAG GTG CTA CAA CTG	TCT TGG GCT GGG GTG GCA G	74
TDGF1	TCA GAG ATG ACA GCA TTT GGC	TTC AGG CAG CAG GTT CTG TTT A	118
KLF4	CCG CTC CAT TAC CAA G	CAC GAT CGT CTT CCC CTC TT	80
cMYC	AGC GAC TCT GAG GAG GAA CA	CTC TGA CCT TTT GCC AGG AG	87
pri-miR-302/367 (MIR302CHG) intronic region	TAA CTT TAT TGT ATT GAC CGC AGC TC	GTC ACA GCA AGT GCC TCC AT	106
pri-miR-302/367 (MIR302CHG) exonic region	TGG AGG AGA ACA CGA ATC TTTG GG	ACA AGC AGC AAA AGC AAT TGA GGT A	127/74 splice variants
Cyclophilin G	CAA TGG CCA ACA GAG GGA AG	CCA AAA ACA ACA TGA TGC CCA	94
**Primary Antibody**	**Manufacturer**	**Host Species and Dilution**	**Catalog**
OCT3/4	Santa Cruz Biotechnology	Rabbit 1:500	SC-9081
NANOG	Cell signaling technologies	Rabbit 1:200	4903S
TRA-1-60	Invitrogen	Mouse 1:500	MA1-023

TRA-1-81	Invitrogen	Mouse 1:250	MA1-024
SOX17	R&D Systems	Goat 1:500	AF1924
α-Smooth muscle actin	Sigma	Mouse 1:500	A2547
β-III-tubulin	Abcam	Rabbit 1:500	Ab18207
**Secondary Antibody**	**Manufacturer**	**Host Species and Dilution**	**Catalog**
Anti-Rabbit IgG (H+L) Alexa Fluor 594 (Red)	Invitrogen	Donkey 1:500	A21207
Anti-Rabbit IgG (H+L) Alexa Fluor 488 (Green)	Invitrogen	Donkey 1:500	A21206
Anti-Mouse IgG (H+L) Alexa Fluor 594 (Red)	Invitrogen	Donkey 1:500	A21203
Anti-Mouse IgG (H+L) Alexa Fluor 488 (Green)	Invitrogen	Donkey 1:500	A21202
Anti-Goat IgG (H+L) Alexa Fluor 594 (Red)	Invitrogen	Donkey 1:500	A11058
Anti-Goat IgG (H+L) Alexa Fluor 488 (Green)	Invitrogen	Donkey 1:500	A11055

Table S4: Guide RNA oligos, related to Figure 2

Gene	Guide	Sequence
miR-302/367	1	GTGGAAAGGACGAAACACCgAAGAATAGTATAAATAGAAGgttttagagctaGAAAtag
miR-302/367	2	GTGGAAAGGACGAAACACCgATCTCAGAGAATCATTACAAgttttagagctaGAAAtag
miR-302/367	3	GTGGAAAGGACGAAACACCgAGGGAATGTATGATCCTGGGgttttagagctaGAAAtag
miR-302/367	4	GTGGAAAGGACGAAACACCgAAAAGGATCCAGACCCACCCgttttagagctaGAAAtag
miR-302/367	5	GTGGAAAGGACGAAACACCgTTTAAGAGGAAGATATCTTGgttttagagctaGAAAtag
miR-302/367	6	GTGGAAAGGACGAAACACCgATGCCATCAAACAAGCAGATgttttagagctaGAAAtag
miR-302/367	7	GTGGAAAGGACGAAACACCgCAATGCCTTTCTCGGCTCAGgttttagagctaGAAAtag

Table S5: scRNA-seq cluster cell counts, related to Figure 3

Cluster	LCL	TG d15	TG p1	TG p10	CRISPRa + E d15	CRISPRa + E p1	CRISPRa + E p10	CRISPRa + M d15	CRISPRa + M p1	CRISPRa + ME d15	CRISPRa + ME p1
**1**	5315	371	0	0	92	0	0	75	1	19	1
**2**	15	852	32	30	966	300	31	1109	65	2052	368
**3**	67	36	324	200	93	250	185	98	30	221	33
**4**	18	1073	152	244	69	63	264	249	1373	138	302
**5**	0	465	0	0	0	0	0	0	0	4	0
**6**	0	1438	448	860	71	150	905	108	800	956	679
**7**	0	63	834	1869	26	133	1665	116	2220	557	1215

Table S6: hESC, primed and naïve associated gene sets, related to Figures 3 and 4

**hESC**	ABCG2, ALPP, ANO6, BCL3, BNIP3, BRIX1, BUB1, CCNA2, CD24, CD59, CD9, CD90, CDC42, CDH1, CDK1, CDK8, CHD1, CHD7, COMMD3, CRABP2, CTNNB1, CXCL5, DIAPH2, DNMT1, DNMT3B, DPPA2, DPPA3, DPPA4, DPPA5, EDNRB, EPCAM, ESRG, ESRRB, FBXO15, FGF2, FGF4, FGF5, FOXA2, FOXD3, FUT4, FZD1, GABRB3, GAL, GATA6, GBX2, GDF3, GJB1, GJB4, GJC1, GRB7, HDAC1, HES1, HESX1, HHEX, HMGA2, HOXB5, HSPA9, IDO1, IFITM2, IGF2BP2, IL6ST, ITGA4, ITGA6, ITGB1, KAT5, KCNIP3, KHDC3L, KIT, KITLG, KLF4, KLF5, KNL1, L1TD1, LCK, LEF1, LEFTY1, LEFTY2, LIFR, LIN28A, LMNA, MYBL2, MYC, NACC1, NANOG, NODAL, NOG, NR5A2, NR6A1, NUMB, OCT4, OTX2, PCGF2, PECAM1, PITX2, PIWIL1, PIWIL2, PIWIL4, PML, PODXL, PRDM14, PRDM5, PROM1, PTEN, PUM2, REST, REX1, RIF1, SALL4, SCN1A, SEMA3A, SFRP2, SMAD1, SMAD2, SMAD3, SMAD4, SMAD5, SMAD9, SOX15, SOX2, STAT3, SUMO2, TAF8, TBX3, TCF3, TCL1A, TDGF1, TERF1, TERT, TEX19, TFCP2L1, THAP11, TRIM22, TRIM28, TRIM6, UTF1, XIST, ZFX, ZIC1, ZSCAN10, ZSCAN4
**Primed**	NLGN4X, PTPRG, AC022140.1, LARGE1, JARID2, RIMS2, KCND2, NRXN1, TCF7L1, DLGAP1, TMEM132B, FOXO1, ADCY2, CADM2, GALNT17, TERF1, ANK3, FIRRE, AP002856.2, AC009446.1, SHISA9, AL590705.1, MGAT4C, AL365259.1, PTPRZ1, RYR2, LINC00678, DTNA, GPC6, ANOS1, PCDH11X, GPC4, LEFTY1
**Naïve**	CHODL, NLRP7, SLC16A10, UTF1, MT1G, AC011447.3, ZYG11A, CBFA2T2, MT1H, ZNF600, AKAP12, TRIML2, WDHD1, PRODH, RESF1, SERPINB9, NLRP2, NLRP1, ASRGL1, AC092546.1, LINC01950, PTCHD1, SLC25A16, DNMT3L, ZNF729, CNR2, PBX4, BRDT

Supplemental experimental procedures

Cell imaging

For light microscopy a Leica DM IL LED microscope (Leica-microsystems) was used, and pictures of live cells were taken with an attached Leica EC3 (Leica-microsystems) camera using LAS-EZ imaging software (Leica-microsystems). EVOS FL cell imaging system (Thermo Fisher) was used for fluorescence imaging. Whole culture plate images for the cell growth assays were taken with the IncuCyte S3 live-cell analysis system (Sartorius).

Guide RNA design and validation

Guide cassettes were designed and produced as previously described (Balboa et al., 2015; Weltner et al., 2018). Guide RNA (gRNA) expression cassettes, containing the U6 promoter, chimeric single gRNA and Pol III terminator, were assembled by PCR amplification and concatenated into GG-dest plasmid (Addgene #69538) using Golden Gate assembly. Concatenated guide sets were cloned from GG-dest into an episomal OriP-EBNA1 containing plasmid backbone for reprogramming experiments. A list of miR-302/367 targeting gRNA oligonucleotides is provided in supplemental table S4. Guides targeting pluripotency factors and the EEA-motif (Addgene #102902, #102904 and #102898) have been described previously (Weltner et al., 2018). To validate gRNAs, HEK293s were transfected using 4:1 ratio of FuGENE HD transfection reagent (Promega) with 500 ng of dCas9 transactivator encoding plasmid (Addgene plasmid #69535) and 100-200 ng of gRNA cassette. Transfected cells were cultured for 72 h, after which they were collected for quantitative reverse transcription PCR (qRT-PCR) analysis.

Alkaline phosphatase staining

iPSC colonies were fixed with 4% PFA for 10 min and washed with PBS. Cells were then stained with NBT/BCIP (Roche) containing buffer (0.1 M Tris HCL pH 9.5, 0.1M NaCl, 0.05 M $MgCl_2$) until purple precipitate formed. The reaction was stopped by washing the plates with PBS.

Embryoid body assay

For the embryoid body (EB) assay iPSC were passaged on ultra-low attachment 6-well culture plates in hES medium without bFGF. The medium was supplemented with 10 µM ROCKi Y-27632 for the first 24 h. The cells were cultured as EB aggregates for two weeks and the medium was changed every other day. After two weeks, the EBs were plated onto a 24 well-plate and cultured for 7 days in hES medium without bFGF. Thereafter, the EBs were fixed with 4% PFA for 30 min for immunocytochemistry.

Quantitative reverse transcription PCR

Total RNA was extracted from cells using NucleoSpin RNA Plus kit (Macherey-Nagel) and complementary DNA (cDNA) was synthesized from 1 µg of RNA by reverse transcription with 0.5 µl Moloney murine leukemia virus reverse transcriptase (Promega), 0.5 µl Riboblock RNAse inhibitor (Thermo Scientific), 0.2 µl Random hexamers (Promega), 1 µl Oligo (dT) 18 Primer (Thermo Scientific) and 2.5 ul dNTP (2.5 mM) at 37 °C for 90 minutes. For qRT-PCR reactions, 1 µl of cDNA was added to a master mix containing 4 µl of 5x HOT FIREPol EvaGreen qPCR Mix Plus (no ROX) (Solis Biodyne) and 10 µl of DEPC-H_2O per reaction. QIAgility (Qiagen) liquid handling system was used for pipetting 15 µl of sample mix with 5 µl of 2 µM forward and reverse primer mix per reaction with all reactions made as duplicates. Samples were amplified in Rotor-Gene Q (Qiagen) with a thermal cycle of 95 °C for 15 min, followed by 35 cycles of 95 °C 25 s, 57 °C 25 s, 72 °C 25 s, followed by a melting step. The qRT-PCR results were analyzed by the ΔΔCt method, using Cyclophilin G as a housekeeping gene. The expression data were presented as a fold change compared to the control. For the reverse transcription PCR (RT-PCR) reaction, 1 µl of synthesized cDNA was used per reaction and added to the master mix containing DreamTaq DNA polymerase (Thermo Scientific). Primers used in RT-PCR and qRT-PCR assays are listed in supplemental table S3.

Immunocytochemistry

The cells were plated onto 24-well culture plates prior to the immunostainings. Cells were fixed with 4% paraformaldehyde (PFA) (Fisher Chemical) in PBS for 15 min. The cells were then permeabilized by 0.5% Triton X-100 in PBS for 10 min and treated with Ultra Vision (UV)-blocker (Thermo Scientific) for 10 min. Primary

antibodies were diluted in 0.1% Tween in PBS, added to the wells and incubated for 24 h in dark at 4 °C on a Stuart SSL4 see-saw rocker. Secondary antibodies, and Hoechst 33342 (Thermo Fisher Scientific) to stain the nuclei, were diluted in 0.1% Tween in PBS and added to the wells. The wells were then incubated in the dark at RT for 30 min on the see-saw rocker. Primary and secondary antibodies used are listed in supplemental table S3.

Flow cytometry

Prior to the flow cytometry analysis, the cells were detached as single cells from the culture plates with TrypLE Select. 1 x 10^6 cells were resuspended in flow cytometry-buffer (5% FBS in PBS) containing 1:10 TRA-1-60 primary antibody and incubated in the dark at RT for 20 min on the see-saw rocker. After aspirating the primary antibody solution, the secondary antibody was diluted 1:100 in flow cytometry-buffer and added to the cells. Cells were incubated in the dark at RT for 20 min on the see-saw rocker, and resuspended in 500 µl flow cytometry-buffer and transferred to 5 ml Falcon flow cytometry-tubes. Cells were analyzed in FACSCalibur (Becton Dickinson) using 3 x 10^4 cells per sample. The acquired data were analyzed and visualized using the FlowJo v.10.7 software.

Karyotyping

2 x 10^6 cells were suspended in medium supplemented with 0.1 µg/mL KaryoMAX Colcemid Solution in PBS and incubated for 4 h at 37 °C. Cells were resuspended in 0.075 M KCl and incubated at 37 °C for 10 min. Fixative (3:1 ratio of methanol and acetic acid) was added dropwise to the cell suspension. Fixation was repeated three times before storing the samples at 4 °C until shipping. Karyotyping was performed as a service by Ambar in Barcelona, Spain.

Bulk RNA sequencing and processing

Total RNA was extracted from 15 iPSC lines (Supplemental table 2) and purified by NucleoSpin RNA Plus kit (Macherey-Nagel). Bulk RNA-seq was performed as a service at Novagen after the cells passed quality control. Samples were sequenced using Illumina PE150. The raw data was filtered with cutadapt (Martin, 2011) to remove adapter sequences, ambiguous (N) and low quality bases (Phred score < 25). The filtered reads were mapped to a customized version of the human reference genome (GRCh38 release 99) with STAR aligner (Dobin et al., 2013). The backbone of the plasmid sequences (pCXLE-gw) and gene annotations to the reference genome were added as a separate chromosome. Gene expression was counted from read pairs mapping to exons using featureCount in Rsubreads(Liao et al., 2019). Duplicates, chimeric and multi mapping reads were excluded, as well as reads with low mapping scores (MAPQ < 10). The read count data were analyzed with DESeq2 (Love et al., 2014). The expression profiles of the RNA-seq data were compared to published reference datasets of iPSC, LCL and fibroblasts (GSE79636, HipSci, GSE121926, E-MTAB-4652) (Carcamo-Orive et al., 2017; Kaisers et al., 2017; Kilpinen et al., 2017; Ozgyin et al., 2019) analyzed with the same methods.

Single-cell RNA sequencing and processing

Cells were dissociated with Accutase at 37 °C for 5 min, resuspended in PBS + 0.04% BSA and passed through a Flowmi tip strainer (Fisher Scientific) to yield a single-cell suspension. The quality of the samples was assessed using a Luna cell counter (Logos Biosystems). ScRNA-seq was performed using the 10x Genomics Chromium Single Cell 3'RNA-seq platform at the Institute of Molecular Medicine Finland (FIMM). The raw data were mapped to the same customized human reference genome (GRCh38.99 + pCXLE-gw) using Cell Ranger (10x Genomics, v.3.1.0). Empty droplets were removed with DropletUtils (Lun et al., 2018). The remaining droplets were filtered to exclude cells with less than 2000 detected genes, and cells with a high mitochondrial fraction (>50 %). Transcripts that were detected in less than 10 cells were also excluded. The UMI counts were analyzed with R (R Core Team, 2019) using Seurat (version 3.1.4) (Stuart et al., 2019). The data were normalized and the 1000 most variable features per sample were identified (using FindVariableFeatures). The data were scaled (using ScaleData), and the top 50 principal components were extracted. The PCA matrix was "harmonized" using Harmony (Korsunsky et al., 2019) to reduce sample specific biases, with sample ID as the main variable, theta-value set to 2, using 50 clusters and maximum iterations for clusters set to 40 and 15 for the entire harmony series. The harmonized PCA values were then used as input for a 2D and 3D UMAP. Cell clusters were identified with Seurat functions (FindNeighbors, FindClusters and BuildClusterTree) with the

resolution set to 0.04 and the rest of the settings set to default. For pseudotime analysis we used monocle 3 (v0.2.2) (Trapnell et al., 2014) in combination with tradeSeq (Van den Berge et al., 2020), as well as diffusion maps using destiny (v3.0.1) (Angerer et al., 2016). Expression scores of pluripotency genes were calculated using the AddModuleScore function of Seurat. Differential gene expression analysis between different conditions was performed with the FindMarkers function of Seurat with default settings, and genes with Bonferroni adjusted *P*-value < 0.01 (Wilcoxon rank-sum test) were considered as differentially expressed. Pearson correlation coefficients between cells were calculated using the normalized expression values of 2,000 most variable genes among the mid-reprogramming samples. Annotation of cell identity for the mid-reprogramming samples was performed using SingleR (v1.0.6) (Aran et al., 2019) with Human Primary Cell Atlas (Mabbott et al., 2013) as the reference.

Supplemental references

Angerer, P., Haghverdi, L., Büttner, M., Theis, F.J., Marr, C., and Buettner, F. (2016). Destiny: Diffusion maps for large-scale single-cell data in R. Bioinformatics *32*, 1241–1243. 10.1093/bioinformatics/btv715

Aran, D., Looney, A.P., Liu, L., Wu, E., Fong, V., Hsu, A., Chak, S., Naikawadi, R.P., Wolters, P.J., Abate, A.R., et al. (2019). Reference-based analysis of lung single-cell sequencing reveals a transitional profibrotic macrophage. Nat. Immunol. *20*, 163–172. 10.1038/s41590-018-0276-y

Balboa, D., Weltner, J., Eurola, S., Trokovic, R., Wartiovaara, K., and Otonkoski, T. (2015). Conditionally Stabilized dCas9 Activator for Controlling Gene Expression in Human Cell Reprogramming and Differentiation. Stem Cell Reports *5*, 448–459. 10.1016/j.stemcr.2015.08.001

Van den Berge, K., Roux de Bézieux, H., Street, K., Saelens, W., Cannoodt, R., Saeys, Y., Dudoit, S., and Clement, L. (2020). Trajectory-based differential expression analysis for single-cell sequencing data. Nat. Commun. *11*, 1–13. 10.1038/s41467-020-14766-3

Carcamo-Orive, I., Hoffman, G.E., Cundiff, P., Beckmann, N.D., Souza, S.D., Knowles, J.W., Patel, A., Papatsenko, D., Reaven, G.M., Whalen, S., et al. (2017). Analysis of transcriptional variability in a large human iPSC library reveals genetic and non-genetic determinants of heterogeneity. Cell Stem Cell *20*, 518–532. 10.1016/j.stem.2016.11.005

Dobin, A., Davis, C.A., Schlesinger, F., Drenkow, J., Zaleski, C., Jha, S., Batut, P., Chaisson, M., and Gingeras, T.R. (2013). STAR: Ultrafast universal RNA-seq aligner. Bioinformatics *29*, 15–21. 10.1093/bioinformatics/bts635

Kaisers, W., Schwender, H., and Schaal, H. (2017). Sample size estimation for detection of splicing events in transcriptome sequencing data. Int. J. Mol. Sci. *18,* 1-17. 10.3390/ijms18091900

Kilpinen, H., Goncalves, A., Leha, A., Afzal, V., Alasoo, K., Ashford, S., Bala, S., Bensaddek, D., Casale, F.P., Oliver, J., et al. (2017). Common genetic variation drives molecular heterogeneity in human iPSCs. Nature *546*, 370–375. 10.1038/nature22403

Korsunsky, I., Millard, N., Fan, J., Slowikowski, K., Zhang, F., Wei, K., Baglaenko, Y., Brenner, M., Loh, P. ru, and Raychaudhuri, S. (2019). Fast, sensitive and accurate integration of single-cell data with Harmony. Nat. Methods *16*, 1289–1296. 10.1038/s41592-019-0619-0

Liao, Y., Smyth, G.K., and Shi, W. (2019). The R package Rsubread is easier, faster, cheaper and better for alignment and quantification of RNA sequencing reads. Nucleic Acids Res. *47,* 1-9. 10.1093/nar/gkz114

Love, M.I., Huber, W., and Anders, S. (2014). Moderated estimation of fold change and dispersion for RNA-seq data with DESeq2. Genome Biol. *15*, 1–21. 10.1186/s13059-014-0550-8

Lun, A., Riesenfeld, S., Andrews, T., Gomes, T., and Marioni, J.C. (2018). Distinguishing cells from empty droplets in droplet-based single-cell RNA sequencing data. Genome Biol. *20*, 1-9. 10.1101/234872

Mabbott, N.A., Baillie, J.K., Brown, H., Freeman, T.C., and Hume, D.A. (2013). An expression atlas of human primary cells: Inference of gene function from coexpression networks. BMC Genomics *14,* 1-13. 10.1186/1471-2164-14-632

Martin, M. (2011). Cutadapt removes adapter sequences from high-throughput sequencing reads. EMBnet.Journal *17.1*, 10–12. 10.14806/ej.17.1.200

Ozgyin, L., Horvath, A., Hevessy, Z., and Balint, B.L. (2019). Extensive epigenetic and transcriptomic variability between genetically identical human B-lymphoblastoid cells with implications in pharmacogenomics research. Sci. Rep. *9*, 1–16. 10.1038/s41598-019-40897-9

Stuart, T., Butler, A., Hoffman, P., Hafemeister, C., Papalexi, E., Mauck, W.M., Hao, Y., Stoeckius, M., Smibert, P., and Satija, R. (2019). Comprehensive Integration of Single-Cell Data. Cell *177*, 1888-1902.e21.

Trapnell, C., Cacchiarelli, D., Grimsby, J., Pokharel, P., Li, S., Morse, M., Lennon, N.J., Livak, K.J., Mikkelsen,

T.S., and Rinn, J.L. (2014). The dynamics and regulators of cell fate decisions are revealed by pseudotemporal ordering of single cells. Nat. Biotechnol. *32*, 381–386. 10.1038/nbt.2859

Weltner, J., Balboa, D., Katayama, S., Bespalov, M., Krjutškov, K., Jouhilahti, E.-M., Trokovic, R., Kere, J., and Otonkoski, T. (2018). Human pluripotent reprogramming with CRISPR activators. Nat. Commun. *9*, 1–12. 10.1038/s41467-018-05067-x
